# Insights into the Catalytic Activity of a Metagenome-Derived Urethanase

**DOI:** 10.1021/jacs.5c13147

**Published:** 2025-11-05

**Authors:** Katarzyna Świderek, Kemel Arafet, Victor de Sousa Batista, Daniel Grajales-Hernández, Fernando López-Gallego, Vicent Moliner

**Affiliations:** 1 BioComp Group, Institute of Advanced Materials (INAM), 16748 Universitat Jaume I, Castellón 12071, Spain; 2 Heterogeneous Biocatalysis Laboratory, Center for Cooperative Research in Biomaterials (CIC biomaGUNE), Basque Research and Technology Alliance (BRTA), Donostia-San Sebastián 20014, Spain; 3 Ikerbasque, Basque Foundation for Science, Plaza Euskadi, 5, Bilbao 48009, Spain

## Abstract

The discovery of urethanases shows an opportunity to access the biotechnological recycling of polyurethane-based plastics (PURs), widely used in the manufacture of everyday materials. However, the mechanistic understanding of these enzymes remains under debate. In this work, we report a QM/MM-based mechanistic study of the metagenome-derived urethanase UMG-SP2 catalyzing the degradation of a urethane-like model compound, 4-nitrophenyl benzylcarbamate (pNC). A high-quality structural model generated with AlphaFold2, prior to the availability of the crystal structure, accurately captured the Ser-Ser-Lys catalytic triad characteristic of amidase signature enzymes. Highly accurate constant-pH nonequilibrium molecular dynamics and Monte Carlo (neMD/MC) simulations provided the full titration curve of active site Lys, explaining the need for alkaline media for the enzyme to be active. The generation of the free energy landscape, obtained by means of free energy perturbation methods with the M06-2X DFT functional describing the QM region of the full system, reveals an esterase-like three-step mechanism of UMG-SP2, i.e., acylation, hydrolysis, and decarboxylation, with all steps being kinetically feasible. Our computational results show very good agreement with experimental kinetic data, with a calculated free energy barrier of 21.2 kcal·mol^–1^ for the rate-determining step compared to 22.9 kcal·mol^–1^ derived from the experimentally measured turnover frequency (TOF). The present results also open the door for the final decarboxylation occurring in the solution after the release of the product of the hydrolysis step or within the active site. These findings provide an atomistic insight into the urethanase function and establish a robust framework for the future design of biocatalysts targeting polyurethane degradation.

## Graphical abstract

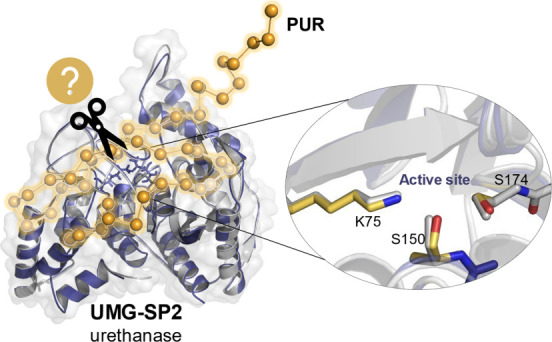

## Introduction

Plastics are one of the most important materials of the modern economy, being indispensable for food and beverage packaging, in modern medicine, textiles, construction, and beyond.^1^ In fact, plastic production has grown exponentially due to the two major advantages, the ease of mass production and resistance to degradation, and unfortunately, both advantages have become an environmental problem.^2^ Therefore, it is crucial to develop sustainable and economically viable solutions to address the growing problem of plastic pollution. As a promising alternative, biological depolymerization using highly selective enzymes offers the potential to precisely break down postconsumer plastics into well-defined building blocks. These fragments can then be repolymerized into virgin-grade materials or upcycled into high-value products.^3–5^

Polyurethanes (PURs) are versatile polymers extensively used in textiles, automotive, and packaging, contributing to a global market of over 20 million tonnes and USD 78 billion in 2023, with continued growth expected.^6^ Despite their widespread use, PURs present major recycling challenges due to their complex, often cross-linked structures, which hinder mechanical recycling and contaminate other plastic waste streams when not separated.^7,8^ PURs, like other plastics such as polyolefins,^9,10^ lack viable end-of-life solutions, making their selective depolymerization an urgent but unmet need.

Compounding the issue, PURs are derived exclusively from nonrenewable resources and, despite their C–O and C–N backbones theoretically enabling low-barrier depolymerization, current chemical and enzymatic recycling approaches are underdeveloped and require harsh conditions due to the structural complexity of PURs, including diverse bond types and thermoset formulations.

Urethanases (EC 3.5.1.75) are enzymes that catalyze the hydrolysis of urethane bonds to release alcohols, amines, and carbon dioxide.^11^ Kobashi and co-workers in 1990 reported the first urethanase isolated from *Citrobacter sp.* and found that the bacterium isolated from mouse feces stoichiometrically decomposed urethane to ethanol and ammonia.^12^ For several years, polyether-polyurethane degradation has remained inaccessible to biocatalytic hydrolysis. Recently, Bornscheuer and co-workers reported the discovery of three urethanases (UMG-SP1, UMG-SP2, and UMG-SP3) from a metagenomic library constructed using soil exposed to PUR.^13^ Their findings suggest that these enzymes are capable of hydrolyzing urethane bonds of oligomers derived from the chemical depolymerization of polyether-polyurethanes, a class of bonds previously considered inaccessible to biocatalytic hydrolysis. In particular, they demonstrated how the three metagenome-derived urethanases can hydrolyze oligomers derived from chemical polymerization of polyether toluene diisocyanate (TDI)-based polyurethane to yield aromatic diamines. UMG-SP2 is the one with the greatest potential for this purpose.^13^ Later, in a series of papers, they published the crystal structure of the three metagenomic urethanases,^14–16^ although the 3D structures were not released in the Protein Data Bank (PDB) until September 2024. They demonstrated, by structural analysis and molecular dynamics (MD) simulations with classical force fields, that the flexible loop L3 (residues from 219 to 226) is crucial for the hydrolytic activity of the enzyme. Based on sequence homology, these urethanases appear to belong to the amidase signature (AS) protein superfamily.^17–19^ In general, the catalytic triad of the AS family is formed by Lys-Ser-Ser_nuc_.^14,17,20–22^ Later on, Wu and co-workers demonstrated that substituting the Ser_nuc_ residue of the urethanase from *Agrobacterium tumefaciens d*_3_ provokes the loss of urethanase activity.^23^ Herein, the Ser_nuc_ was reported to act as a nucleophile, attacking the carbonyl carbon of the substrate, while being deprotonated by a second serine, which acts as the catalytic base.^24^ At the same time, the Ser catalytic base is deprotonated by the Lys side chain. Granzin and co-workers proposed that this second serine serves as an acid/base catalyst and is in direct contact with the substrate and the Ser_nuc_, participating in both deprotonation of the Ser_nuc_ and protonation of the leaving group during catalysis.^19^ This proposed mechanism relies on the assumption that the lysine residue is in its neutral form, enabling it to accept the proton donated by the second serine.

Recently, Ramos and co-workers investigated the reaction mechanism underlying the urethanase activity of UMG-SP2, using the diurethane ethylene 4,4′-methylenedianiline as a substrate and assuming the standard acylation-hydrolysis cycle of serine proteases.^25^ According to their findings, the first stage of the reaction corresponds to the acylation step, in which the urethane bond is cleaved through its ester moiety, releasing the alcohol leaving group. Interestingly, this step, initiated by the formation of an intermediate featuring a deprotonated Ser_nuc_, was reported to be significantly endergonic (ΔG = +10.9 kcal·mol^–1^), which may raise concerns about its feasibility under physiological conditions. Moreover, in the subsequent hydrolysis step, the reaction product is a carbamic acid intermediate, suggesting that the complete degradation of the urethane bond, i.e., formation of CO_2_ and the corresponding amine, would not be enzyme-mediated but would instead occur independently of the enzyme.

Herein, we report a study of the hydrolysis of a PUR-like compound, 4-nitrophenyl benzylcarbamate (pNC), in an attempt to model the urethane bonds occurring in aromatic polyurethane-based plastics.^13^ Despite the lack of an aliphatic alcohol moiety, frequently present in PU-based plastics,^26,27^ and the low solubility of pNC in water, the selection of pNC as a PUR model compound is mainly based on computational reasons; pNC is a small urethane surrogate that resembles the aromatic amines found in polyurethanes. Thus, the use of a small model allows for including the full molecule in the quantum region during QM/MM simulations, thereby avoiding a possible source of errors derived from, for instance, introducing additional quantum link atoms. In addition, this compound has been employed in a recent study where a variety of hydrolases (lipases, proteases, cutinases, urethanases) were screened for their ability to catalyze the transcarbamoylation of PUR thermosets in primary alcohols under mild conditions.^28^ Thus, the results and conclusions derived from the present study can be directly compared with other studies with similar final goals. Finally, as shown in Figure 1, the model compound contains an asymmetric carbamate bond that, upon hydrolysis, releases two products readily detected by UPLC-MS using the same method. Thus, this surrogate substrate facilitates us to explore both amidase and the esterase activity of UMG-SP2. Previous studies have revealed how natural enzymes, such as cutinases or some enzymes from microbial sources (bacteria and fungi), that grow using PUR as their sole carbon and energy source,^29,30^ are capable of hydrolyzing the ester bond of polyester-polyurethanes, albeit with limited efficiency, and are mostly active on the ester bonds of the polyester chains.^31–35^ However, as mentioned by Badenhorst, Bornscheuer and co-workers, the urethane bonds in polyether-polyurethanes have remained inaccessible to biocatalytic hydrolysis, and UMG-SP2 should be considered as the first true urethanase, capable of hydrolyzing urethane bonds.^13^

**Figure 1:**
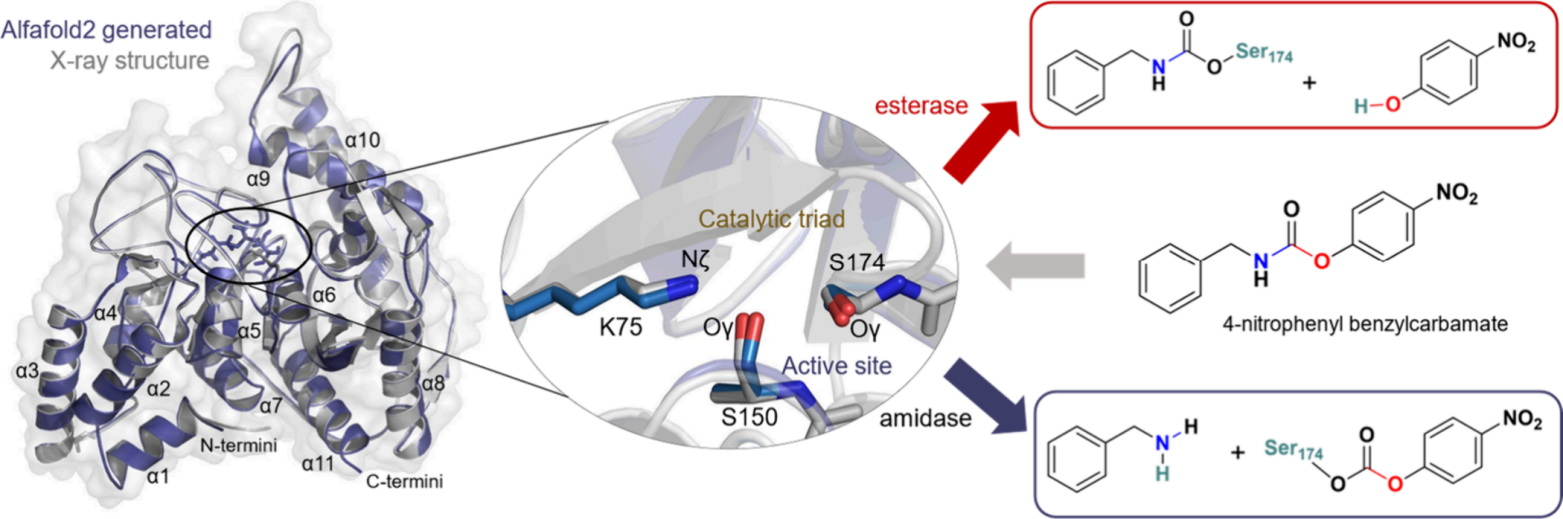
Overlay of the crystal structure of urethanase UMG-SP2 from an uncultured bacterium with PDB code 8WDW (in gray) and the structure predicted by AlphaFold (in blue). Detail of the active site triad is shown in the center, and acylation step products of the possible amidase (blue arrow) or esterase (red arrow) activity of UMG-SP2 on the 4-nitrophenyl benzylcarbamate (pNC) PUR model compound are schematically shown on the right side.

The enzymatic hydrolysis of the selected substrate has been explored based on QM/MM molecular dynamics (MD) simulations, and the proposed mechanism has been experimentally validated. The use of a nonsymmetric substrate opened the door to alternative binding poses within the enzyme’s active site, which enabled us to investigate both amidase and esterase activities, as schematically indicated in Figure 1. Free energy surfaces (FESs) have been computed employing free energy perturbation (FEP) methods, allowing a detailed description of the chemical reaction, with predicted activation free energies in agreement with results derived from experimental kinetics.

## Methods

### Model Setup

The initial protein geometry employed in the present computational study was obtained using the AlphaFold2 program^36^ with the nucleotide sequence available in GenBank (accession code OP972510), as solved by Bornscheuer and co-workers.^13^ It is important to point out that, as mentioned above, although the X-ray structure of UMG-SP2 was deposited in the PDB in 2023 (PDB: 8WDW),^15^ it was not made publicly available until the end of 2024. Moreover, the active site was not identified in the information reported in the literature when the genome was published. Consequently, we assumed the location of the active site based on homology studies with related Ser-Ser-Lys hydrolases, such as the amidase ClbL from the colibactin gene cluster (PDB: 8ES6).^18,37^ Then, the substrate pNC was built manually within the binding site using Discovery Studio Visualizer (DSV)^38^ by considering the available volume of the region, and using as a template the position of the β-ketothioester substrate bound in the amidase ClbL. As depicted in Figure 1, different orientations of the substrate were tested to explore the two possible activities of the enzyme as amidase (a pose that favors the breaking of the C–N bond of the carbamate group in the acylation step) or as esterase (a pose that favors the breaking of the C–O bond of the ester group).

In the molecular model, the missing hydrogen atoms of the protein were added at pH 8 using the tLEAP module of AmberTools program^39^ based on the p*K*_a_ values of the titratable residues, which were obtained from calculated titration curves for all ionizable amino acids using constant-pH nonequilibrium molecular dynamics and Monte Carlo (neMD/MC) simulations,^40,41^ implemented as a Tcl plugin, namdcph, for use in conjunction with NAMD ver. 2.12,^42^ as recently applied in our laboratory for the exploration of the PETase activity of CALB.^43^ The initial protonation states for neMD/MC were assigned based on results from the empirical PROPKA 3.1 program.^44,45^ Despite most of the lysine residues being protonated, according to the results derived from both methods, the active site Lys75 and Lys188 must be in their neutral protonation state at pH 8 (Figure 2, Figure S10, and Tables S1 and S6). The rest of the titratable residues appear to present the standard protonation state (Tables S1–S5 and Figures S6–S9). Interestingly, none of the amino acids exhibits a significant dependence of their protonation state on the orientation of the substrate. The predicted neutral state of Lys75 at alkaline pH is a key result, as it aligns with the accepted mechanism of Ser-Ser-Lys amidases, in which the active-site serine is activated by a nearby lysine through the mediation of a second serine residue.^46,47^ The p*K*_a_ shift observed for Lys188 can be attributed to its location within a deeply buried hydrophobic pocket. Given its distance from the active site and the occupied hydrophobic pocket, Lys188 is unlikely to influence either the reorientation of Ser174 or the overall catalytic activity of the enzyme (see Figures S11 and S12). In all, the protonation states of the titratable residues, and in particular the active site Lys75, appear to be invariant at pH values above 8.

**Figure 2:**
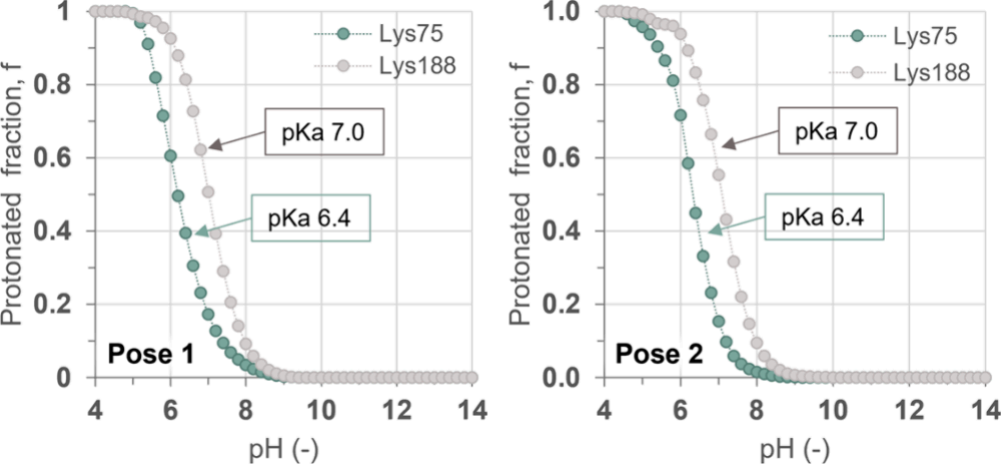
Titration curves computed for lysine residues Lys75 and Lys188 generated by pH-constant *ne*MD/MC simulations with two different poses of pNC in the active site: Pose 1 (left panel) and Pose 2 (right panel).

Missing force field parameters for the substrate were generated based on the General Amber Force Field (GAFF)^48^ and the partial atomic charges were computed at the AM1 method^49^ with bond charge corrections (AM1-BCC)^50^ using the Antechamber^51^ software (Figure S13 and Table S7). Neutralization of the system was achieved by adding 18 Na^+^ counterions in the electrostatically most favorable positions. Finally, the system was solvated into a 96 × 96 × 80 Å^3^ pre-equilibrated box of TIP3P^52^ water molecules, and counterions were added to neutralize the system.

The next step for each model consisted of 10^5^ steps of conjugate-gradient minimization, heating protocol, followed by 1 μs of molecular dynamics (MD) in the NPT ensemble with the AMBER ff03 force field,^53^ as implemented in NAMD software.^42^

In all simulations, periodic boundary conditions were applied, as well as the Particle Mesh Ewald (PME) algorithm for the electrostatic interactions with a force-switch scheme ranging from 14.5 to 16 Å, and a time step of 2 fs was used through the SHAKE algorithm.^54^ Analysis of the time evolution of the root-mean-square deviation (RMSD) of the backbone atoms of the protein and heavy atoms of the substrate confirms that the molecular systems were equilibrated after 1 μs MD simulations (Figure S14). Analysis was done using cpptraj software.^55^

### Free Energy Surfaces

A representative structure of the equilibrated system with the substrate in pose 2 generated during the MD simulations (as described in the Results section, pose 2 is the only stable conformation in the active site of the enzyme) was used as a starting point for the subsequent QM/MM computational study, using the fDYNAMO library.^56,57^ As in previous studies, the representative structure was selected from the equilibrated MD trajectory showing the key active site interatomic distances in the maxima of the population plots. The hybrid M06–2X functional^58^ with the standard 6–31+G­(d,p) basis set,^59^ were used to describe the QM subset of atoms corresponding to the catalytic triad Lys75, Ser150, and Ser174, the carbonyl groups of Gly149 and Gly173, together with the substrate. The selection of the hybrid functional and basis set was based on previous comparative analysis of the performance of this and other functionals on studies involving main-group thermochemistry, kinetics, and noncovalent interactions,^58,60,61^ and on our successful experience not only in recent studies carried out to explore the enzymatic hydrolysis of synthetic polymers such as dimers and trimers of PET-like compounds,^43,62^ and Impranil, a commercial PUR-like sample,^63^ but in the study of many different enzyme-catalyzed processes, including proteases, methyltransferases, etc.^64–68^ To saturate the valence of the QM-MM frontier atoms, 5 quantum link atoms were inserted (Figure S18). The AMBER ff03 ^53^ and TIP3P^52^ classical force fields were used to treat the protein and the solvent water molecules, respectively, as implemented in the fDYNAMO library.

A harmonic constraint was used to maintain the proper interatomic distances along the reaction coordinate, and a series of conjugate gradient optimizations and L-BFGS-B optimization algorithms were applied to obtain the final potential energy of the minimized constrained geometry. Transition state structures were optimized and characterized at the M06–2*X*/6–31+G­(d,p)/MM level, and the intrinsic reaction coordinate (IRC)^69,70^ paths were traced down to the forward and backward energy minima structures to confirm the reliability of the computational protocol. A micro–macro iteration optimization algorithm^71,72^ was used to localize, optimize, and characterize the key transition state structures (TSs) using a Hessian matrix containing all the coordinates of the QM subsystem at M06–2*X*/6–31+G­(d,p)/MM level.

The FEP method was applied to generate the free-energy profile of each step of the catalytic mechanism.^73^ FEP involves sampling the environment through the path traced by the previously calculated IRC at the M06–2X/MM level of theory. This ensures that the free-energy profile is obtained over a realistic reaction coordinate, with the MM region charges polarizing the QM wave function, thus allowing the exploration of the reaction path at a high level of theory.

The structures used in FEP calculations were obtained from the traced IRC and are characterized by a single *s* coordinate, as shown in eq 1,

$$s_{i}=s_{i-1}+{\left\{\underset{j\in QM}{\sum }m_{i}\left[{\left(x_{j,i}-x_{j,i-1}\right)}^{2}+{\left(y_{j,i}-y_{j,i-1}\right)}^{2}+{\left(z_{j,i}-z_{j,i-1}\right)}^{2}\right]\right\}}^{1/2}$$ 1

Here, *x*_*j,i*_, *y*_*j,i*_ and *z*_*j,i*_ are the coordinates for the *i*th structure and *j*th QM atom of the structure and *m*_*i*_ is the mass of the corresponding QM atoms.

The change of the free energy can be expressed as a function of *s,* as shown in eq 2,

$$\Delta G_{FEP}\left(s^{R}-s^{j}\right)=\left[E_{QM}^{0}\left(s^{j}\right)-E_{QM}^{0}\left(s^{R}\right)\right]-k_{B}T\overset{j}{\underset{i=R}{\sum }}\ln{\left\langle \exp\left[\frac{E_{QM/MM}\left(s^{i+1}\right)-E_{QM/MM}\left(s^{i}\right)}{k_{B}T}\right]\right\rangle }_{MM,i}$$ 2

Where the *E*_*QM*_^0^ is the gas-phase QM energy computed at M06–2*X*/6–31+G­(d,p) level, *k*_*b*_ the Boltzmann constant, and T the temperature. The contribution of the QM/MM interaction to the free energy difference between two distinct values of *s* is obtained by averaging the QM/MM interaction energy, including the polarization energy, over all the MM coordinates obtained for the said *s* coordinate during the MD simulation. In this case, 20 ps of QM/MM NVT MD simulations were carried out for each window along the IRC path at 303 K, with the QM atoms frozen. Finally, the contribution of the vibrations of the QM subsystem to the free energy was obtained, considering the quantum nature of its motions under the harmonic approximation, for the stationary points.

### Kinetic Studies

To perform kinetic studies of the hydrolysis of pNC, 1 mL of a mixture composed of 1 mM of 4-nitrophenyl benzylcarbamate in DMSO at 10% in the presence of 1% (v/v) of Triton X-100 in buffer sodium bicarbonate at 100 mM and pH 10, was incubated with 0.77 mg of UMG-SP-2 (immobilized on porous agarose 6BCL beads functionalized with cobalt-chelates through its His-tag) at 30 °C and 1000 rpm Blanks without the enzyme were carried out. Samples were taken at 1.5, 3, 19, 48, and 120 h. Samples were diluted 10-fold in acetonitrile and analyzed using an Acquity UPLC equipped with a photodiode array detector (PDA) and an Electrospray Ionization Time-of-Flight mass spectrometry detector (ESI-TOF) LCT Premier XE from Waters. The gradient elution buffers were (A) 1% trifluoroacetic acid and (B) acetonitrile. The following gradient program was used at a 0.3 mL min^–1^: from 0 to 1 min, isocratic at 99% A; from 1 to 5 min, gradient to 1% A; from 5 to 7 min, isocratic at 1% A, from 7 to 9 min back to 99% A and stabilization for 1 min. Retention times for pNC, 4-nitrophenol, and benzylamine were 5.06, 2.90, and 3.3 min, respectively. All chemicals and reagents were of analytical grade purchased from Sigma (St. Louis, IL).

To study the effect of Triton X-100 on the UMG-SP2 activity, 5 μL of a solution of UMG SP2 at 8 μg/mL were placed in a 96 well microplate, afterward 200 uL of a solution composed of 0.5 mM of 4-nitrophenyl butyrate, 10% of dimethyl sulfoxide and increasing concentrations of Triton X-100 in buffer sodium phosphate 50 mM at pH 7.0, were added to start the reaction. Reaction progress was monitored spectrophotometrically at 348 nm. The hydrolysis rate was calculated using the extinction coefficient of 4-nitrophenol at 348 nm and pH 7.0. (5.4 mM^–1^cm^–1^).

## Results and Discussion

### Generation of UMG-SP2 3D Structure

The first step in our computational study was to compare the 3D structure of the protein predicted by AlphaFold, which served as the initial model in this work, with the recently released crystal structures of UMG-SP2 (PDB: 8WDW and 8XTB), where the enzyme is complexed with phenylmethanesulfonyl fluoride, and with 4-oxidanylbutyl ∼ {N}-(4-aminophenyl)­carbamate, respectively. In addition, we also compare the predicted structure with the amidase ClbL from the colibactin gene cluster (PDB: 8ES6).^18,37^ The structural alignments between the AlphaFold-predicted model and the three crystal structures are remarkably high, including not only the overall protein backbone but the position of the catalytic triad in the predicted and the crystal structures (see left and center panels of Figure 1 and Figure S1), despite the sequence homology between UMG-SP2 and amidase ClbL is not high (34% as shown in Figure S5). In fact, the similarities between ClbL and the UMG-SP2 crystal structures were recently highlighted when the structure of UMG-SP2 was published.^15^ The root-mean-square deviations (RMSDs) calculated for the protein backbone atoms and all heavy atoms between the AlphaFold-predicted model and the UMG-SP2 crystal structure (8WDW) are 0.629 Å and 2.648 Å, respectively, revealing a high similarity between the two systems. However, the calculation of the RMSD by residue reveals some differences (RMSD > 3 Å) in flexible irregular segments of amino acids, loops 310–320 and 200–210, being Ser206, Ala207, and Lys208 those with significantly different orientations of their side chain (Figure S2). These two loops are exposed to the solvent, thus suggesting a certain degree of flexibility, which is mirrored by the high crystallographic B-factor found for these regions (Figure S3). These results support the reliability of the structural models generated from the AlphaFold2 prediction, which were used as the foundation for our simulations. Once the protein was set up, and after adding the H atoms according to the estimation of the p*K*_a_ of the titratable residues, we explored the protein:substrate binding of the selected compound. At this point, it is important to point out that the location of the active site was not evident when the protein structure was generated from its annotated protein sequence.^13^ Then, as mentioned in the methods section, by analogy with the active site of amidase ClbL, a solvent accessible cavity where a conserved Ser-Ser-Lys triad was identified (Ser174, Ser150, and Lys75, UMG-SP2 numbering). This agrees with the structural analysis later carried out with the UMG-SP2 solved crystal structure.^15^

### Substrate: Protein Binding Step

Once the protein was generated, the next step consisted of manually docking the substrate into the active site of the enzyme. Thus, the substrate can adopt two different promising poses in the active site, Pose 1 and Pose 2 (Figure 3A). Consequently, long MD simulations were carried out to confirm the stability of both complexes. The difference between the two poses relies on the relative orientation of the carbamate group of the substrate to the catalytically active site Ser174. According to the results, while the substrate pNC remains stable in the active site when adopting Pose 2, it is spontaneously released from the binding pocket to the solvent after ca. 400 ns when starting the MD simulations from the alternative Pose 1 (see Figure 3B). It is important to stress that, according to the interatomic distances between the substrate functional group and the nucleophilic Ser174, the orientation of the substrate in Pose 2 would promote the breaking of the ester bond in the acylation step, while the alternative Pose 1 would promote the initial breaking of the C–N bond (see Figure 1). Interestingly, the similarity between the conformation of Pose 2 of pNC in the active site of the protein structure generated by AlphaFold and the pose of the original ligand 4-oxidanylbutyl ∼ {N}-(4-aminophenyl)­carbamate employed during the crystallization of UMG-SP2 (PDB: 8XTB) is remarkable (see Figure S1).

**Figure 3:**
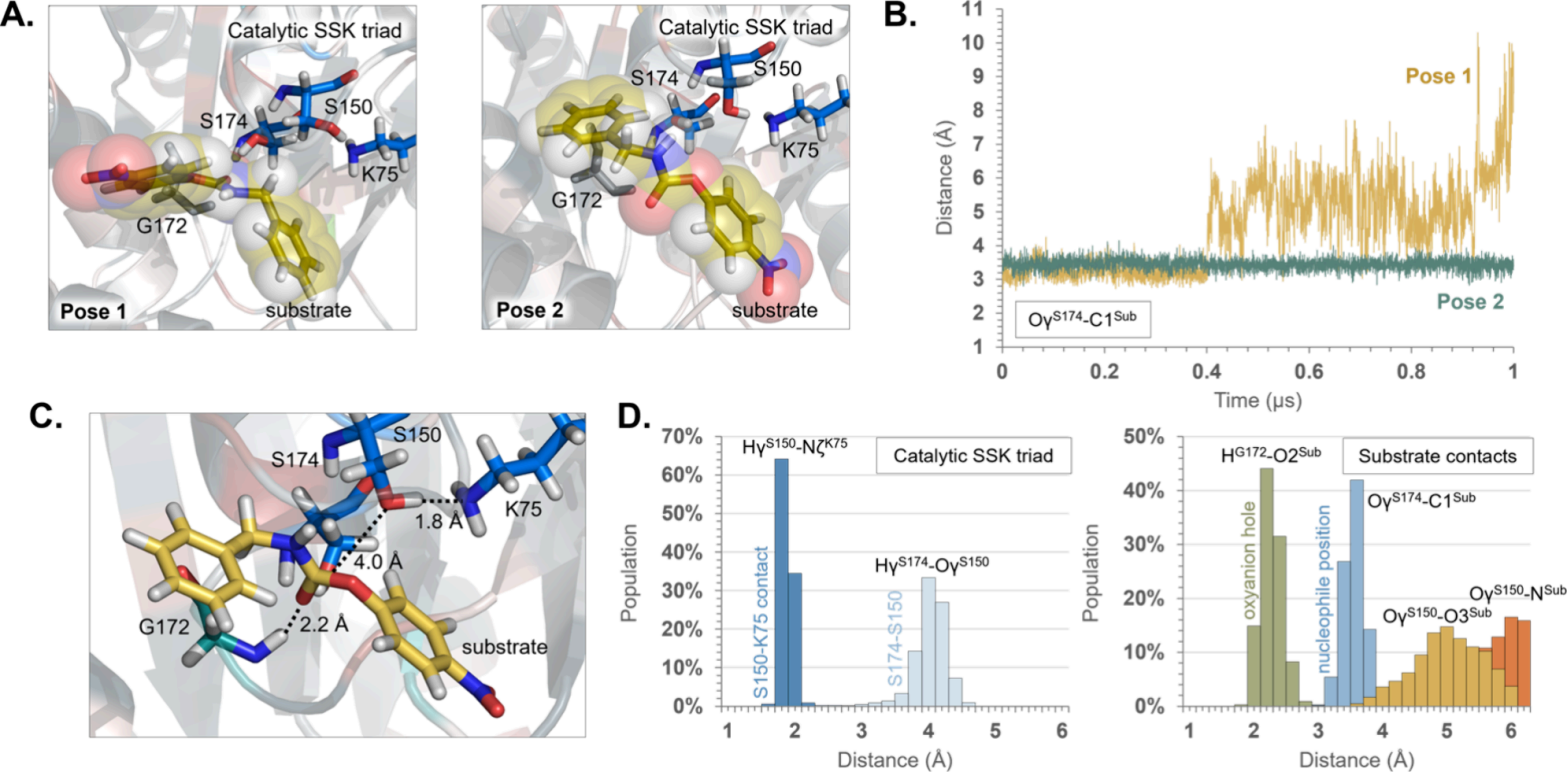
Structural analysis of the initial protein: substrate complexes derived from 1 μs MD simulations. A. Detail of the active site of structures with the two possible poses of the substrate, depending on the relative position between the carbamate group and the catalytic SSK triad (Pose 1 and Pose 2). B. Time evolution of the interatomic distance O^γ(S174)‑^C1^(Substrate)^. C. Representative structure of the equilibrated system in Pose 2. Key distances are given in Å. D. Population analysis of interatomic distances defining the key interactions between the substrate and the active site residues.

These results suggest that UMG-SP2 shows a certain degree of selectivity since the relative position of the active site triad and the putative scissile bonds of the substrate during the acylation step (C–O or C–N, see Figure 1) in the stable Pose 2 suggests an esterase activity of UMG-SP2. Deeper structural analysis of the protein:substrate complex derived from the MD simulations when pNC adopts Pose 2 renders remarkable results (Figures 3C and 3D). Thus, according to the assumed mechanism in the AS superfamily, some interatomic distances (i.e, Hγ^S174^-N_ζ_^K75^ and the H^G172^-O2^Sub^) appear to be favorable for the reaction to take place. These distances define the interaction network that forms the oxyanion hole. This network also includes Ile171, Gly173, and Ser174, yet their distances to the oxygen (O2^Sub^) from the substrate are always larger than those established with Gly172. These larger distances suggest weaker interactions (see Tables S19–S21). Regarding the nucleophilic Ser174, it is not well oriented for a possible proton transfer to Ser150 (Hγ^S174^-O1^Sub^ = 4 Å) despite the interatomic distance for the nucleophilic attack on the C1 atom of the substrate could suggest a reactive structure (Oγ^S174^-C1^Sub^ = 3.5 Å). Ser150 is also too far from the substrate for playing the role of the nucleophile, thus discarding an alternative mechanism where Ser174 was not involved in the reaction (Figure S16).

### Activation of the Ser174-Ser150-Lys Catalytic Triad

As concluded from the structural analysis based on the data depicted in Figure 3, the most stable protein:substrate complex adopts a nonreactive conformation (Figure 3C), requiring a preliminary conformational rearrangement before the onset of the chemical steps.

As explained in the methods section, the free energy landscape was explored by means of FEP methods from stationary point structures of transition states optimized at M06–2X/MM level, followed by tracing down the IRC path to confirm that the located TS connects the expected minima. According to the results, this preliminary activation of the Ser174-Ser150-Lys75 catalytic triad involves the rotation of the hydroxyl group of Ser174, from a nonreactive conformation (stabilized by a hydrogen bond interaction with the C=O carbonyl group of the substrate, as shown in Figure S17) to a conformation where it is pointing toward the Oγ of Ser150 (“ES inactive” to “ES active” in Figure 4). The time-dependent evolution of the dihedral angle describing the orientation of Ser174 provides the most populated conformation corresponds to the ES inactive, despite ES active conformations being sometimes observed (Figure S17). This result suggests an equilibrium with a low energy barrier, as confirmed below. In this regard, conformational effects associated with the formation of a reactive conformation, albeit primarily related to substrate conformational changes, were proposed in previous computationally and experimentally well-characterized fatty acid amide hydrolase containing a similar catalytic Lys-Ser-Ser triad.^74^ It is important to point out the required cis-orientation of Ser150, which is maintained during the MD simulations, for the formation of a reactive catalytic triad allowing the proton shuttle from Ser174 to Lys50. This has been previously concluded as a structural feature of AS superfamily members, such as the ClbL crystal structure reported by Tripathi et al.^37^

**Figure 4:**
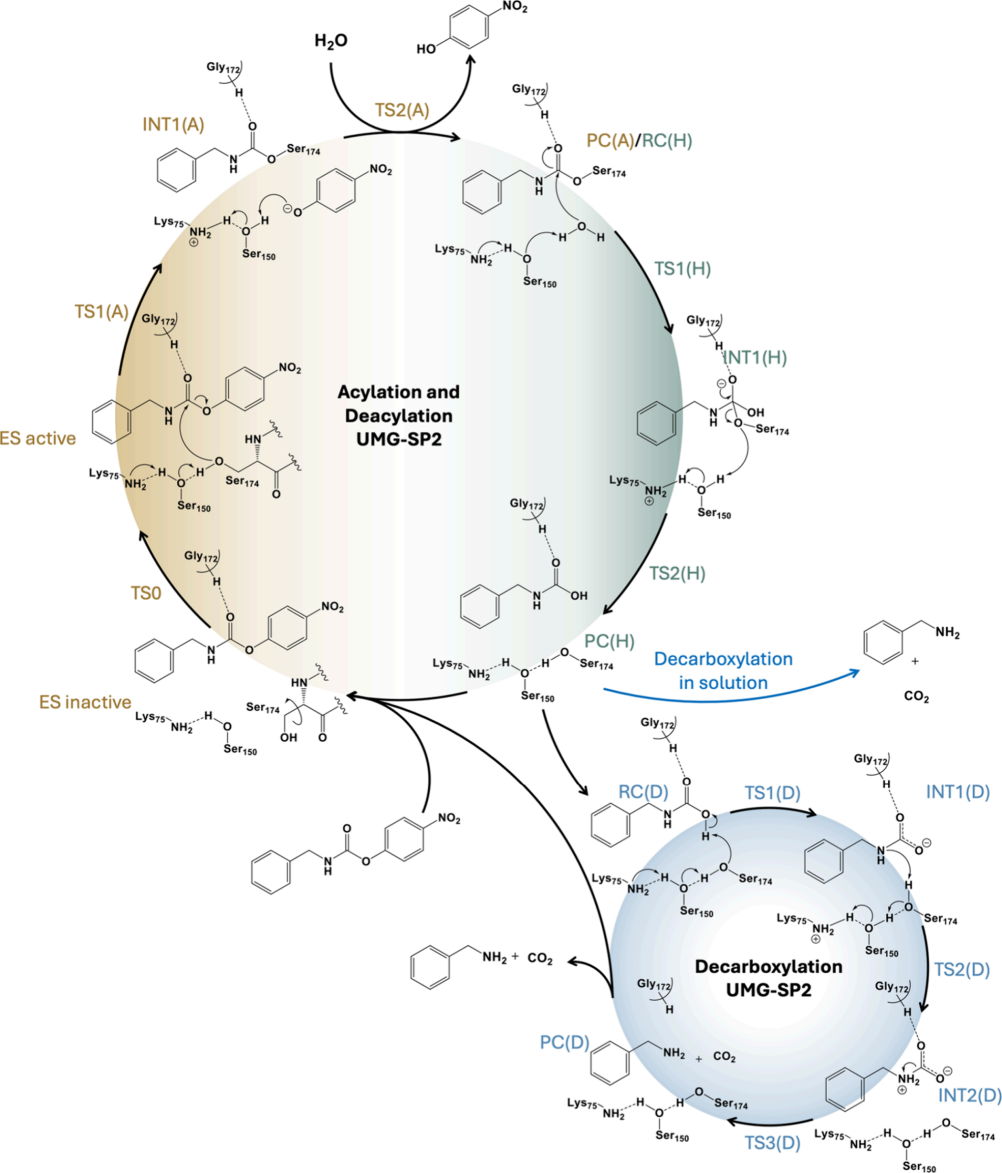
Proposed mechanism for the full decomposition of pNC catalyzed by UMG-SP2 as derived from computed M06–2X/MM free energy landscape.

According to the computed free energy profiles (Figure 5), the active ES state is just 2.6 kcal·mol^–1^ less stable than the most populated nonreactive conformation, separated by a free energy barrier of 4.5 kcal·mol^–1^. Thus, this conformational equilibrium may take place almost spontaneously at the working temperature. The active site of the reactive E:S shows a perfect alignment for the conventional mechanism for this type of hydrolase, where a proton shuttle takes place from Ser174 to Lys75 through Ser150 (Figure 4). This result is in contrast to previous studies, suggesting that the activation of Ser174 requires a preliminary proton transfer step to generate a metastable deprotonated serine.^25^ Our results suggest that this unfavorable chemical step is not required, but a low-energy barrier conformational rearrangement is enough to generate an active ES reactant state (see Figures 4 and 5). In general terms, as we recently discussed in a minireview,^67^ the free energy landscape, FEL, of catalyzed processes and in particular of the step corresponding to the formation of the Michaelis complex, can be seen as a dynamic entity that can be modified by external stimulus, such as the temperature, the addition of an allostery ligand, alterations of the pH or salt concentration. The results obtained in the present study suggest that a possible strategy for the development of more efficient enzymes for plastic recycling can be focused on reshaping the topology of the FEL to increase the population of more reactive reactant conformations, “ES active” in Figures 4 and **5**, or new enzyme–substrate stable conformations advanced along the reaction coordinate (Transition Like Conformations).^67,75^ A favorable reshaping of the FEL can be achieved by introducing mutations in the protein, or perhaps by less sophisticated changes in the media, such as the pH (we recently have shown how modulating the pH conditions in CALB can selectivity switch the formation of different subproducts from PET oligomers)^43^ or the temperature. In this regard, studies on the temperature dependence of enzyme activity, a subject of increasing interest and controversy,^75–78^ have important implications for this field.

**Figure 5:**
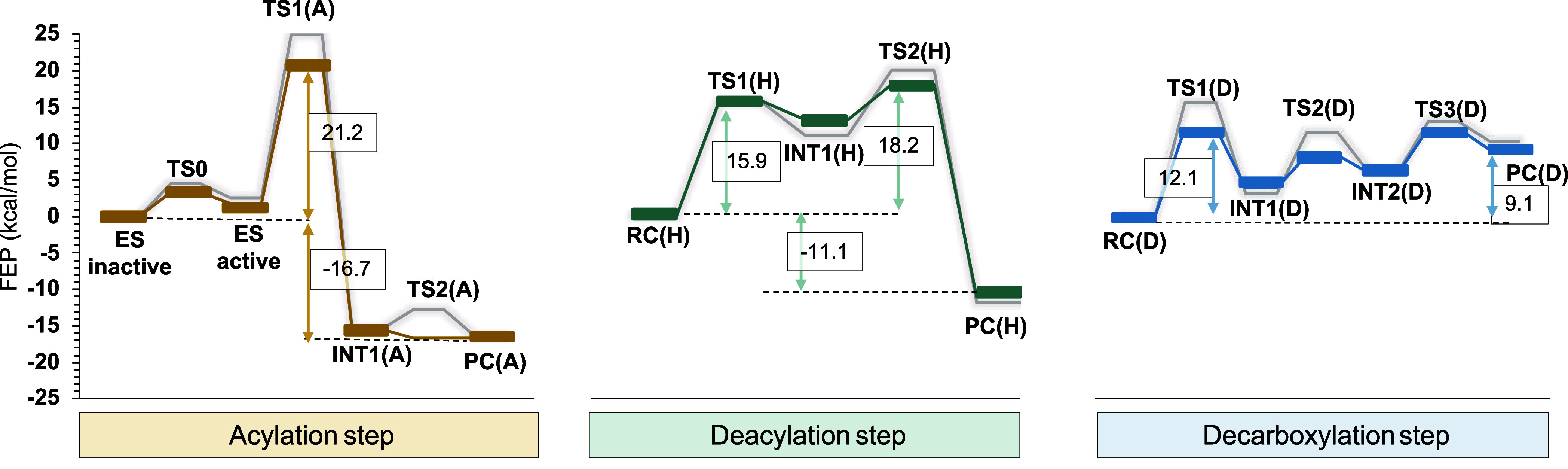
M06–2*X*/6–31+G­(d,p)/MM free energy profile of the catalytic mechanism of the substrate 4-nitrophenyl benzylcarbamate hydrolysis catalyzed by UMG-SP2. The orange, green, and blue lines represent the free energy profile for acylation, deacylation, and decarboxylation steps. The gray lines represent the free energy profiles before adding the zero-point vibrational corrections. FES of each step is deposited in the Supporting Information, Figures S19–S21, while energetic values are reported in Tables S8–S10, and estimations of statistical errors in Tables S11–S18.

### Chemical Reaction

The next step of our study consists of the exploration of the chemical steps of the hydrolysis of the selected compound catalyzed by UMG-SP2. According to the results derived from the exploration of the binding of pNC in the active site of UMG-SP2, the full catalyzed reaction was explored from the stable protein: substrate, Pose 2.

Once the system arrives at a reactive noncovalent reactant complex, we propose that the complete biocatalytic degradation of the PUR-like compounds will follow a molecular mechanism similar to those shown in other Ser-Ser-Lys hydrolases, consisting of two steps: acylation and hydrolysis (Figure 4). In addition, we will explore the possibility of a third step, decarboxylation, taking place in the active site of the enzyme. This is an alternative to previous studies suggesting that the complete degradation of the urethane bond, i.e., formation of CO_2_ and the corresponding amine, would occur independently of the enzyme.^25^

#### Acylation

The deprotonation of Ser174 takes place concomitant with the nucleophilic attack on the carbonyl carbon of the carbamate group of the substrate and, interestingly, the C–O scissile bond. This step appears to be exergonic for pNC (−17.91 kcal·mol^–1^). The acylation step is completed by the release of the leaving group from the active site after a proton transfer from Ser150, which is assisted by Lys75. The complete acylation step appears to be thermodynamically favorable with a reaction free energy of −16.7 kcal·mol^–1^ and an activation free energy of 21.2 kcal·mol^–1^ (measured from the most stable inactive reactant state, ES).

#### Hydrolysis or Deacylation

Once the acylation was finished, the nitrophenyl alcohol leaving group was replaced by 4 water molecules within H-bond distance in way that one of them act as a nucleophile for the hydrolysis step, once activated by Ser150 and Lys75. The number of water molecules was selected according to the volume of the cavity generated once the leaving group is released from the active site (Figure S23). As depicted in Figure 4, proton transfer from the water molecule to the catalytic dyad proceeds simultaneously with the formation of the bond between the substrate’s carbonyl carbon and the water oxygen, resulting in intermediate INT1­(H). The subsequent deacylation step yields the hydrolysis product, PC­(H), corresponding to a carbamic acid species. The hydrolysis, an exergonic process with a free energy change of –11.1 kcal·mol^–1^, is kinetically controlled by the second step, which involves cleavage of the covalent bond between Ser174 and the substrate, accompanied by a double proton transfer from Ser150 to Ser174 and from Lys75 to Ser150. This step proceeds with an activation free energy of 18.2 kcal·mol^–1^. Finally, because the presence of water networks in the active site of serine hydrolases can dominate barrier heights,^79,80^ we have monitored the water occupancy and orientation statistics in the active site across the deacylation steps. Our analysis shows that the number of water molecules in the active site remains constant throughout the deacylation steps, although some molecules exchange with the bulk solvent due to the open cavity of the active site being exposed to the solvent. (see Table S23 and Figures S23 and S24).

#### Decarboxylation

The product of the hydrolysis step, a carbamic acid, can be released from the active site and decompose in solution through a spontaneous decarboxylation process, giving CO_2_ and an amine, as previously suggested by Ramos and co-workers.^25^ However, because MD simulations show that the noncovalent product complex of hydrolysis, PC­(H), remains stable in the active site (Figure S22), we explored the possible decomposition of the product of the hydrolysis in the active site of the enzyme, thus, completing the full degradation of the initial compounds. Structural analysis of the average structures suggests that the active site of the enzyme provides an environment, basically based on the restored triad, that can indeed catalyze this last step of the reaction. This last scenario was explored assuming the proposed mechanisms shown in Figure 4. According to the free energy profile, shown in Figure 5, the decarboxylation step catalyzed by UMG-SP2 presents a low activation free energy (12.1 kcal·mol^–1^), suggesting the feasibility of the catalyzed process. However, from the thermodynamic point of view, the formation of the carbamic acid and CO_2_ in the active site of the enzyme would not be a spontaneous step, considering its endergonic character (9.1 kcal·mol^–1^). Considering that the rate constant for the spontaneous decarboxylation of benzylcarbamic acid in solution has been reported as 49 s^–1^ at 25 °C,^81^ which corresponds to an activation free energy of 15.1 kcal·mol^–1^ based on TST, our results open the door for different possible scenarios, with or without participation of the enzyme in the final decarboxylation of the product of the hydrolysis step, the former being kinetically more favorable, albeit thermodynamically endergonic.

In all, according to our computational simulations, the complete free energy landscape of the degradation of the pNC catalyzed by UMG-SP2 shows the feasibility of the process, despite predicted low activity according to the activation energy, 21.2 kcal·mol^–1^, of the rate-limiting step, TS1­(A). This prediction is supported by the experimental kinetic results detailed below, considering the uncertainty associated with the computational and experimental methods. In this regard, the estimated error in the energy barriers for each chemical step, calculated using the multivariable error propagation method along the individual FEP paths, reaches values of up to 0.97 kcal·mol^–1^ (see Supporting Information for details). Notably, this value is consistent with previous estimations of the error associated with the employed methodology which, based solely on the statistical uncertainty from MD sampling, is assumed to be around 1 kcal·mol^–1^.^82–84^

### Kinetic Studies

To support the computational mechanism, we next validated UMG-SP2 activity toward pNC (**1**) experimentally. We performed the hydrolysis of pNC (Figure 6A) under the conditions stated in the methodology section in triplicate. Because of the poor solubility of pNC in water, a cosolvent (10% DMSO) and a detergent were added to the reaction media to enhance the substrate solubility up to 1 mM. Under these conditions, the release of benzyl amine was followed through time using UPLC-MS because of the limitations of conventional UV–vis assays. Figure 6B shows the chromatograms (λ = 240 nm) of the p-nitrophenol (**3**) and benzylamine (**2**) standards, as well as the reaction catalyzed by UMG-SP2 at pH 10. This reaction pH was selected according to the activity-pH profile of this enzyme previously reported by Branson et al.^13^ Furthermore, we performed the reaction at pH 10 to ensure that the catalytically relevant Lys 75 is unprotonated considering the *in silico* titration curves (Figure 2). As expected, we detected **2** when the reaction was performed at basic conditions. According to the proposed mechanism (Figure 4), Lys75 must be deprotonated to accept the proton from Ser150 and then activate Ser174 for the nucleophilic attack in the first step of the acylation. In agreement with the data reported by these authors using 7-carbethoxy-4-methylcoumarin, we also found that UMG-SP2 hydrolyzes pNC more efficiently at alkaline pH (pH = 10). Under these conditions, we observed a plateau after 18 h of reaction (Figure 6C). Even incubating the enzyme for longer times (120 h), we found no further increase in the benzyl amine concentration, which reached a maximum value of 40 μM. The amine titers upon the enzyme-driven hydrolysis of this urethane surrogate are higher than the titers reported for the same enzyme using other model substrates.^85^ We suggest that the premature stop of the reaction at very low product concentrations is due to enzyme inactivation triggered by the drastic reaction conditions (10% DMSO, 1% Triton at pH 10). By assessing the concentration of benzylamine released into the reaction media, we determined the specific activity of the UMG-SP2. Herein, we found a maximum turnover frequency (TOF) of 1.77 ± 0.84 x10^–4^ s^–1^ in the first 3 h, reaching a total turnover number (TTN) value after 120 h of 5.25. Despite Michaelis–Menten parameters could not be determined due to the low solubility of the substrate under the reaction conditions, considering the TOF as an apparent *k*_*cat*_, the obtained value corresponds to a phenomenological free energy barrier of 22.9 ± 0.3 kcal·mol^–1^, as estimated using transition state theory (TST) at 303 K. This value shows remarkably good agreement with the computationally predicted barrier of 21.2 kcal·mol^–1^, obtained for the rate-determining transition state TS1­(A). The slight deviation is likely due to the presence of some additives (i.e, DMSO and Triton X-100) in the reaction media. For instance, Triton-X100 inhibits the activity of UMG-SP2 (Figure S25), which can underestimate the experimental apparent *k*_*cat*_ herein reported.

**Figure 6:**
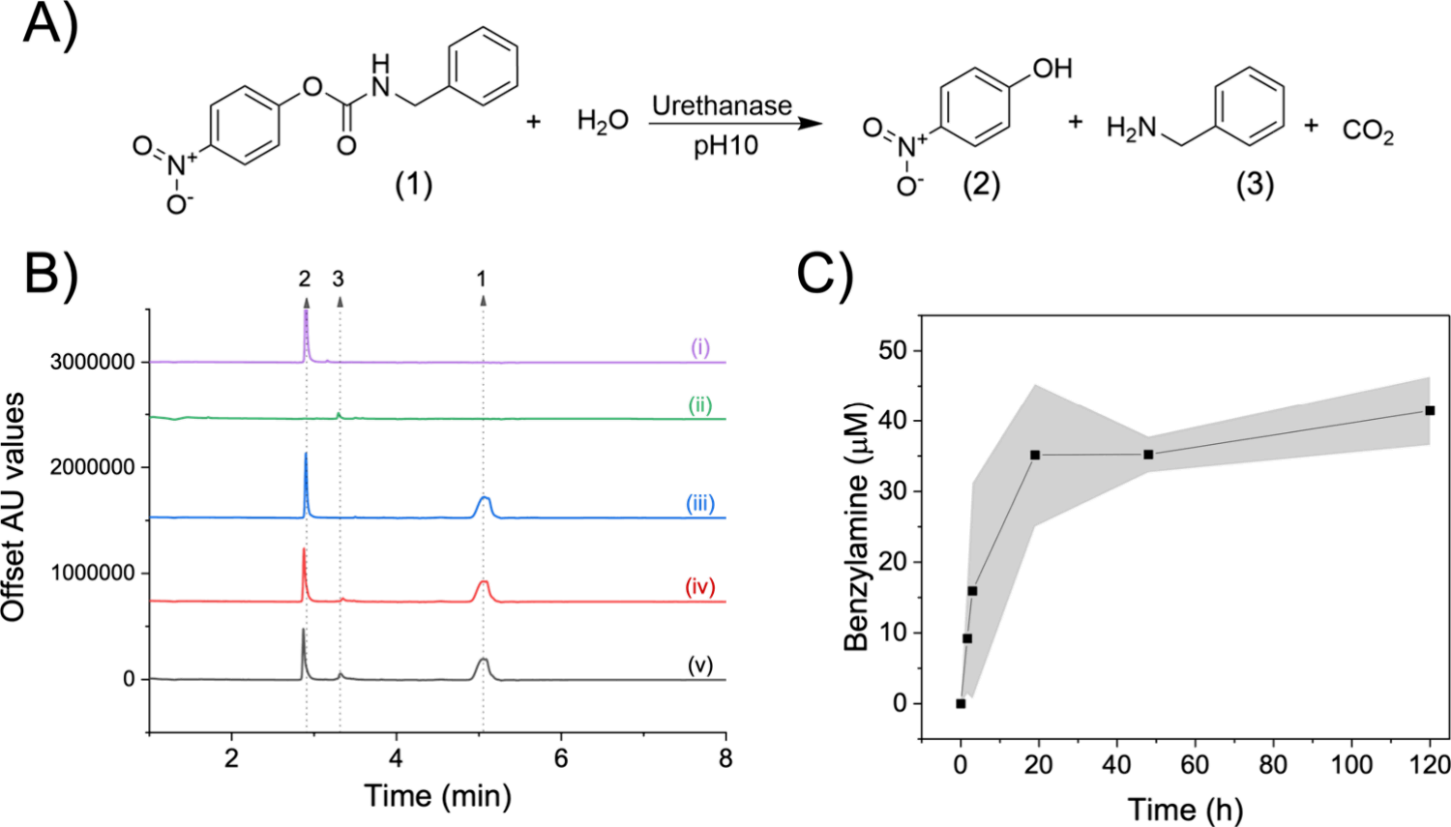
Kinetic studies in the hydrolysis of pNC driven by the UMG-SP2. A) Reaction scheme of the hydrolysis reaction. (1) 4-nitrophenyl benzylcarbamate, (2) 4-nitrophenol, and (3) Benzylamine. B) UPLC/UV–vis analysis of standards and reaction crudes resulting from the incubation of pNC, (i) Standard of 4-nitrophenol, (ii) standard of benzyl amine, (iii) Initial reaction mixture of hydrolysis (T_0_). (iv) Reaction at 90 min. (v) Reaction at 120 h. Arrows and numbers in the upper figure indicate the compound detected as referenced in A. C) Reaction course of the hydrolysis of pNC driven by the UMG-SP2. Reaction conditions: 1 mM of 4-nitrophenyl benzylcarbamate, dissolved in 10% of DMSO and 1% of Triton X-100, 30 °C and 1000 rpm at pH 10 in buffer sodium bicarbonate. Activity measurements were performed in triplicate. The gray shadow corresponds to the standard error of each data point.

## Conclusions

In this work, we have investigated the full catalytic mechanism of the metagenome-derived urethanase UMG-SP2 ^13^ acting on a polyurethane-like model compound, 4-nitrophenyl benzylcarbamate (pNC). Importantly, this study was initiated before the crystal structure of UMG-SP2 had been made publicly available. By generating a structural model using AlphaFold, we were able to construct and validate a reliable enzyme scaffold that closely aligned with the amidase ClbL from the colibactin gene cluster, a representative of the AS protein superfamily featuring the canonical Ser-Ser-Lys catalytic triad. Remarkably, the AlphaFold-predicted structure also showed excellent agreement with the UMG-SP2 crystal structure subsequently deposited in the PDB,^15^ highlighting the value of predictive modeling for enzyme characterization and design.

Highly accurate constant-pH nonequilibrium molecular dynamics and Monte Carlo (neMD/MC) simulations provided the full titration curve of active site Lys75, explaining the need for alkaline media for the enzyme to be active, a result that was confirmed by our kinetic studies.

After exploring potential binding poses of the model compound within the enzyme’s active site, we performed molecular dynamics simulations employing both classical and hybrid QM/MM potentials to dissect the catalytic process at atomic resolution. Our results show that UMG-SP2 promotes full substrate degradation through a three-step esterase-like mechanism involving acylation, hydrolysis (deacylation), and decarboxylation.

A particularly noteworthy finding is that the catalytic nucleophile, Ser174, does not require prior deprotonation to initiate the reaction, a departure from conventional Ser-Ser-Lys hydrolase mechanisms that suggests a more concerted proton-shuttling mechanism involving the catalytic triad. Instead, our simulations suggest that its activation is achieved by a rotation that allows achieving a reactive conformation to initiate by a concerted proton-shuttling mechanism within the Ser-Ser-Lys triad, deviating from conventional activation models in this enzyme class. This result suggests that a possible strategy for the development of more efficient enzymes for plastic recycling can be focused in reshaping the topology of the FEL to increase the population of the reactive reactants conformations, ES active, by introducing specific mutations or by external stimulus such as the temperature, the addition of an allostery ligands, and/or alterations of the pH or salt concentration.

Regarding the decarboxylation step, previous studies have proposed that the decarboxylation of the carbamic acid intermediate occurs spontaneously in solution. In contrast, our structural analysis and the fact that the product of the hydrolysis remains stable in the active site of the enzyme indicate that this final step could also be catalyzed within the enzyme’s active site since. Our results show that the enzyme-catalyzed decarboxylation step has a low activation free energy, making it competitive with the corresponding reaction in solution. However, this step exhibits an overall endergonic reaction profile, suggesting a nonspontaneous process. Analyses of our results reveal that the enzyme stabilizes key intermediates (including the product of the hydrolysis) and transition states along the reaction pathway, with each step being energetically feasible under physiological conditions.

These mechanistic nuances have significant implications for our understanding of enzymatic carbamate hydrolysis and may inform the design of engineered variants with enhanced performance, with special focus on the pH, according to the dramatic effect confirmed by our simulations and experiments. Importantly, the complete free energy landscape of the degradation of the pNC catalyzed by UMG-SP2 shows the feasibility of the process, although we predict a low activity according to the activation energy, 21.2 kcal·mol^–1^, of the rate-limiting step, TS1­(A), in water. The enzyme activity through this model substrate for urethane hydrolysis has been proven experimentally, detecting the hydrolysis product and validating the computationally proposed hydrolytic mechanism. Although the low solubility of pNC in water limited substrate availability, the experimentally measured turnover frequency (TOF), interpreted as an apparent *k*_*cat*_, corresponds to a free energy barrier of 22.9 kcal·mol^–1^ based on transition state theory at 303 K. The slight discrepancy between experimental and theoretical results likely arises from an underestimation of the enzyme’s maximum catalytic rate under the experimental conditions, as substrate saturation was not achieved. Anyway, further studies with different PUR model compounds, as those selected in previous studies, will contribute to obtaining a complete scenario of the activity of this and other putative PURases.

In sum, the present work not only sheds light on the catalytic versatility of UMG-SP2, which appears to work as an esterase with the urethane model herein selected, but also establishes a robust computational pipeline for characterizing new biocatalysts even in the absence of structural data. These insights will be instrumental in guiding the rational engineering of urethanases for more effective enzymatic recycling of polyether-polyurethane materials, polymers that have so far resisted sustainable degradation solutions.

## Supplementary Material









## Data Availability

As presently no standard database for MD data exists (see ref 86 for a discussion), reduced trajectories (5000 snapshots of each 1 μs MD data has been placed at Zenodo: https://zenodo.org/records/17208566.

## Notes

Structural data and analysis of the AlphaFold model of UMG-SP2, the crystal structure of UMG-SP2, and the crystal structure of amidase ClbL from the colibactin gene cluster. Force field parameters for pNC. p*K*_a_ values of titratable residues computed with PROPKA3.1, and titration curves derived from pH-constant neMD/MC simulations. Detail of the active site and QM-MM partitioning. M06–2*X*/6–31+G­(d,p)/AMBER FESs of every chemical step. Uncertainty analysis of free energy barrier predictions. Analysis of MD trajectories. Key distances of structures optimized at M06–2X/AMBER level (PDF)
Initial structures (in PDB format) of the MD simulations, and coordinates of structures of all intermediates, transition states, reactants, and products of the individual reaction steps, optimized at M06–2*X*/6–31+G­(d,p)/AMBER level (ZIP)
