# Supplementary material for: Insights into the Catalytic Activity of a Metagenome-Derived Urethanase
Source: J Am Chem Soc. 2025 Nov 5;147(46):42511–23. doi: 10.1021/jacs.5c13147 (PMC12636025; doi:10.1021/jacs.5c13147)
Supplement: Supplementary file 1 [file ja5c13147_si_001.pdf]

Supplementary Information

Insights into the Catalytic Activity of a Metagenome-Derived Urethanase

Katarzyna Świderek,[1,]* Kemel Arafet,[1] Victor de Sousa Batista,[1] Daniel Grajales-Hernández,[2] Fernando López-Gallego[2,]* and Vicent Moliner[1,]*

1. BioComp Group, Institute of Advanced Materials (INAM), Universitat Jaume I, 12071 Castellón, Spain.
2. Heterogeneous Biocatalysis Laboratory, Center for Cooperative Research in Biomaterials (CIC biomaGUNE), Basque Research and Technology Alliance (BRTA), Donostia San Sebastián, Spain

INDEX:

Overlay of the structures of the unusual amidase of ClbL, and UMG-SP2, with the AlphaFold model of UMG-SP2 2

Sequence alignment of UMG-SP2 crystal structure and the model generated using AlfaFold2 ... 5

Sequence alignment of amidase ClbL and model generated using AlfaFold2 6

Results for pKa derived from PROPKA ver. 3.1 program 7

Results for pKa based on constant-pH neMD/MC simulations 8

Values of pKa computed for aspartic acid residues 8

Values of pKa computed for glutamic acid residues 9

Values of pKa computed for histidine residues 10

Values of pKa computed for cysteine residues 11

Values of pKa computed for lysine residues 12

Position of Lys188 concerning the catalytic triad in the active site 13

FF parameters for the 4-nitrophenyl benzylcarbamate substrate 15

RMSD for heavy atoms of protein backbone and substrate 17

Evolution of key distances in Pose 1 17

Evolution of key distances in Pose 2 18

Schematic representation of the active site 19

Free energy profiles computed at the M06-2X/AMBER level using the FEP method 20

Uncertainty Analysis of Free Energy Barrier Predictions 22

Key distances (in Å) for the states located along the reaction progress. 33

Time evolution of interatomic distance between Oγ-Ser174 and C1 atom of the substrate in the product of the hydrolysis step 35

Occupancy analysis of water molecules inside the active site of the enzyme during the deacylation step 36

Experimental evaluation of the effect of the concentrations of Triton X100 over the esterase activity of the UMG – SP2. 47

References 48

Overlay of the structures of the unusual amidase of ClbL, and UMG-SP2, with the AlphaFold model of UMG-SP2

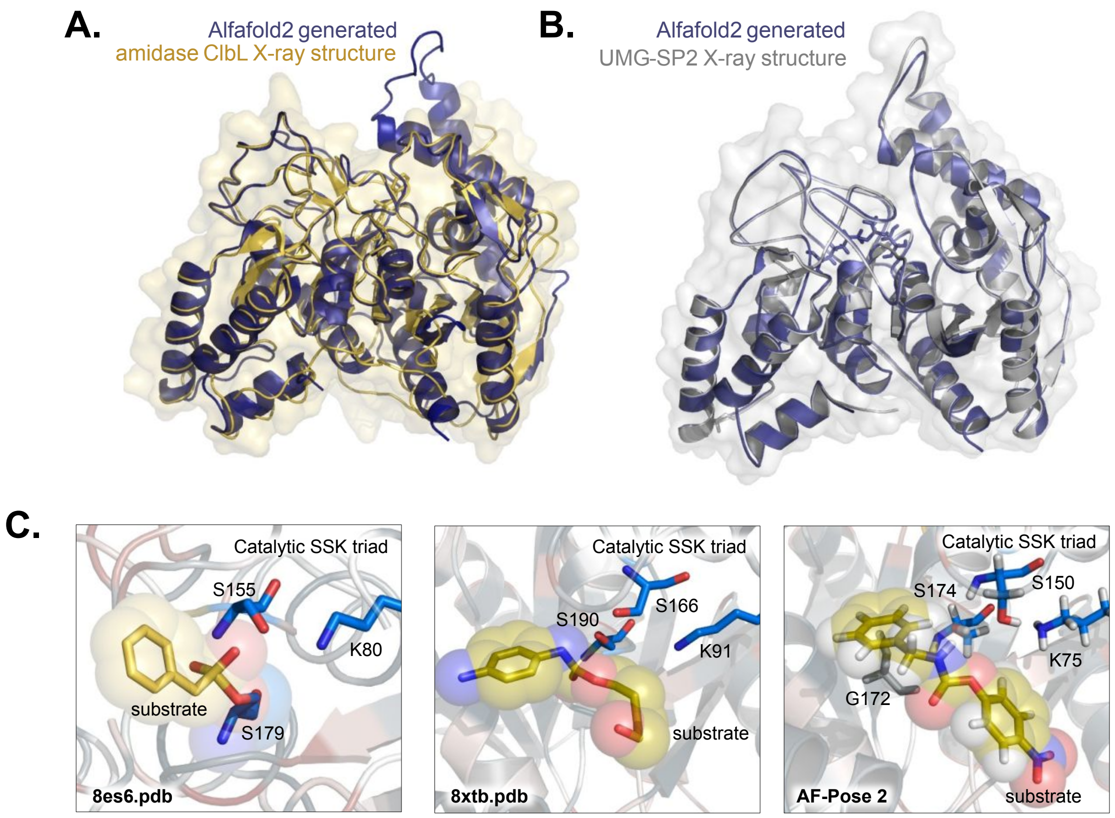

Figure S1. A. Overlay of the crystal structure of unusual amidase ClbL from the colibactin gene cluster with PDB code 8ES6 (in yellow) and the AlphaFold model of UMG-SP2 (in blue). **B.** Overlay of the AlphaFold model of UMG-SP2 (in blue) and the crystal structure of UMG-SP2 with PDB code 8WDW (in grey). **C.** Comparison of Pose 2 of pNC in the active site of the protein structure generated by AlphaFold and the pose of the original ligand 4-oxidanylbutyl ~{N}-(4-aminophenyl)carbamate employed during the crystallization of UMG-SP2 in 8xtb and the β-ketothioester substrate bound in 8es6.

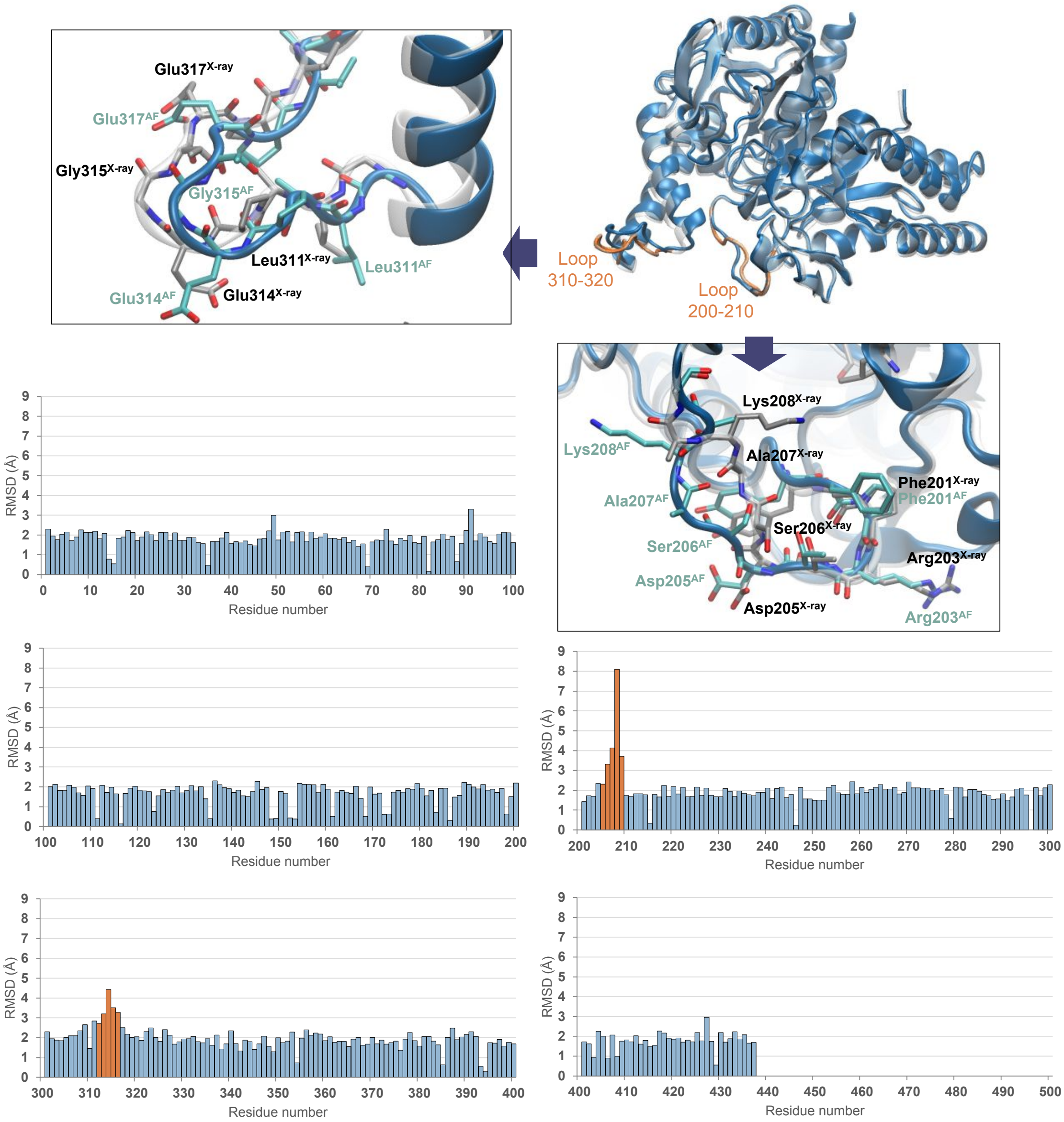

Figure S2. Overlay of the AlphaFold model of UMG-SP2 (in blue) and the crystal structure of UMG-SP2 with PDB code 8WDW (in grey), with amplified area of the two loops showing the largest RMSD computed by residue, as shown in the bottom panels, where those residues showing the highest RMSD values are highlighted I orange.

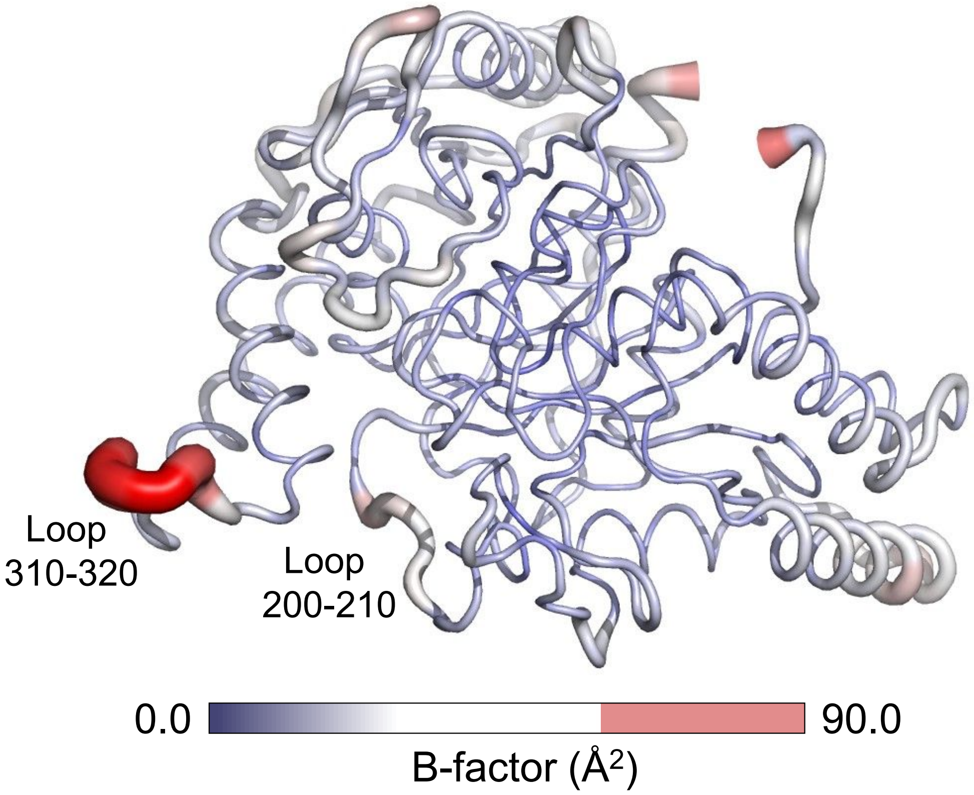

Figure S3. Crystallographic B-factor of the crystal structure of UMG-SP2 with PDB code 8WDW.

Sequence alignment of UMG-SP2 crystal structure and the model generated using AlfaFold2

```
UMG-SP2      GAMSELSAIETAAAIAGGSMTALEACDAAIARIEQRDGPINAVVVRDFDRARDAAKAADA      60
AF           ---SELSAIETAAAIAGGSMTALEACDAAIARIEQRDGPINAVVVRDFDRARDAAKAADA      57
                *********************************************************

UMG-SP2      EIAAAVRKPLLGVPMTIKESFDIAGLPTSWGFAEHADHIATADSLVVSRLKAAGAVFLGK      120
AF           EIAAAVRKPLLGVPMTIKESFDIAGLPTSWGFAEHADHIATADSLVVSRLKAAGAVFLGK      117
             ************************************************************

UMG-SP2      SNIPVGLADWQSVNPNYGRTNNPHDHSRSAGGSSGGAAAALAAGMVPLEYGSDIGGSIRV      180
AF           SNIPVGLADWQSVNPNYGRTNNPHDHSRSAGGSSGGAAAALAAGMVPLEYGSDIGGSIRV      177
             ************************************************************

UMG-SP2      PAHFCGVWGLKTTFDAVSLEGHYFPRTDSAKADLSVVGPMARTPADLALALDITSKVPLP      240
AF           PAHFCGVWGLKTTFDAVSLEGHYFPRTDSAKADLSVVGPMARTPADLALALDITSKVPLP      237
             ************************************************************

UMG-SP2      QSRIANLSGLRILLLTAHPETVADSATISAVERAAAACEASGATVATSSPDLPDLSALVA      300
AF           QSRIANLSGLRILLLTAHPETVADSATISAVERAAAACEASGATVATSSPDLPDLSALVA      297
             ************************************************************

UMG-SP2      DYTRMLLVVLARGLAPEGTEPVSLNAWYAMLDDQARMMRAFDRLFESFDAIFCPVLGTTA      360
AF           DYTRMLLVVLARGLAPEGTEPVSLNAWYAMLDDQARMMRAFDRLFESFDAIFCPVLGTTA      357
             ************************************************************

UMG-SP2      FAHSDEPDWAKRSLSIDGGIAPFAAQLGWISMATYGGMPALSMPLGADGNGLPINLQIIT      420
AF           FAHSDEPDWAKRSLSIDGGIAPFAAQLGWISMATYGGMPALSMPLGADGNGLPINLQIIT      417
             ************************************************************

UMG-SP2      RNWSDHDAIRIGALVAEALD-      440
AF           RNWSDHDAIRIGALVAEALDR      438
             ********************
```

Figure S4. Sequence alignment of UMG-SP2 crystal structure and model generated using AlfaFold2 obtained using Clustal ver.1.2.4.[1]

Sequence alignment of amidase ClbL and model generated using AlfaFold2

```
AF          ----SELSAIETAAAIAGGSMTALEACDAAIARIEQRDGPINAVVVRDFDRARDAAKAAD  56
ClbL        MSEQSYRSAGTLLAQLASGETTSVALVNHYFSRMAQFNKPLNAVVQQHYALALEAAARAD  60
                *  **    * :*.*. *::   :  ::*: * : *:**** :.:  * :**  **

AF          -AEIAAAVRKPLLGVPMTIKESFDIAGLPTSWGFAEHADHIATADSLVVSRLKAAGAVFL  115
ClbL        RERLEGRARGVLHGLPCTVKESFDVQGWLTTSGAHYLKDNRATQDAPSIARLRAAGAILM  120
              .: . .*  * *:* *:*****: *  *: *     *: ** *:  ::**:****:::

AF          GKSNIPVGLADWQSVNPNYGRTNNPHDHSRSAGGSSGGAAAALAAGMVPLEYGSDIGGSI  175
ClbL        GKTNVPMMTADWQTYNDLYGTTHNLWDRQRSPGGSSGGAAVAVAADFTPVEFGSDLFGSL  180
            **:*:*:  ****: *  ** *:*  *:.** ********.*:**.:.*:*:***: **:

AF          RVPAHFCGVWGLKTTFDAVSLEGHYFPRT--DSAKADLSVVGPMARTPADLALALDITSK  233
ClbL        RIPAHYTGVYAHRCSLGLMSVRGHVPGGGPQATDEPDLSTAGPMARSAADLRLMMRALST  240
            *:***: **:. : ::. :*:.**        : : ***..*****: *** * :   *.

AF          VPLPQSRIANLSGLR------ILLLTAHPETVADSATISA----V--ERAAAACEASGAT  281
ClbL        FWVEPPRIPDFSRYQAKANYRVCTWFSAPHHEIDQQIAQRFQSFIDKLRAQPGVEVDDAM  300
            . :   ** ::*  :      :    : *.   *.   .     :   **  . *...*

AF          VATSSPD-LPDLSA---------LVADYTRMLLVVLARGL---APEGTEPVSLNAWYA-M  327
ClbL        PADIDPDALFDIAVKLSGRLVSTALNGRQRLTAGLAALGFRLVGKLADVPEGITSYYQGM  360
             *  .** * *::.          : .  *:   : * *:   .  .  * .:.::*  *

AF          LDD----------QARMMRAFDRLFESFDAIFCPVLGTTAFAHSDEPDWAKRSLSIDGGI  377
ClbL        LKDSGEQRNTDKLRHEYSRVIETLFARYDVLLTPVSPVLAFAHMQQPV-RKRKLIVNGEP  419
            *.*          : .  *.:: **  :*.:: **  . **** ::*   **.* ::*

AF          APFAAQLGWISMATYGGMPALSMPLGADGNGLPINLQIITRNWSDHDAIRIGALVAEALD  437
ClbL        QDYNEHLFWNMLATVFGLPATVYPLAKTMDELPCGIQIISGHFHDDVTINFAEFCESISG  479
              :  :* *  :** *:**   **.   : ** .:***: :: *. :*.:. :  .  .

AF          R--------     438
ClbL        GFTVPEGYG     488
```

Figure S5. Sequence alignment (34% sequence homology) of the model generated using the AlfaFold model of UMG-SP2 and unusual amidase ClbL from the colibactin gene cluster obtained using Clustal ver.1.2.4.[1]

Results for pKa derived from PROPKA ver. 3.1 program

Table S1. List of the pKa of the titratable residues of metagenome-derived urethanase UMG-SP-2 (aa) as derived from PROPKA3.1 semiempirical program. Protonation states were assigned at pH 7 according to the obtained pKa values. HID: *Histidine* with hydrogen on the delta nitrogen; HIE: *Histidine* with hydrogen on the epsilon nitrogen, LYN: neutral lysine.

aa	pKa	model-pKa	aa	pKa	model-pKa	aa	pKa	model-pKa
ASP 24	3.16	3.80	GLU 7	3.62	4.50	LYN 75	6.40	10.50
ASP 34	4.06	3.80	GLU 21	3.89	4.50	LYS 108	10.11	10.50
ASP 44	4.00	3.80	GLU 31	4.59	4.50	LYS 117	8.30	10.50
ASP 46	3.87	3.80	GLU 58	3.76	4.50	LYN 188	7.02	10.50
ASP 50	3.00	3.80	GLU 76	6.47	4.50	LYS 208	11.28	10.50
ASP 56	3.57	3.80	GLU 91	3.75	4.50	LYS 233	10.61	10.50
ASP 79	5.18	3.80	GLU 166	7.77	4.50	LYS 368	10.60	10.50
ASP 94	2.45	3.80	GLU 197	4.72	4.50	ARG 29	11.23	12.50
ASP 100	3.02	3.80	GLU 257	3.89	4.50	ARG 33	12.99	12.50
ASP 126	4.49	3.80	GLU 269	4.86	4.50	ARG 43	12.92	12.50
ASP 142	2.01	3.80	GLU 276	4.70	4.50	ARG 47	13.86	12.50
ASP 170	1.98	3.80	GLU 314	4.53	4.50	ARG 49	13.72	12.50
ASP 192	2.26	3.80	GLU 317	4.61	4.50	ARG 64	12.23	12.50
ASP 205	3.99	3.80	GLU 343	4.70	4.50	ARG 106	11.85	12.50
ASP 210	4.83	3.80	GLU 363	4.25	4.50	ARG 136	12.53	12.50
ASP 223	4.69	3.80	GLU 434	4.51	4.50	ARG 145	12.83	12.50
ASP 229	3.76	3.80	HID 92	6.02	6.50	ARG 176	13.11	12.50
ASP 261	4.73	3.80	HID 95	5.55	6.50	ARG 203	13.32	12.50
ASP 288	4.03	3.80	HIE 141	6.04	6.50	ARG 219	10.80	12.50
ASP 291	2.95	3.80	HID 143	6.08	6.50	ARG 240	12.46	12.50
ASP 298	4.38	3.80	HID 180	4.01	6.50	ARG 248	13.85	12.50
ASP 329	3.88	3.80	HID 199	2.44	6.50	ARG 270	12.03	12.50
ASP 330	2.35	3.80	HID 255	4.55	6.50	ARG 301	13.72	12.50
ASP 339	4.43	3.80	HID 360	4.47	6.50	ARG 309	12.18	12.50
ASP 346	2.85	3.80	HIE 423	6.38	6.50	ARG 333	13.57	12.50
ASP 362	3.19	3.80	TYR 134	12.35	10.00	ARG 336	12.08	12.50
ASP 365	3.16	3.80	TYR 167	13.28	10.00	ARG 340	12.38	12.50
ASP 374	3.21	3.80	TYR 200	11.22	10.00	ARG 369	13.27	12.50
ASP 405	2.75	3.80	TYR 299	13.49	10.00	ARG 418	12.40	12.50
ASP 422	5.36	3.80	TYR 325	10.15	10.00	ARG 427	12.20	12.50
ASP 424	4.50	3.80	TYR 392	12.62	10.00	ARG 438	12.52	12.50
ASP 437	4.08	3.80	LYS 53	11.32	10.50			
GLU 2	3.18	4.50	LYS 65	11.22	10.50			

Results for pKa based on constant-pH neMD/MC simulations

All simulations attempted protonation moves every 10 ps over 50 ns with switch times of 20 ps (i.e., 5000 neMD/MC cycles). Constant-pH MD assays of the titration curves were performed on 73 pH values between 0.2 and 14.8 at intervals of 0.2 units and repeated three times for each substrate orientation: to promote the amidase activity (amidase) or the esterase activity (esterase).

Values of pKa computed for aspartic acid residues

Table S2. Values of pKa computed for aspartic acid residues using pH-constant *ne*MD/MC simulations for amidase (on the left) and esterase (on the right).

Residue	Amidase	Esterase	Residue	Amidase	Esterase
	pKa			pKa	
Asp24	3.277 ± 0.023	3.191 ± 0.047	Asp229	3.685 ± 0.032	3.776 ± 0.034
Asp34	4.137 ± 0.014	4.116 ± 0.036	Asp261	4.825 ± 0.020	4.800 ± 0.027
Asp44	4.069 ± 0.016	3.996 ± 0.042	Asp288	3.968 ± 0.018	3.894 ± 0.031
Asp46	3.899 ± 0.025	3.793 ± 0.045	Asp291	2.832 ± 0.038	2.798 ± 0.042
Asp50	2.981 ± 0.031	2.939 ± 0.016	Asp298	4.320 ± 0.013	4.419 ± 0.023
Asp56	3.674 ± 0.024	3.564 ± 0.020	Asp329	3.869 ± 0.034	3.810 ± 0.040
Asp79	5.197 ± 0.009	5.286 ± 0.010	Asp330	2.400 ± 0.016	2.295 ± 0.020
Asp94	2.454 ± 0.033	2.396 ± 0.032	Asp339	4.467 ± 0.033	4.481 ± 0.027
Asp100	2.961 ± 0.025	2.987 ± 0.020	Asp346	2.853 ± 0.024	2.774 ± 0.023
Asp126	4.431 ± 0.024	4.503 ± 0.034	Asp362	3.127 ± 0.019	3.060 ± 0.046
Asp142	1.996 ± 0.020	2.087 ± 0.022	Asp365	3.183 ± 0.029	3.222 ± 0.020
Asp170	1.992 ± 0.014	1.998 ± 0.023	Asp374	3.220 ± 0.027	3.241 ± 0.021
Asp192	2.227 ± 0.018	2.263 ± 0.024	Asp405	2.786 ± 0.026	2.663 ± 0.022
Asp205	3.967 ± 0.017	3.871 ± 0.024	Asp422	5.394 ± 0.016	5.281 ± 0.010
Asp210	4.853 ± 0.015	4.773 ± 0.016	Asp424	4.589 ± 0.025	4.518 ± 0.040
Asp223	4.687 ± 0.020	4.629 ± 0.026	Asp437	4.003 ± 0.036	4.068 ± 0.031

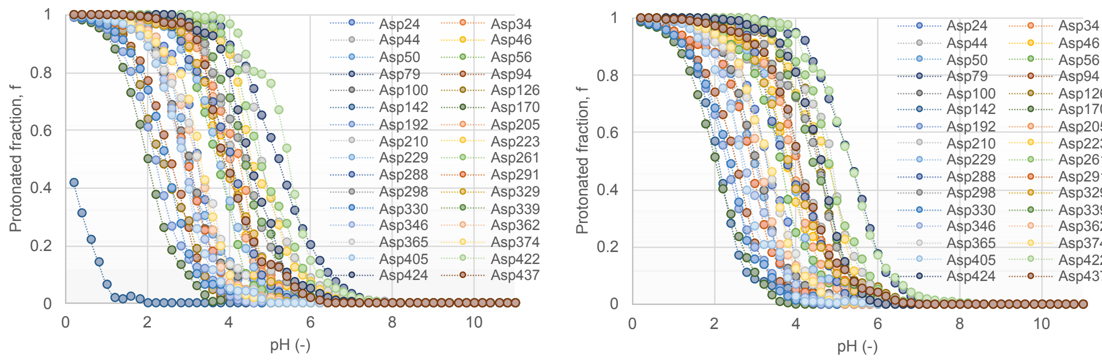

Figure S6. Titration curves computed for aspartic acid residues using pH-constant *ne*MD/MC simulations for amidase (on the left) and esterase (on the right).

Values of pKa computed for glutamic acid residues

Table S3. Values of pKa computed for glutamic acid residues using pH-constant *ne*MD/MC simulations for amidase (on the left) and esterase (on the right).

Residue	Amidase	Esterase	Residue	Amidase	Esterase
	pKa			pKa	
Glu2	3.588 ± 0.042	2.837 ± 0.055	Glu257	3.843 ± 0.026	3.866 ± 0.036
Glu7	4.201 ± 0.041	4.070 ± 0.056	Glu269	4.822 ± 0.019	4.770 ± 0.013
Glu21	3.904 ± 0.043	4.006 ± 0.032	Glu276	4.635 ± 0.010	4.784 ± 0.015
Glu31	4.778 ± 0.021	4.427 ± 0.010	Glu314	4.506 ± 0.017	4.493 ± 0.026
Glu58	3.730 ± 0.013	3.808 ± 0.023	Glu317	4.655 ± 0.012	4.586 ± 0.011
Glu76	6.274 ± 0.016	6.422 ± 0.009	Glu343	4.801 ± 0.015	4.765 ± 0.014
Glu91	3.672 ± 0.009	3.610 ± 0.025	Glu363	4.268 ± 0.029	4.318 ± 0.016
Glu166	7.786 ± 0.006	7.697 ± 0.003	Glu434	4.576 ± 0.009	4.533 ± 0.011
Glu197	4.710 ± 0.008	4.746 ± 0.018			

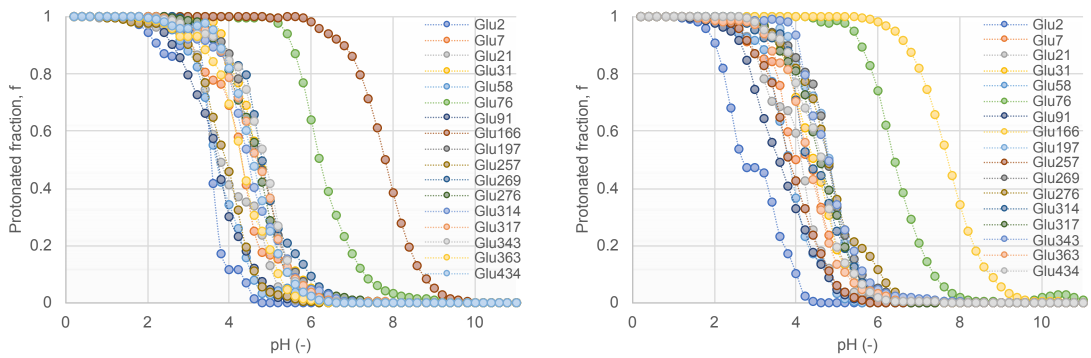

Figure S7. Titration curves computed for glutamic acid residues using pH-constant *ne*MD/MC simulations for amidase (on the left) and esterase (on the right).

<u>Values of pKa computed for histidine residues</u>

Table S4. Values of pKa computed for histidine residues using pH-constant *ne*MD/MC simulations for amidase (on the left) and esterase (on the right).

Residue	Amidase	Esterase	Residue	Amidase	Esterase
	pKa			pKa	
His92	5.726 ± 0.010	5.714 ± 0.010	His199	1.998 ± 0.015	2.138 ± 0.010
His95	5.226 ± 0.011	5.269 ± 0.008	His255	4.231 ± 0.028	4.219 ± 0.027
His141	5.775 ± 0.010	5.686 ± 0.013	His360	4.092 ± 0.028	4.179 ± 0.030
His143	5.802 ± 0.018	5.817 ± 0.003	His423	6.129 ± 0.010	6.117 ± 0.018
His180	3.507 ± 0.032	3.617 ± 0.026			

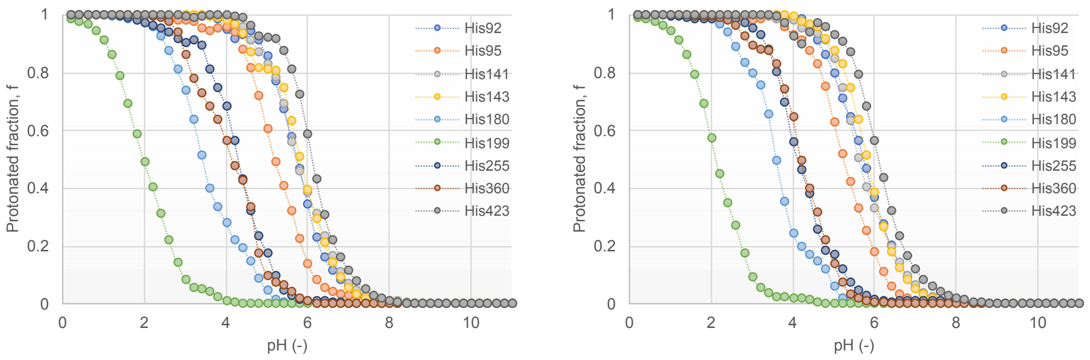

Figure S8. Titration curves computed for histidine residues using pH-constant *ne*MD/MC simulations for amidase (on the left) and esterase (on the right).

Values of pKa computed for cysteine residues

Table S5. Values of pKa computed for cysteine residues using pH-constant *ne*MD/MC simulations for amidase (on the left) and esterase (on the right).

Residue	Amidase	Esterase	Residue	Amidase	Esterase
	pKa			pKa	
Cys23	10.324 ± 0.002	10.255 ± 0.003	Cys275	9.449 ± 0.003	9.481 ± 0.003
Cys182	9.478 ± 0.003	9.500 ± 0.001	Cys350	9.561 ± 0.006	9.459 ±0.005

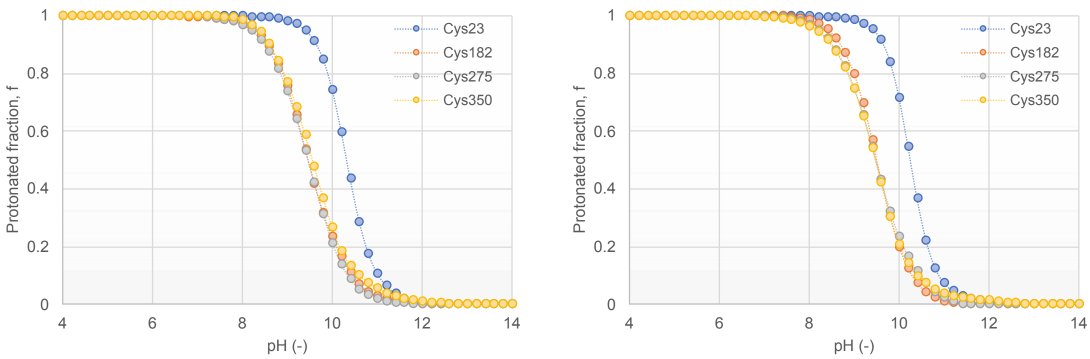

Figure S9. Titration curves computed for cysteine residues using pH-constant *ne*MD/MC simulations for amidase (on the left) and esterase (on the right).

<u>Values of pKa computed for lysine residues</u>

Table S6. Values of pKa computed for lysine residues using pH-constant *ne*MD/MC simulations for amidase (on the left) and esterase (on the right).

Residue	Amidase	Esterase	Residue	Amidase	Esterase
	pKa			pKa	
Lys53	11.522 ± 0.002	11.492 ± 0.002	**Lys188**	**7.076 ± 0.007**	**7.020 ± 0.003**
Lys65	11.351 ± 0.002	11.344 ± 0.007	Lys208	11.293 ± 0.006	11.325 ± 0.002
**Lys75**	**6.390 ± 0.027**	**6.247 ± 0.018**	Lys233	10.584 ± 0.004	10.623 ± 0.003
Lys108	9.991 ± 0.006	10.061 ± 0.006	Lys368	10.609 ± 0.004	10.581 ± 0.002
Lys117	8.387 ± 0.005	8.596 ± 0.009			

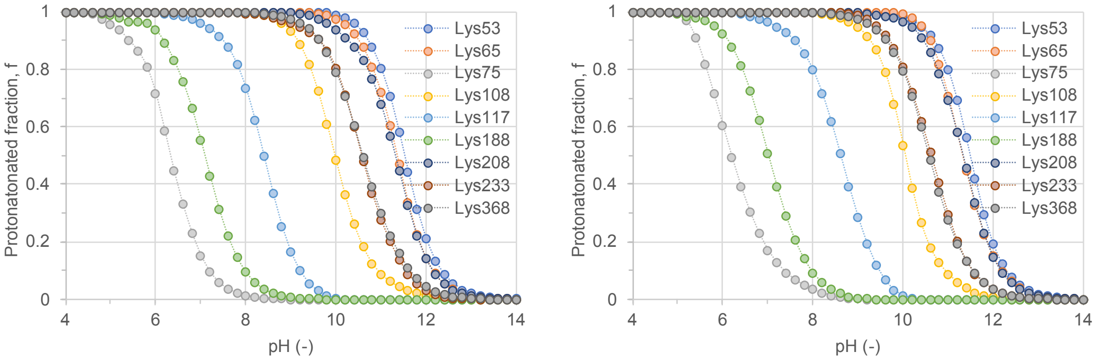

Figure S10. Titration curves computed for lysine residues using pH-constant *ne*MD/MC simulations for amidase (on the left) and esterase (on the right).

Position of Lys188 concerning the catalytic triad in the active site

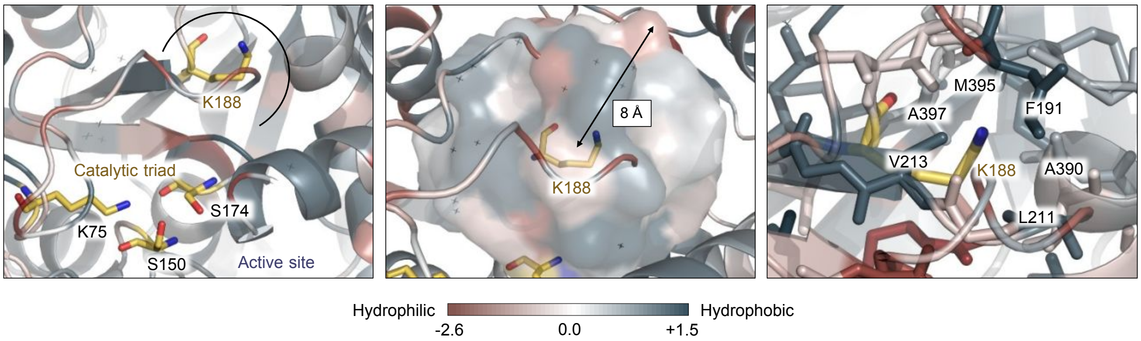

Figure S11. Position of Lys188 concerning the catalytic triad in the active site (on the left), surface within 8 Å from Lys188 with a hydrophobicity map generated based on the Eisenberg hydrophobicity scale[2] (in the center), and the detailed surrounding of Lys188 indicates high hydrophobic residues in the proximity of this residue (in the right).

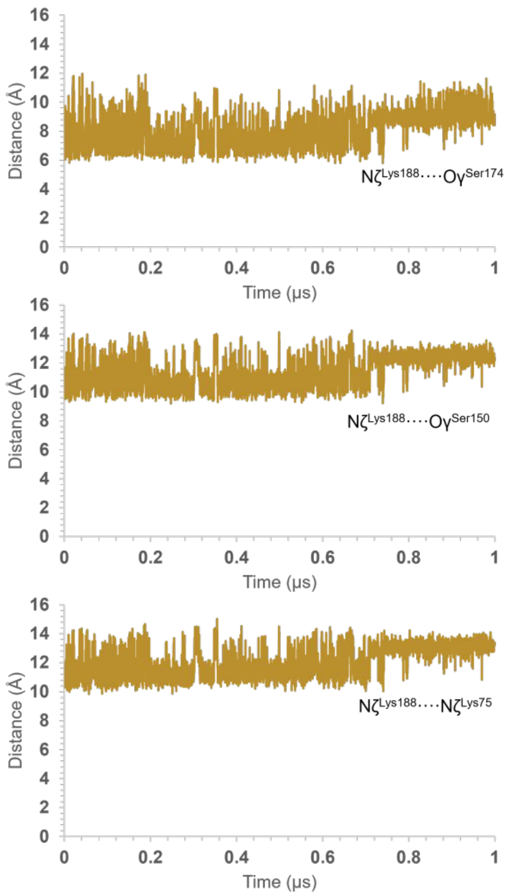

Figure S12. Evolution of the distance between Lys188 and catalytic Ser174 (top panel), Ser150 (middle panel) and Lys75 (bottom panel), established during 1 μs of classical MD simulations for the substrate bound in pose 2.

FF parameters for the 4-nitrophenyl benzylcarbamate substrate

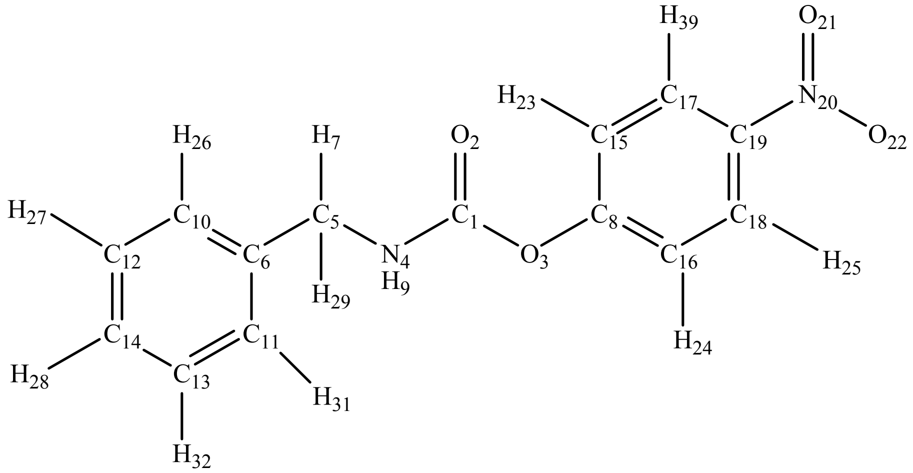

Figure S13. Molecule structure and numbering scheme for the substrate 4-nitrophenyl benzylcarbamate, pNC.

Table S7. Atom types, charges and parameters obtained for the substrate generated on the **E:S** reactant complex using the antechamber package included in AmberTools.

<table>
<tr><th>Atom name</th><th>Atom type</th><th>Charge</th><th>Parameters</th></tr>
<tr><td>O21</td><td>o</td><td>-0.209500</td><td rowspan="29"><u>NONBON</u>
o 1.7107 0.1463
no 1.8886 0.0858
ca 1.8606 0.0988
ha 1.4735 0.0161
os 1.7713 0.0726
c 1.8606 0.0988
ns 1.8352 0.1174
hn 0.6210 0.0100
c3 1.9069 0.1078
h1 1.3593 0.0208

<u>BOND</u>
no-o 361.74 1.231
ca-no 250.20 1.470
ca-ca 354.25 1.399
ca-ha 360.69 1.086
ca-os 343.34 1.369
c -os 351.31 1.362
c -o 590.60 1.219
c -ns 336.07 1.380
hn-ns 473.59 1.013
c3-ns 261.66 1.456
c3-h1 344.22 1.097
c3-ca 243.91 1.515

<u>ANGLE</u>
o -no-o 131.020 125.050
ca-no-o 99.080 117.760
ca-ca-no 66.120 119.000
ca-ca-ca 63.670 120.020
ca-ca-ha 44.900 119.880
ca-ca-os 79.000 119.110
c -os-ca 89.360 121.290
o -c -os 101.260 123.200
ns-c -os 88.540 108.880</td></tr>
<tr><td>N20</td><td>no</td><td>0.315200</td></tr>
<tr><td>O22</td><td>o</td><td>-0.209500</td></tr>
<tr><td>C19</td><td>ca</td><td>-0.191200</td></tr>
<tr><td>C18</td><td>ca</td><td>-0.053500</td></tr>
<tr><td>C16</td><td>ca</td><td>-0.148500</td></tr>
<tr><td>H24</td><td>ha</td><td>0.169500</td></tr>
<tr><td>H25</td><td>ha</td><td>0.174500</td></tr>
<tr><td>C17</td><td>ca</td><td>-0.053500</td></tr>
<tr><td>H30</td><td>ha</td><td>0.174500</td></tr>
<tr><td>C15</td><td>ca</td><td>-0.148500</td></tr>
<tr><td>H23</td><td>ha</td><td>0.169500</td></tr>
<tr><td>C8</td><td>ca</td><td>0.167100</td></tr>
<tr><td>O3</td><td>os</td><td>-0.376200</td></tr>
<tr><td>C1</td><td>c</td><td>0.734100</td></tr>
<tr><td>O2</td><td>o</td><td>-0.556000</td></tr>
<tr><td>N4</td><td>ns</td><td>-0.497900</td></tr>
<tr><td>H9</td><td>hn</td><td>0.316500</td></tr>
<tr><td>C5</td><td>c3</td><td>0.120300</td></tr>
<tr><td>H7</td><td>h1</td><td>0.080700</td></tr>
<tr><td>H29</td><td>h1</td><td>0.080700</td></tr>
<tr><td>C6</td><td>ca</td><td>-0.138300</td></tr>
<tr><td>C10</td><td>ca</td><td>-0.113500</td></tr>
<tr><td>H26</td><td>ha</td><td>0.136000</td></tr>
<tr><td>C12</td><td>ca</td><td>-0.129000</td></tr>
<tr><td>H27</td><td>ha</td><td>0.137000</td></tr>
<tr><td>C14</td><td>ca</td><td>-0.117000</td></tr>
<tr><td>H28</td><td>ha</td><td>0.136000</td></tr>
<tr><td>C13</td><td>ca</td><td>-0.129000</td></tr>
</table>

H32	ha	0.137000	c -ns-hn 57.370 117.260<br>c -ns-c3 76.900 121.960<br>ns-c -o 87.210 123.050<br>h1-c3-ns 48.390 108.490
C11	ca	-0.113500	
H31	ha	0.136000	

Parameters

ANGLE

ca-c3-ns 65.840 112.020
c3-ns-hn 54.620 117.420
c3-ca-ca 60.740 120.830
h1-c3-h1 35.640 108.550
ca-c3-h1 43.810 109.600

IMPROPER

ca-o -no-o	1.1	180.0	2.0
ca-ca-ca-no	1.1	180.0	2.0
ca-ca-ca-ha	1.1	180.0	2.0
ca-ca-ca-os	1.1	180.0	2.0
ns-o -c -os	1.1	180.0	2.0
c -c3-ns-hn	1.1	180.0	2.0
c3-ca-ca-ca	1.1	180.0	2.0

DIHEDRALS

ca-ca-no-o 1 0.720 180.000
2.000
ca-ca-ca-no 4 14.500 180.000
2.000
ha-ca-ca-no 4 14.500 180.000
2.000
ca-ca-ca-ha 4 14.500 180.000
2.000
ca-ca-ca-ca 4 14.500 180.000
2.000

DIHEDRALS

ca-ca-ca-os 4 14.500 180.000
2.000
ca-ca-os-c 1 0.980 180.000
2.000
ha-ca-ca-os 4 14.500 180.000
2.000
ha-ca-ca-ha 4 14.500 180.000
2.000
o -c -os-ca 1 2.150 180.000 -
2.000
o -c -os-ca 1 0.080 0.000 -
3.000
o -c -os-ca 1 1.730 0.000
1.000
ns-c -os-ca 2 5.400 180.000
2.000
os-c -ns-hn 4 10.000 180.000
2.000
os-c -ns-c3 4 10.000 180.000
2.000
h1-c3-ns-c 1 0.000 0.000
1.000
ca-c3-ns-c 6 0.000 0.000
2.000
o -c -ns-hn 1 2.500 180.000 -
2.000
o -c -ns-hn 1 2.000 0.000
1.000
o -c -ns-c3 4 10.000 180.000
2.000
ns-c3-ca-ca 6 0.000 0.000
2.000
h1-c3-ns-hn 6 0.000 0.000
2.000
ca-c3-ns-hn 6 0.000 0.000
2.000
c3-ca-ca-ha 4 14.500 180.000
2.000
c3-ca-ca-ca 4 14.500 180.000
2.000
h1-c3-ca-ca 6 0.000 0.000
2.000

Results of MD simulations

RMSD for heavy atoms of protein backbone and substrate

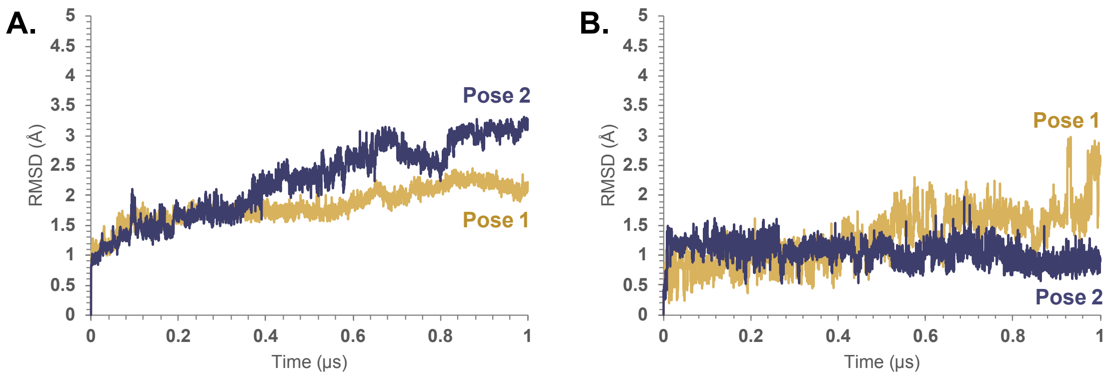

Figure S14. RMSD for heavy atoms of **A.** protein backbone and **B.** substrate along 1 μs classical NPT MD simulations.

Evolution of key distances in Pose 1

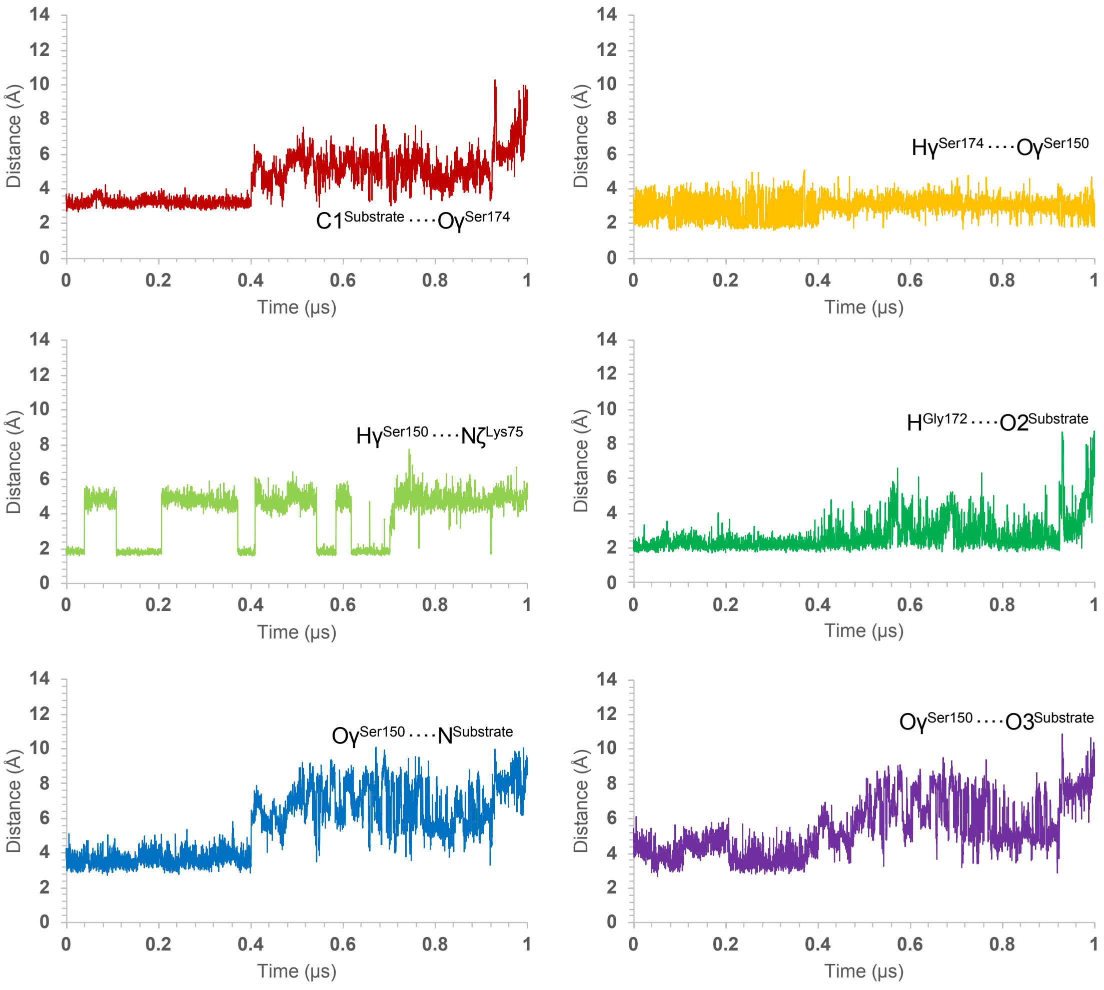

Figure S15. Evolution of key distances established during 1 μs classical NPT MD simulations in Pose 1 of the substrate.

Evolution of key distances in Pose 2

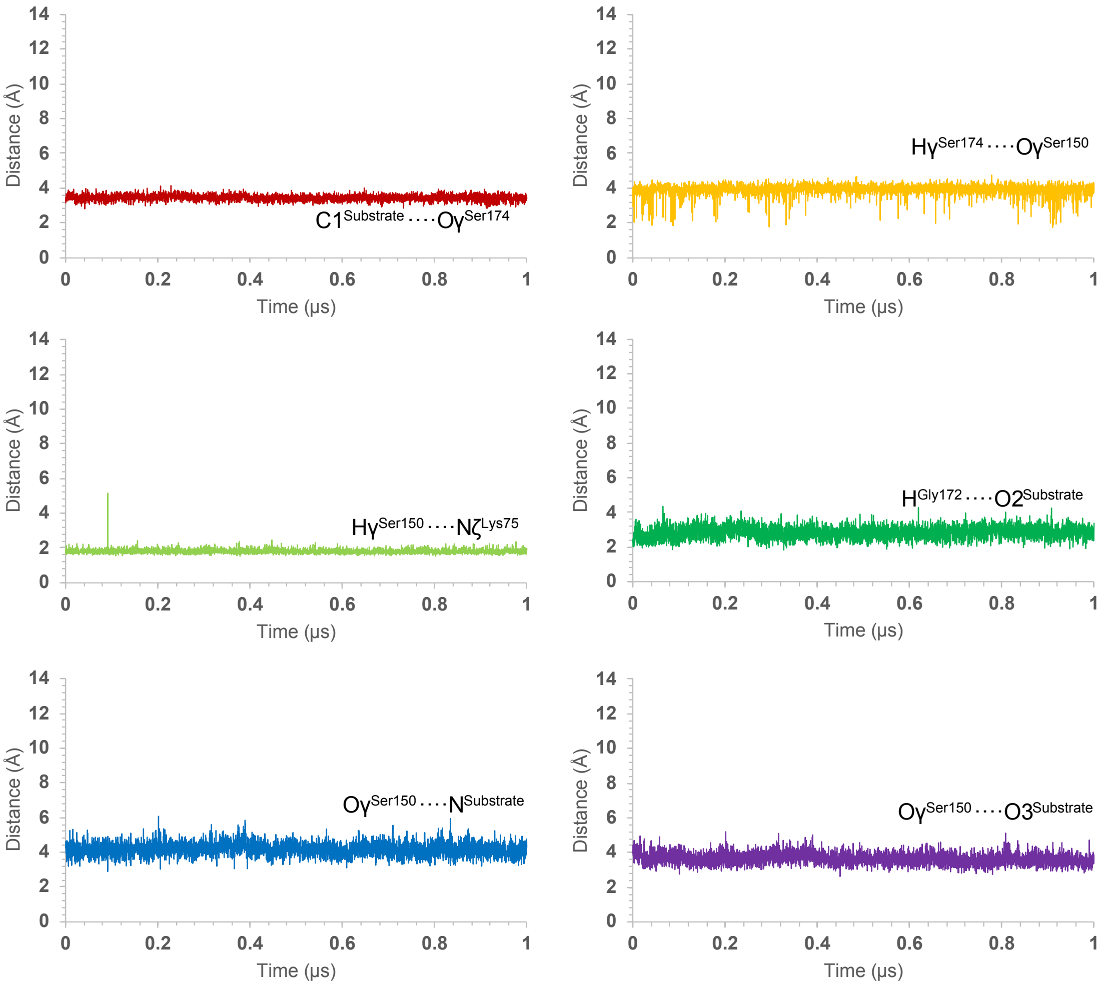

Figure S16. Evolution of key distances established during 1 μs classical NPT MD simulations in Pose 2 of the substrate.

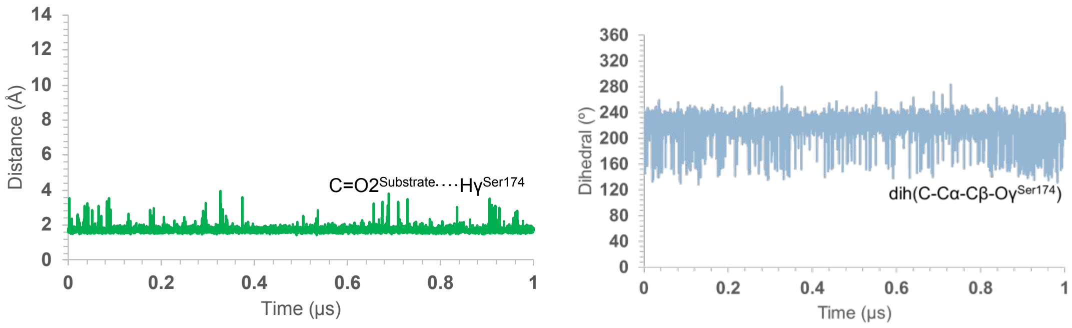

Figure S17. Evolution of the distance between Ser174 and the oxygen of the carbonyl group of the substrate (left panel) and the dihedral angle describing the orientation of Ser174 (right panel) from ES inactive conformation established during 1 μs of classical MD simulations for the substrate bound in pose 2.

Schematic representation of the active site

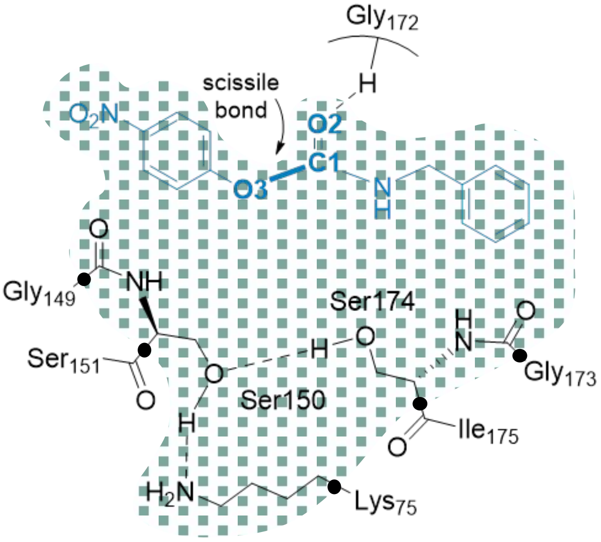

Figure S18. Schematic representation of the active site metagenome-derived urethanase UMG-SP-2 in complex with the pNC substrate in the non-covalent reactant complex, E·S. Light blue region contains atoms treated quantum mechanically. Dashed lines indicate hydrogen bond interactions. Quantum link atoms are represented as black dots. Representation of the Pose 2 that promotes the esterase activity of UMG-SP-2.

Free energy profiles computed at the M06-2X/AMBER level using the FEP method

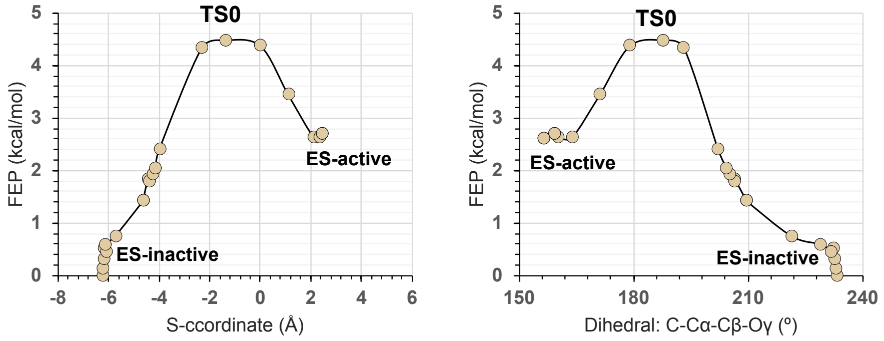

Figure S19. Free energy profile computed at M06-2X/AMBER for Ser174 rotation from inactive to active form as a function of internal s-coorditate and dihedral angle formed between C-Cα-Cβ-Oγ.

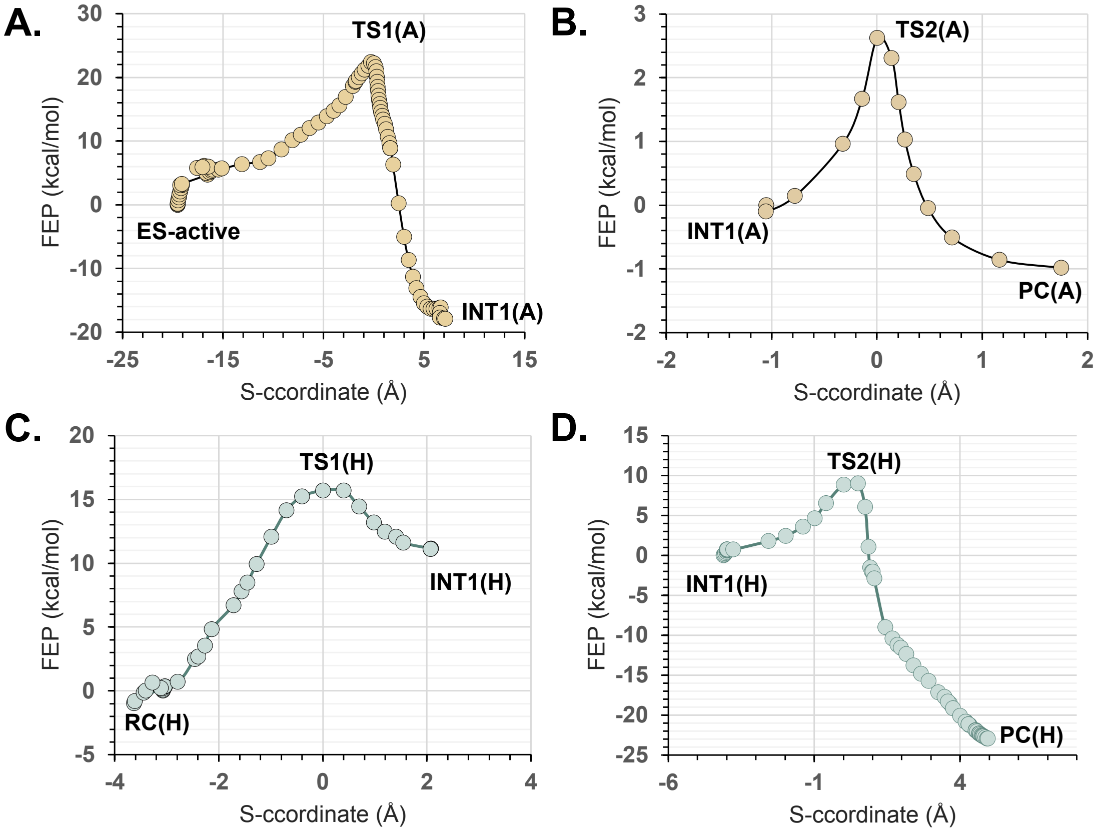

Figure S20. Free energy profiles computed at the M06-2X/AMBER level using the FEP method for individual reaction steps of the hydrolysis of the carbamate bond of pNC catalyzed by UMG-SP2. **A.** Acylation step involving activation of the Ser174 residue via proton transfer through Ser150 to Lys75, and nucleophilic attack of Ser174 on carbony pNC carbon. **B.** Proton transfer from Lys75 via Ser150 to the oxygen of pNC, leading to ester bond cleavage and departure of the leaving group. **C.** Activation of a water molecule by proton transfer through Ser150 to Lys75, followed by a nucleophilic attack of the water oxygen on the carbonyl carbon of pNC. **D.** Reactivation of the active site via cleavage of the Ser174–pNC linkage and protonation of the Ser174 residue.

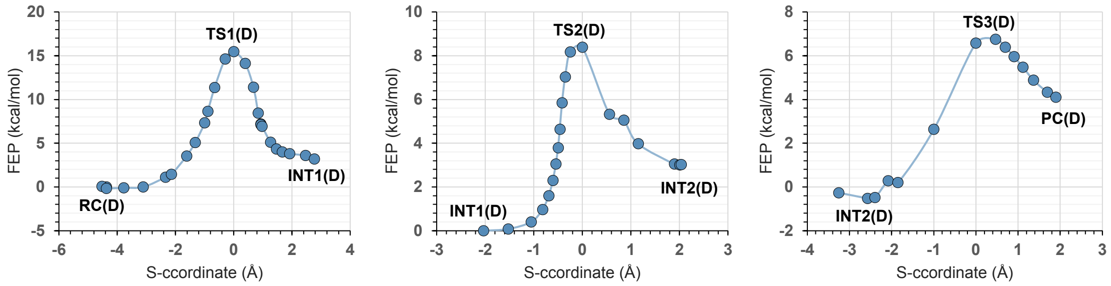

Figure S21. Free energy profiles computed at the M06-2X/AMBER level using the FEP method for individual reaction steps of the decarboxylation step of the product of the hydrolysis step in the pNC degradation catalyzed by UMG-SP2.

Table S8. Free energies for acylation step of pNC catalyzed by UMG-SP2 computed at M06-2X/AMBER level using FEP method. Values of energies are given in kcal/mol.

	ΔG(FEP)	ΔΔG	ZPEcorr.	ΔG+ZPE	ΔΔ(G+ZPE)
ES-inactive	0.00	0.00	390.0	389.97	0.0
TS0	4.48	4.48	388.9	393.35	3.4
ES-active	2.60	2.60	389.2	391.80	1.8
TS1	22.26	24.86	386.3	411.14	21.2
INT1	-17.91	-15.30	389.8	374.45	-15.5
TS2	2.62	-12.68	386.0	373.36	-16.6
INT2	-0.98	-16.29	389.6	373.32	-16.7

Table S9. Free energies for deacylation step of pNC catalyzed by UMG-SP2 computed at M06-2X/AMBER level using FEP method. Values of energies are given in kcal/mol.

	ΔG(FEP)	ΔΔG	ZPEcorr.	ΔG+ZPE	ΔΔ(G+ZPE)
RC	0.00	0	337.4	337.44	0.0
TS1(H)	15.69	15.69	337.7	353.37	15.9
INT1(H)	11.11	11.11	339.6	350.68	13.2
TS2(H)	8.97	20.08	335.6	355.66	18.2
INT1	-22.95	-11.83	338.1	326.31	-11.1

Table S10. Free energies for decarboxylation step of pNCcatalyzed by UMG-SP2 computed at M06-2X/AMBER level using FEP method. Values of energies are given in kcal/mol.

	ΔG(FEP)	ΔΔG	ZPEcorr.	ΔG+ZPE	ΔΔ(G+ZPE)
RC(D)	0.00	0.00	337.9	337.86	0.0
TS1(D)	15.47	15.47	334.5	350.00	12.1
INT1(D)	3.17	3.17	338.7	341.83	4.0
TS2(D)	8.39	11.55	334.7	346.21	8.3
INT2(D)	3.02	6.18	337.7	343.86	6.0
TS3(D)	6.75	12.93	336.9	349.85	12.0
PC(D)	4.11	10.29	336.7	346.97	9.1

Uncertainty Analysis of Free Energy Barrier Predictions

The free energy change between two consecutive points in the traced QM/MM IRC, i and i+1, as shown in equations 1 and 2 of the main text, can be expressed as:

$$\Delta G_{i\rightarrow i+1} = -RTln\left(\frac{1}{N}\sum_{n=1}^{N} e^{-\frac{\Delta E_{i\rightarrow i+1}^{n}}{RT}}\right) = -RTln\langle e^{-Tot/RT}\rangle_{i} \qquad \text{(S1)}$$

In equation S1, $\Delta E_{i\rightarrow i+1}$ is the difference between electrostatic and Lennard-Jones energies (ΔE_{elec}+ ΔE_{LJ}) in i and i+1 step of the IRC. Then, in order to compute the error (standard error) of $\Delta G_{i\rightarrow i+1}$, the error propagation along the FEP paths of every chemical step can be computed according to equation S2:

$$\sigma_{\Delta G_{i\rightarrow i+1}} = \frac{RT}{\langle e^{-Tot/RT}\rangle} \cdot \sigma_{e^{-Tot/RT}}/\sqrt{N} \qquad \text{(S2)}$$

This assumes the multivariable error propagation for the logarithmic function. Therefore, since the free energy barrier of every chemical step, $\Delta G^{\ddagger}$, is determined according to $\Delta G^{\ddagger} = \sum_{i=1}^{n} \Delta G_{i\rightarrow i+1}$, the total uncertainty can be calculated from:

$$\sigma^{\ddagger} = \sqrt{\sum_{i=1}^{n} \sigma_i^2} \qquad \text{(S3)}$$

The results for every chemical step are listed in Tables S11-As shown in the provided Tables, the error of our prediction ranges between **0.06 and 0.97** kcal·mol^{-1}.

Table S11. Determined errors of computed free energies ($\Delta G^{\ddagger}$), for every step ($\sigma_{\Delta G_{i \to i+1}}$) and total free energy barrier ($\sigma^{\ddagger}$) explored using FEP method for Ser174 rotation from inactive to active form ("ES inactive" to "ES active"). Results were obtained at M06-2X/AMBER level of theory and energy values are provided in kcal/mol.

s-coor (Å)	$\Delta G_{i \to i+1}$	$\sigma(\Delta G_{i \to i+1})$	FEP	$\sigma^2(\Delta G_{i \to i+1})$	$\sigma^{\ddagger}$
-6.240	0.0000	0.000	0	0	**0.21**
-6.223	0.1289	0.005	0.13	2.20E-05	
-6.202	0.1917	0.007	0.32	4.84E-05	
-6.192	0.1956	0.006	0.52	4.04E-05	
-6.116	-0.0561	0.020	0.46	4.08E-04	
-6.140	0.1382	0.048	0.60	2.31E-03	
-5.739	0.1554	0.053	0.75	2.86E-03	
-4.641	0.6652	0.081	1.42	6.53E-03	
-4.449	0.4198	0.053	1.84	2.80E-03	
-4.394	-0.0526	0.029	1.79	8.50E-04	
-4.250	0.1348	0.013	1.92	1.76E-04	
-4.157	0.1289	0.011	2.05	1.14E-04	
-3.998	0.3546	0.041	2.40	1.67E-03	
-2.318	1.9308	0.156	4.34	2.42E-02	
-1.377	0.1475	0.039	4.48	1.52E-03	
0.000	-0.1090	0.075	4.37	5.65E-03	
1.092	-0.9257	0.040	3.45	1.63E-03	
2.096	-0.8150	0.036	2.63	1.29E-03	
2.350	0.0081	0.022	2.64	4.95E-04	
2.414	0.0519	0.007	2.69	4.39E-05	
2.414	0.0002	0.000	2.69	2.68E-09	
2.698	-0.0891	0.020	2.60	4.07E-04	

Table S12. Determined errors of computed free energies ($\Delta G^{\ddagger}$), for every step ($\sigma_{\Delta G_{i \to i+1}}$) and total free energy barrier ($\sigma^{\ddagger}$) explored using FEP method for <u>first step of the acylation</u> involving activation of the Ser174 residue via proton transfer through Ser150 to Lys75, and nucleophilic attack of Ser174 on carbony pNC carbon form ("ES active" to "INT1(A)"). Results were obtained at M06-2X/AMBER level of theory and energy values are provided in kcal/mol.

s-coor (Å)	$\Delta G_{i \to i+1}$	$\sigma(\Delta G_{i \to i+1})$	FEP	$\sigma^2(\Delta G_{i \to i+1})$	$\sigma^{\ddagger}$
-19.53	0.0000	0.000	0.00	0	**0.47**
-19.53	0.1213	0.008	0.12	5.77E-05	
-19.52	0.0877	0.004	0.21	1.94E-05	
-19.47	0.5156	0.021	0.72	4.27E-04	
-19.41	0.4596	0.017	1.18	2.88E-04	
-19.34	0.4380	0.016	1.62	2.67E-04	
-19.26	0.4943	0.017	2.12	2.76E-04	
-19.19	0.5417	0.013	2.66	1.79E-04	
-19.08	0.4298	0.015	3.09	2.17E-04	
-19.26	0.0076	0.009	3.10	7.68E-05	
-19.08	0.1816	0.015	3.28	2.27E-04	
-16.53	1.4586	0.196	4.74	3.85E-02	
-16.68	0.2689	0.012	5.00	1.39E-04	
-16.34	0.1665	0.040	5.17	1.62E-03	
-16.13	0.1701	0.012	5.34	1.53E-04	
-16.03	0.1959	0.041	5.54	1.65E-03	
-17.58	0.2464	0.088	5.78	7.73E-03	
-15.48	-0.2404	0.334	5.54	1.12E-01	
-16.86	0.5661	0.087	6.11	7.60E-03	
-16.44	-0.0983	0.023	6.01	5.36E-04	
-17.03	-0.0672	0.053	5.94	2.84E-03	
-15.14	-0.1957	0.091	5.75	8.34E-03	
-13.13	0.6174	0.076	6.37	5.80E-03	
-11.30	0.3387	0.099	6.70	9.76E-03	
-10.49	0.6336	0.040	7.34	1.57E-03	
-9.18	1.3780	0.058	8.72	3.37E-03	
-8.10	1.4371	0.050	10.15	2.53E-03	
-7.27	0.8795	0.038	11.03	1.44E-03	
-6.41	1.0193	0.039	12.05	1.49E-03	
-5.52	0.8841	0.035	12.94	1.20E-03	
-4.68	0.9728	0.038	13.91	1.47E-03	
-4.02	0.8901	0.036	14.80	1.30E-03	
-3.44	0.8340	0.034	15.63	1.14E-03	
-2.80	1.3248	0.030	16.96	9.02E-04	
-2.10	1.7418	0.036	18.70	1.27E-03	
-1.84	0.5945	0.012	19.29	1.38E-04	
-1.78	0.1664	0.003	19.46	9.61E-06	
-1.51	0.5987	0.015	20.06	2.24E-04	
-1.22	0.6193	0.014	20.68	2.08E-04	
-0.91	0.5626	0.016	21.24	2.60E-04	
-0.55	0.6082	0.021	21.85	4.52E-04	

-0.28	0.5457	0.017	22.39	2.85E-04
0.00	-0.1337	0.023	22.26	5.45E-04
0.18	-0.5976	0.028	21.66	7.83E-04
0.25	-0.6988	0.015	20.96	2.18E-04
0.30	-0.8385	0.011	20.13	1.16E-04
0.34	-0.7918	0.009	19.33	7.31E-05
0.39	-0.7700	0.007	18.56	5.01E-05
0.42	-0.6639	0.006	17.90	3.60E-05
0.47	-0.7231	0.006	17.18	3.69E-05
0.52	-0.6597	0.005	16.52	2.61E-05
0.58	-0.6757	0.005	15.84	2.57E-05
0.65	-0.6182	0.005	15.22	2.92E-05
0.73	-0.6209	0.006	14.60	3.59E-05
0.82	-0.6128	0.006	13.99	4.12E-05
0.94	-0.5952	0.007	13.39	5.51E-05
1.08	-0.6723	0.010	12.72	1.01E-04
1.23	-0.8245	0.013	11.90	1.71E-04
1.39	-1.1902	0.013	10.71	1.69E-04
1.54	-1.0229	0.013	9.68	1.67E-04
1.65	-0.7969	0.010	8.89	9.45E-05
1.91	-2.5671	0.022	6.32	4.95E-04
2.48	-6.0616	0.036	0.26	1.28E-03
3.01	-5.3092	0.020	-5.05	4.06E-04
3.46	-3.6025	0.023	-8.65	5.20E-04
3.87	-2.6386	0.030	-11.29	9.07E-04
4.24	-1.7928	0.030	-13.08	9.02E-04
4.62	-1.3643	0.025	-14.45	6.34E-04
4.98	-0.9993	0.022	-15.45	5.03E-04
5.33	-0.4906	0.023	-15.94	5.40E-04
5.93	-0.3854	0.019	-16.32	3.59E-04
6.23	0.0348	0.020	-16.29	4.01E-04
6.51	0.1271	0.018	-16.16	3.15E-04
6.66	0.0706	0.010	-16.09	1.07E-04
6.55	-0.8549	0.033	-16.95	1.12E-03
6.57	-0.7131	0.047	-17.66	2.17E-03
7.00	-0.1367	0.035	-17.80	1.23E-03
7.13	-0.1095	0.013	-17.91	1.60E-04

Table S13. Determined errors of computed free energies ($\Delta G^{\ddagger}$), for every step ($\sigma_{\Delta G_{i\rightarrow i+1}}$) and total free energy barrier ($\sigma^{\ddagger}$) explored using FEP method for second step of the acylation, "INT1(A)" to "PC(A)", involving proton transfer from Lys75 via Ser150 to the oxygen of pNC, leading to ester bond cleavage and departure of the leaving group. Results were obtained at M06-2X/AMBER level of theory and energy values are provided in kcal/mol.

s-coor (Å)	$\Delta G_{i\rightarrow i+1}$	$\sigma(\Delta G_{i\rightarrow i+1})$	FEP	$\sigma^2(\Delta G_{i\rightarrow i+1})$	$\sigma^{\ddagger}$
-1.05	0.0000	0.000	0.00	0	**0.06**
-1.06	-0.0994	0.018	-0.10	3.30E-04	
-0.78	0.2416	0.010	0.14	1.04E-04	
-0.33	0.8215	0.040	0.96	1.58E-03	
-0.14	0.7006	0.018	1.66	3.09E-04	
0.00	0.9557	0.030	2.62	8.77E-04	
0.14	-0.3146	0.032	2.31	1.03E-03	
0.20	-0.6891	0.015	1.62	2.13E-04	
0.26	-0.5909	0.012	1.03	1.48E-04	
0.35	-0.5388	0.009	0.49	8.74E-05	
0.48	-0.5368	0.008	-0.05	6.50E-05	
0.71	-0.4615	0.009	-0.51	8.63E-05	
1.16	-0.3471	0.015	-0.86	2.20E-04	
1.75	-0.1250	0.019	-0.98	3.71E-04	

Table S14. Determined errors of computed free energies ($\Delta G^{\ddagger}$), for every step ($\sigma_{\Delta G_{i \to i+1}}$) and total free energy barrier ($\sigma^{\ddagger}$) explored using FEP method for first step of the deacylation, "RC(H)" to "INT1(H)", involving activation of a water molecule by proton transfer through Ser150 to Lys75, followed by a nucleophilic attack of the water oxygen on the carbonyl carbon of pNC. Results were obtained at M06-2X/AMBER level of theory and energy values are provided in kcal/mol.

s-coor (Å)	$\Delta G_{i \to i+1}$	$\sigma(\Delta G_{i \to i+1})$	FEP	$\sigma^2(\Delta G_{i \to i+1})$	$\sigma^{\ddagger}$
-3.08	0.0000	0.000	0	0.00E+00	**0.97**
-3.07	0.0817	0.007	0.08	4.72E-05	
-3.08	0.1795	0.012	0.16	1.35E-04	
-3.03	0.3197	0.031	0.34	9.68E-04	
-3.04	0.0040	0.020	0.35	3.83E-04	
-3.12	-0.1259	0.021	0.22	4.27E-04	
-3.63	-1.1969	0.567	-0.98	3.21E-01	
-3.62	0.1559	0.096	-0.82	9.29E-03	
-3.45	0.6840	0.038	-0.14	1.44E-03	
-3.41	0.1640	0.021	0.03	4.35E-04	
-3.28	0.6125	0.036	0.64	1.26E-03	
-2.79	0.0487	0.587	0.69	3.44E-01	
-2.46	1.8055	0.043	2.49	1.87E-03	
-2.40	0.1928	0.090	2.69	8.18E-03	
-2.26	0.8336	0.020	3.52	4.00E-04	
-2.14	1.3127	0.041	4.83	1.66E-03	
-1.72	1.8503	0.046	6.68	2.12E-03	
-1.57	1.0848	0.017	7.77	2.83E-04	
-1.45	0.7008	0.012	8.47	1.36E-04	
-1.27	1.4641	0.034	9.93	1.18E-03	
-0.99	2.1307	0.027	12.06	7.41E-04	
-0.70	2.0528	0.023	14.12	5.32E-04	
-0.40	1.1135	0.415	15.23	1.72E-01	
0.00	0.4591	0.280	15.69	7.85E-02	
0.40	-0.0188	0.056	15.67	3.15E-03	
0.70	-1.2442	0.025	14.43	6.36E-04	
0.98	-1.2599	0.026	13.17	6.93E-04	
1.19	-0.7196	0.022	12.45	4.91E-04	
1.41	-0.3884	0.018	12.06	3.24E-04	
1.55	-0.4692	0.032	11.59	1.00E-03	
2.08	-0.4529	0.038	11.14	1.43E-03	
2.07	-0.0235	0.035	11.11	1.24E-03	

Table S15. Determined errors of computed free energies (ΔG‡), for every step ($\sigma_{\Delta G_{i\rightarrow i+1}}$) and total free energy barrier ($\sigma^{‡}$) explored using FEP method for second step of the deacylation, "INT1(H)" to "PC(H)" to, _involving Reactivation of the active site via cleavage of the Ser174–pNC linkage and protonation of the Ser174 residue. Results were obtained at M06-2X/AMBER level of theory and energy values are provided in kcal/mol.

s-coor (Å)	$\Delta G_{i\rightarrow i+1}$	$\sigma(\Delta G_{i\rightarrow i+1})$	FEP	$\sigma^2(\Delta G_{i\rightarrow i+1})$	σ‡
-4.11	0.0000	0.000	0.00	0	**0.60**
-4.09	0.0722	0.002	0.07	5.21E-06	
-4.05	0.1719	0.006	0.24	3.47E-05	
-4.02	0.1795	0.005	0.42	2.04E-05	
-3.99	0.1637	0.006	0.59	4.06E-05	
-3.98	0.0561	0.002	0.64	3.02E-06	
-3.94	0.0543	0.010	0.70	9.30E-05	
-4.00	0.0542	0.022	0.75	5.06E-04	
-4.00	0.0002	0.001	0.75	2.08E-06	
-3.98	-0.0280	0.020	0.72	3.91E-04	
-3.78	1.0494	0.038	1.77	1.46E-03	
-2.58	0.6365	0.016	2.41	2.51E-04	
-1.98	1.1488	0.023	3.56	5.11E-04	
-1.40	1.0811	0.025	4.64	6.03E-04	
-0.99	1.8505	0.026	6.49	7.02E-04	
-0.60	2.3375	0.088	8.83	7.76E-03	
0.00	0.1409	0.043	8.97	1.81E-03	
0.50	-2.9161	0.059	6.05	3.54E-03	
0.74	-5.0256	0.048	1.03	2.26E-03	
0.85	-2.5435	0.028	-1.52	7.82E-04	
0.91	-0.5585	0.011	-2.07	1.27E-04	
0.97	0.0302	0.007	-2.04	5.25E-05	
1.00	-0.8699	0.009	-2.91	7.97E-05	
1.05	-6.0870	0.587	-9.00	3.44E-01	
1.42	-1.4227	0.013	-10.42	1.60E-04	
1.67	-0.8120	0.010	-11.24	9.20E-05	
1.84	-0.3475	0.007	-11.58	4.51E-05	
1.97	-0.8161	0.009	-12.40	8.96E-05	
2.14	-1.3839	0.263	-13.78	6.93E-02	
2.40	-1.0667	0.016	-14.85	2.64E-04	
2.66	-0.8869	0.013	-15.74	1.57E-04	
2.91	-1.4477	0.018	-17.19	3.09E-04	
3.24	-0.5668	0.014	-17.75	2.03E-04	
3.46	-0.8159	0.028	-18.57	7.84E-04	
3.65	0.2447	0.021	-18.32	4.46E-04	
3.57	-0.8153	0.021	-19.14	4.50E-04	
3.75	-0.9975	0.026	-20.14	6.51E-04	
3.99	-0.7177	0.025	-20.85	6.48E-04	
4.19	0.0088	0.002	-20.84	2.87E-06	
4.19	-0.2990	0.009	-21.14	8.82E-05	
4.29	-0.1287	0.005	-21.27	2.65E-05	

4.33	-0.0191	0.012	-21.29	1.37E-04
4.30	0.1228	0.006	-21.17	3.67E-05
4.28	-0.7573	0.033	-21.93	1.11E-03
4.52	-0.0299	0.001	-21.96	9.66E-07
4.53	-0.1100	0.004	-22.07	1.31E-05
4.57	-0.2518	0.008	-22.32	6.11E-05
4.65	-0.0240	0.001	-22.34	1.06E-06
4.66	-0.0919	0.003	-22.43	7.33E-06
4.69	-0.0836	0.003	-22.52	7.03E-06
4.72	-0.0889	0.003	-22.61	7.69E-06
4.76	-0.0161	0.001	-22.62	2.94E-07
4.77	-0.0697	0.003	-22.69	7.17E-06
4.80	-0.1215	0.004	-22.81	1.38E-05
4.85	-0.1323	0.004	-22.95	1.77E-05

Table S16. Determined errors of computed free energies ($\Delta G^{\ddagger}$), for every step ($\sigma_{\Delta G_{i \to i+1}}$) and total free energy barrier ($\sigma^{\ddagger}$) explored using FEP method for first step of the decarboxylation, "RC(D)" to "INT1(D)". Results were obtained at M06-2X/AMBER level of theory and energy values are provided in kcal/mol.

s-coor (Å)	$\Delta G_{i \to i+1}$	$\sigma(\Delta G_{i \to i+1})$	FEP	$\sigma^2(\Delta G_{i \to i+1})$	$\sigma^{\ddagger}$
-4.37	0.0000	0.000	0.00	0	**0.37**
-4.53	0.0615	0.025	0.06	6.31E-04	
-4.37	-0.2261	0.047	-0.16	2.22E-03	
-3.78	0.0570	0.146	-0.11	2.14E-02	
-3.11	0.0978	0.060	-0.01	3.64E-03	
-2.34	1.1018	0.078	1.09	6.12E-03	
-2.14	0.3377	0.025	1.43	6.07E-04	
-1.62	2.0957	0.061	3.53	3.71E-03	
-1.32	1.5624	0.036	5.09	1.27E-03	
-1.00	2.2497	0.046	7.34	2.12E-03	
-0.89	1.3005	0.024	8.64	5.71E-04	
-0.66	2.7305	0.113	11.37	1.27E-02	
-0.30	3.2699	0.265	14.64	7.01E-02	
0.00	0.8341	0.092	15.47	8.50E-03	
0.39	-1.3479	0.107	14.12	1.14E-02	
0.68	-2.7272	0.058	11.40	3.40E-03	
0.84	-2.9502	0.035	8.45	1.22E-03	
0.93	-1.2597	0.016	7.19	2.61E-04	
0.93	-0.0737	0.010	7.11	9.59E-05	
0.97	-0.1816	0.013	6.93	1.66E-04	
1.26	-1.8307	0.030	5.10	8.72E-04	
1.46	-0.7817	0.020	4.32	4.16E-04	
1.67	-0.3196	0.022	4.00	4.90E-04	
1.91	-0.2011	0.020	3.80	3.93E-04	
2.46	-0.2148	0.035	3.58	1.19E-03	
2.76	-0.4085	0.021	3.18	4.51E-04	

Table S17. Determined errors of computed free energies ($\Delta G^{\ddagger}$), for every step ($\sigma_{\Delta G_{i \to i+1}}$) and total free energy barrier ($\sigma^{\ddagger}$) explored using FEP method for second step of the decarboxylation, "INT1(D)" to "INT2(D)". Results were obtained at M06-2X/AMBER level of theory and energy values are provided in kcal/mol.

s-coor (Å)	$\Delta G_{i \to i+1}$	$\sigma(\Delta G_{i \to i+1})$	FEP	$\sigma^2(\Delta G_{i \to i+1})$	$\sigma^{\ddagger}$
-2.03	0.0000	0.000	0.00	0.00	**0.10**
-1.53	0.0805	0.037	0.08	1.36E-03	
-1.06	0.3194	0.057	0.40	3.21E-03	
-0.82	0.5685	0.024	0.97	5.58E-04	
-0.69	0.6302	0.014	1.60	1.94E-04	
-0.61	0.6993	0.008	2.30	6.79E-05	
-0.55	0.7443	0.008	3.04	6.64E-05	
-0.50	0.7511	0.007	3.79	5.21E-05	
-0.46	0.8398	0.009	4.63	7.40E-05	
-0.42	1.2060	0.017	5.84	2.74E-04	
-0.35	1.1952	0.023	7.03	5.09E-04	
-0.25	1.1353	0.026	8.17	6.85E-04	
0.00	0.2189	0.057	8.39	3.24E-03	
0.56	-3.0619	0.146	5.33	2.12E-02	
0.85	-0.2789	0.021	5.05	4.25E-04	
1.15	-1.0716	0.035	3.98	1.26E-03	
1.90	-0.9327	0.067	3.04	4.46E-03	
2.00	-0.0386	0.022	3.00	4.94E-04	
2.04	0.0132	0.003	3.02	8.21E-06	

Table S18. Determined errors of computed free energies ($\Delta G^{\ddagger}$), for every step ($\sigma_{\Delta G_{i \rightarrow i+1}}$) and total free energy barrier ($\sigma^{\ddagger}$) explored using FEP method for third step of the decarboxylation, "INT2(D)" to "PC(D)". Results were obtained at M06-2X/AMBER level of theory and energy values are provided in kcal/mol.

s-coor (Å)	$\Delta G_{i \rightarrow i+1}$	$\sigma(\Delta G_{i \rightarrow i+1})$	FEP	$\sigma^2(\Delta G_{i \rightarrow i+1})$	$\sigma^{\ddagger}$
-2.43	0.0000	0.000	0.00	0	**0.27**
-2.70	-0.0968	0.046	-0.10	2.14E-03	
-2.72	-0.0077	0.001	-0.10	1.15E-06	
-2.70	0.0083	0.001	-0.10	1.79E-06	
-2.83	-0.0235	0.007	-0.12	5.46E-05	
-2.84	-0.0311	0.002	-0.15	3.48E-06	
-2.13	0.7514	0.058	0.60	3.36E-03	
-2.54	-0.4582	0.042	0.14	1.76E-03	
-2.64	-0.0092	0.009	0.13	8.79E-05	
-3.18	0.0818	0.030	0.21	9.01E-04	
-2.61	-0.5739	0.055	-0.36	3.00E-03	
-2.77	-0.0196	0.014	-0.38	1.85E-04	
-3.26	0.1092	0.029	-0.27	8.24E-04	
-2.57	-0.2567	0.043	-0.53	1.86E-03	
-2.39	0.0351	0.019	-0.49	3.54E-04	
-2.08	0.7709	0.051	0.28	2.57E-03	
-1.85	-0.0799	0.070	0.20	4.89E-03	
-1.00	2.4354	0.168	2.64	2.83E-02	
0.00	3.9387	0.139	6.57	1.93E-02	
0.47	0.1729	0.051	6.75	2.62E-03	
0.70	-0.3534	0.022	6.39	5.03E-04	
0.91	-0.4388	0.017	5.95	2.81E-04	
1.12	-0.4830	0.018	5.47	3.07E-04	
1.37	-0.5859	0.013	4.89	1.82E-04	
1.69	-0.5525	0.016	4.33	2.46E-04	
1.90	-0.2282	0.009	4.11	7.96E-05	

Key distances (in Å) for the states located along the reaction progress.

Table S19. Key distances (in Å) for the states located along the rotation of the residue Ser174 of metagenome-derived urethanase UMG-SP-2 by the substrate, optimized at M06-2X/6-31+G(d,p)/MM level of theory.

	$ES_{innactive}$	TS0	ES_{active}
	Active Site		
$O2_{SUB}$-HG_{S174}	1.80	2.64	2.89
$C1_{SUB}$-OG_{S174}	3.64	3.23	3.16
OG_{S174}-HG_{S174}	0.97	0.97	0.97
OG_{S150}-HG_{S150}	0.99	0.99	0.99
NZ_{K75}-HG_{S150}	1.85	1.88	1.88
dihedrals			
$(C\text{-}CA\text{-}CB\text{-}OG)_{S174}$	-126.9	178.9	156.3
$(CA\text{-}CB\text{-}OG)_{S174}$-C1	-141.4	-162.0	166.6

Table S20. Key distances (in Å) for the states located along the acylation of metagenome-derived urethanase UMG-SP-2 by the substrate, optimized at M06-2X/6-31+G(d,p)/MM level of theory.

	ES_{active}	TS1(A)	Int1(A)	TS2(A)	Int2(A)
	Active Site				
OG_{S174}-HG_{S174}	0.97	1.16	2.62	2.63	2.53
OG_{S150}-HG_{S174}	1.92	1.24	1.04	1.22	1.56
OG_{S150}-HG_{S150}	1.00	1.10	1.58	1.32	1.03
NZ_{K75}-HG_{S150}	1.80	1.48	1.08	1.20	1.65
$C1_{SUB}$-OG_{S174}	2.90	1.65	1.34	1.33	1.33
$O3_{SUB}$-HG_{S174}	3.05	2.41	1.46	1.19	1.01
$C1_{SUB}$-$O3_{SUB}$	1.38	1.49	2.77	2.78	2.79
$O3_{SUB}$-HG_{S174}	3.05	2.41	1.46	1.19	1.01
$C1_{SUB}$-$O2_{SUB}$	1.21	1.25	1.23	1.23	1.23
	Oxyanion Hole				
$O2_{SUB}$-H_{I171}	3.52	2.28	2.66	2.66	2.64
$O2_{SUB}$-HG_{172}	2.73	1.80	1.79	1.79	1.78
$O2_{SUB}$-H_{S174}	4.19	2.47	2.45	2.52	2.52

Table S21. Key distances (in Å) for the states located along the hydrolysis of metagenome-derived urethanase UMG-SP-2 by the substrate, optimized at M06-2X/6-31+G(d,p)/MM level of theory.

	Int1(H)	TS1(H)	Int2(H)	TS2(H)	Int3(H)
	Active Site				
$C1_{SUB}$-OG_{S174}	1.31	1.36	1.58	1.83	2.36
$C1_{SUB}$-O_{W}	2.62	1.85	1.40	1.37	1.33
O_{W}-$H1_{W}$	0.98	1.23	2.69	2.79	2.90
OG_{S174}-$H1_{W}$	2.66	2.54	1.60	1.45	1.01
OG_{S150}-$H1_{W}$	1.78	1.19	1.00	1.05	1.49
OG_{S150}-HG_{S150}	1.03	1.49	1.63	1.57	1.05
NZ_{K75}-HG_{S150}	1.65	1.12	1.06	1.08	1.56
$C1_{SUB}$- $O2_{SUB}$	1.25	1.27	1.30	1.27	1.24
	Oxyanion Hole				
$O2_{SUB}$-H_{I171}	2.04	2.12	2.23	2.25	2.23
$O2_{SUB}$-H_{G172}	1.75	1.84	1.86	1.83	1.75

O2$_{SUB}$-H$_{G173}$	2.43	2.64	2.25	2.28	2.32
O2$_{SUB}$-H $_{S174}$	2.30	2.47	2.01	2.10	2.24

Table S22. Key distances (in Å) for the states located along the decarboxylation of metagenome-derived urethanase UMG-SP-2 by the substrate, optimized at M06-2X/6-31+G(d,p)/MM level of theory.

	RC(D)	TS1(D)	Int1(D)	TS2(D)	Int2(D)	TS3(D)	PC(D)
	Active Site						
C1$_{SUB}$-OG$_{S174}$	2.42	2.52	2.71	2.74	2.53	2.44	2.39
C1$_{SUB}$-O$_{w}$	1.33	1.33	1.23	1.21	1.20	1.17	1.16
O$_{w}$-H2$_{w}$	0.97	1.01	2.85	2.84	2.96	3.12	2.93
OG$_{S174}$-H2$_{w}$	3.14	1.64	1.00	1.48	1.93	2.19	2.20
N4$_{SUB}$-H2$_{w}$	3.08	2.83	1.67	1.10	1.03	1.02	1.02
OG$_{S174}$-H1$_{w}$	1.01	1.22	1.48	1.15	1.01	1.00	1.00
OG$_{S150}$-H1$_{w}$	1.52	1.16	1.01	1.23	1.50	1.53	1.53
OG$_{S150}$-HG$_{S150}$	1.04	1.40	1.57	1.38	1.06	1.05	1.05
NZ$_{K75}$-HG$_{S150}$	1.58	1.13	1.07	1.15	1.53	1.54	1.56
C1$_{SUB}$- O2$_{SUB}$	1.24	1.24	1.28	1.25	1.23	1.20	1.17
C1$_{SUB}$- N4$_{SUB}$	1.36	1.34	1.45	1.56	1.64	2.09	2.68
	Oxyanion Hole						
O2$_{SUB}$-H$_{I171}$	2.22	2.28	2.87	2.94	2.84	2.66	2.51
O2$_{SUB}$-H$_{G172}$	1.77	1.73	1.80	1.82	1.81	1.76	1.75
O2$_{SUB}$-H$_{G173}$	2.43	2.17	1.84	1.85	1.87	2.06	2.24
O2$_{SUB}$-H$_{S174}$	2.46	2.19	2.03	2.12	2.11	2.16	2.20

Time evolution of interatomic distance between Oγ-Ser174 and C1 atom of the substrate in the product of the hydrolysis step.

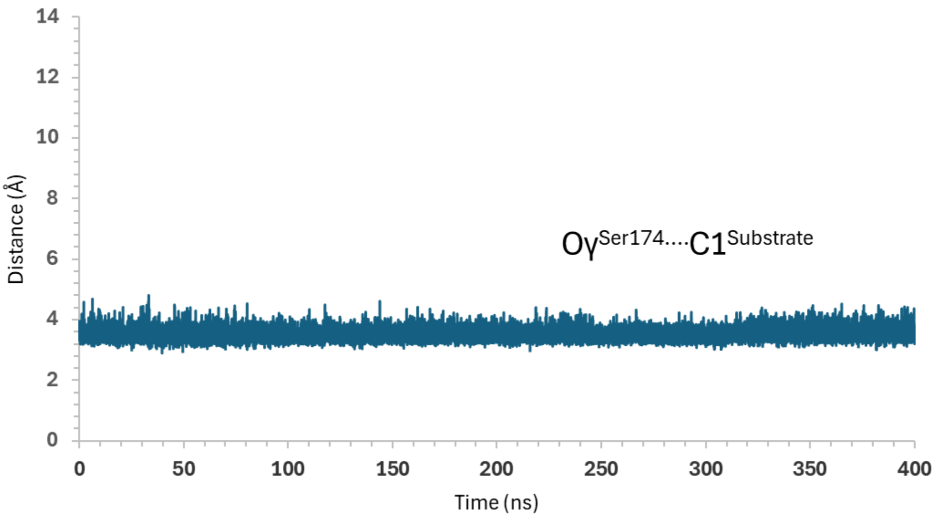

Figure S22. Time evolution of interatomic distance between Oγ-Ser174 and C1 atom of the substrate along 400 ns of unbiased MD simulations in the carbamic acid PC(H), the product of the hydrolysis step.

Occupancy analysis of water molecules inside the active site of the enzyme during the deacylation step

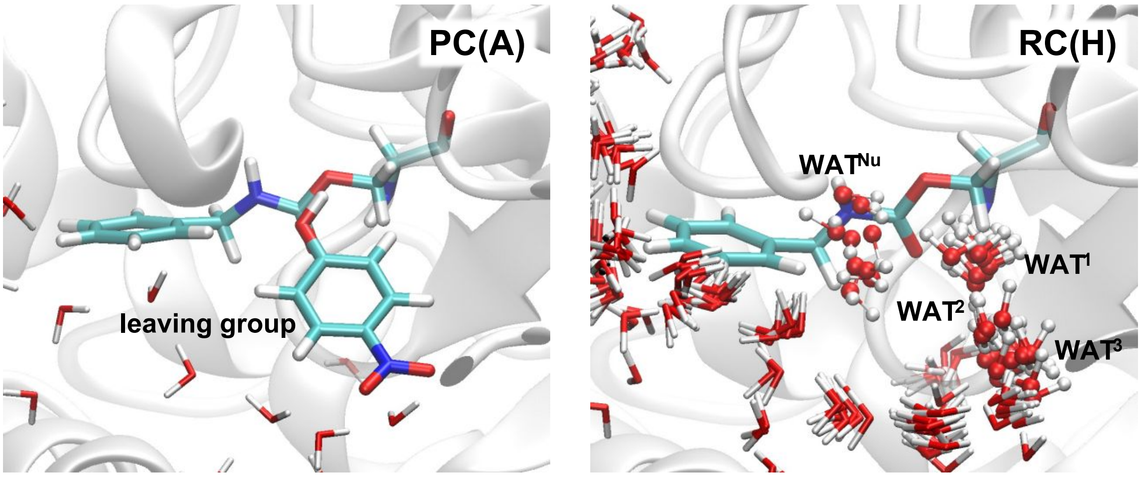

Figure S23. Position of the water molecules inside the active site of the enzyme at the end of the acylation steps. Left panel: M06-2X/MM optimized structure of PC(A); Right panel: overlaying water molecules around the active site as obtained in the QM/MM MD of the RC(H) after replacing the leaving group by 4 water molecules (in balls and sticks representation).

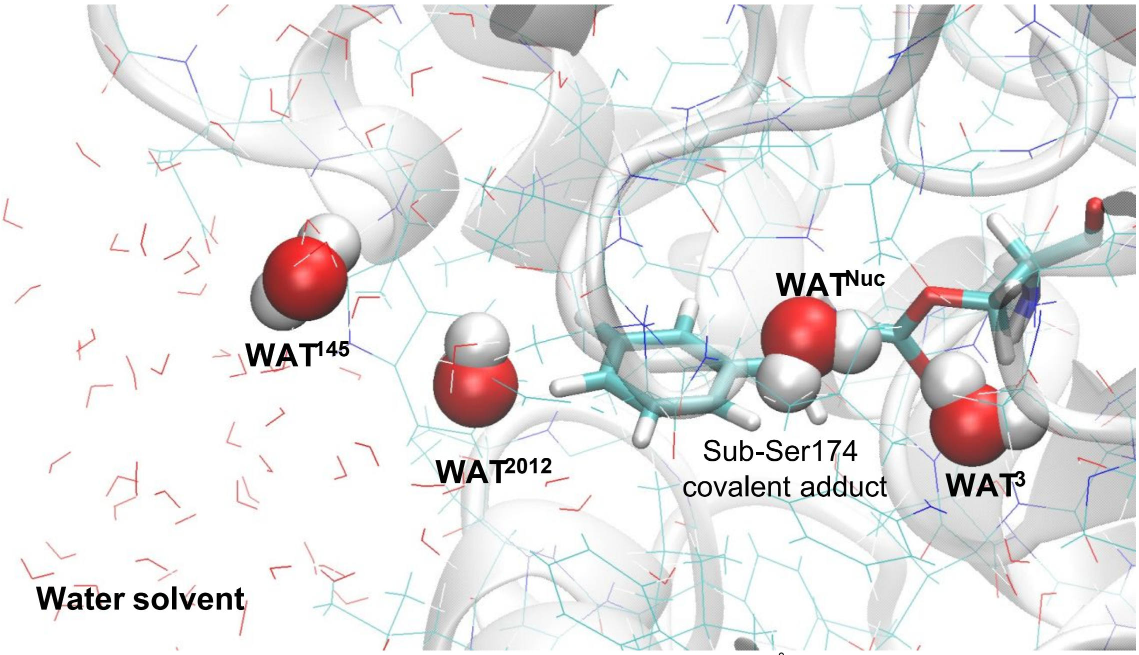

Figure S24. Preserved water molecules located within 6 Å from the substrate in the active site during the two steps of the deacylation process. WAT^{145}, WAT^{2012} and WAT^{3} are those remaining at less than 6 Å from the substrate in the active site. See Table S15.

Table S23. Occupancy of water molecules, measured as those whose O atoms are located within 6 Å from the substrate in the active site. Occupancy values represent the percentage of frames in FEP DFT/MM MD simulations where each water molecule was found within the specified distance, calculated over the full trajectory for 2000 snapshots. NUC stands for nucleophilic water, and WAT2 and WAT3 refer to water molecules added to the initial model during the deacylation step.

			**RC(H)**		**TS1(H)**		**INT1(H)**		**INT1(H)**		**TS2(H)**		**PC(H)**	
	Resid	Segname	Occupancy	%	Occupancy	%	Occupancy	%	Occupancy	%	Occupancy	%	Occupancy	%
**1**	**1**	**NUC**	**2000**	**100**	**2000**	**100**	**2000**	**100**	**2000**	**100**	**2000**	**100**	**2000**	**100**
2	2	WAT	2000	100	1897	95	1981	99	1278	64	992	50	862	43
**3**	**3**	**WAT**	**2000**	**100**	**2000**	**100**	**2000**	**100**	**2000**	**100**	**2000**	**100**	**2000**	**100**
**4**	**145**	**BOX**	**2000**	**100**	**2000**	**100**	**2000**	**100**	**2000**	**100**	**2000**	**100**	**961**	**48**
5	193	BOX							987	49	242	12	777	39
6	299	BOX	1835	92	699	35	1879	94	399	20	369	18	957	48
7	1271	BOX	39	2	91	5	121	6	304	15	30	1	1650	82
8	1331	BOX	2000	100	1998	100	2000	100	62	3			1838	92
9	1442	BOX	1531	77	964	48	226	11						
**10**	**2012**	**BOX**	**2000**	**100**	**2000**	**100**	**2000**	**100**	**2000**	**100**	**2000**	**100**	**2000**	**100**
11	2193	BOX	1033	52	2000	100	2000	100	99	5			815	41
12	2295	BOX							151	8	183	9	685	34
13	2694	BOX	1457	73	744	37	2000	100						
14	3871	BOX	1091	55	850	43	653	33	1082	54	980	49	1096	55
15	3960	BOX	16	1			529	26	115	6	35	2	217	11
16	4088	BOX												
17	5093	BOX	24	1	567	28	782	39						
18	5536	BOX	30	1	46	2	293	15						
19	7060	BOX							4	0	1571	79		
20	8191	BOX			17	1					905	45		

* Four water molecules (highlighted in the table) are preserved within a radius of 6 Å with an occupancy higher than 90% along the deacylation pathway.

Table S24. Cartesian coordinates (in Å) of QM atoms corresponding to the acylation of metagenome-derived urethanase UMG-SP-2 with 4-nitrophenyl benzylcarbamate (pNC) substrate, optimized at M06-2X/6-31+G(d,p)/MM level of theory.

ES inactive				TS0 (vi = 95.5i cm^{-1})				ES active			
Atoms	x	y	z	Atoms	x	y	z	Atoms	x	y	z
C	44.370	51.512	39.748	C	44.362	51.497	39.742	C	44.358	51.491	39.731
H	45.432	51.532	39.482	H	45.423	51.511	39.473	H	45.419	51.504	39.463
H	43.883	50.859	39.014	H	43.867	50.852	39.008	H	43.865	50.854	38.987
C	44.205	50.881	41.140	C	44.193	50.867	41.134	C	44.186	50.835	41.113
H	44.719	51.471	41.907	H	44.710	51.457	41.900	H	44.694	51.417	41.891
H	43.143	50.859	41.414	H	43.131	50.853	41.407	H	43.123	50.806	41.380
C	44.746	49.446	41.183	C	44.725	49.429	41.182	C	44.731	49.398	41.148
H	44.203	48.839	40.446	H	44.179	48.823	40.447	H	44.193	48.795	40.406
H	45.803	49.445	40.879	H	45.782	49.421	40.877	H	45.789	49.400	40.849
C	44.626	48.767	42.554	C	44.603	48.759	42.558	C	44.606	48.715	42.520
H	43.605	48.884	42.935	H	43.582	48.878	42.938	H	43.582	48.822	42.893
H	45.296	49.267	43.262	H	45.271	49.266	43.263	H	45.265	49.224	43.233
N	44.935	47.329	42.567	N	44.912	47.320	42.586	N	44.929	47.278	42.547
H	45.911	47.150	42.326	H	45.888	47.141	42.341	H	45.908	47.106	42.310
H	44.370	46.859	41.855	H	44.348	46.847	41.878	H	44.373	46.802	41.833
C	40.334	44.085	44.683	C	40.307	44.048	44.666	C	40.309	44.044	44.676
O	40.151	42.878	44.467	O	40.094	42.847	44.442	O	40.093	42.840	44.470
N	41.507	44.710	44.578	N	41.500	44.641	44.576	N	41.503	44.632	44.583
H	42.284	44.177	44.207	H	42.266	44.070	44.239	H	42.291	44.018	44.393
C	41.726	46.137	44.844	C	41.739	46.065	44.841	C	41.744	46.059	44.838
H	40.959	46.437	45.569	H	40.967	46.375	45.558	H	40.980	46.375	45.561
C	43.065	46.286	45.563	C	43.063	46.217	45.584	C	43.071	46.208	45.576
H	43.196	47.344	45.816	H	43.185	47.276	45.840	H	43.199	47.265	45.833
H	43.042	45.710	46.497	H	43.030	45.637	46.514	H	43.040	45.625	46.505
O	44.156	45.788	44.818	O	44.174	45.734	44.856	O	44.187	45.725	44.850
H	44.383	46.392	44.062	H	44.401	46.350	44.109	H	44.407	46.321	44.085
C	42.825	39.780	42.290	C	42.894	39.793	42.286	C	42.893	39.795	42.295
O	41.715	39.925	41.774	O	41.854	39.929	41.643	O	41.858	39.930	41.645
N	43.661	40.796	42.519	N	43.695	40.822	42.612	N	43.684	40.829	42.635
H	44.514	40.563	43.007	H	44.370	40.602	43.332	H	44.397	40.601	43.313
C	43.281	42.217	42.554	C	43.281	42.238	42.577	C	43.280	42.249	42.581
H	42.303	42.241	43.057	H	42.312	42.283	43.098	H	42.306	42.307	43.088
C	44.331	42.934	43.460	C	44.314	43.012	43.368	C	44.280	43.056	43.381
H	43.859	43.352	44.354	H	44.025	44.060	43.434	H	44.167	44.111	43.126
H	44.791	43.757	42.915	H	45.280	43.013	42.863	H	45.313	42.788	43.116
O	45.371	42.053	43.946	O	44.497	42.441	44.671	O	44.088	42.852	44.781
H	45.576	42.332	44.854	H	44.379	43.130	45.340	H	44.294	43.696	45.214
O	50.849	45.432	43.112	O	50.843	45.289	43.070	O	50.880	45.347	43.080
N	50.810	45.761	44.282	N	50.817	45.726	44.206	N	50.850	45.750	44.228
O	51.731	46.320	44.868	O	51.730	46.366	44.714	O	51.764	46.366	44.761
C	49.609	45.458	45.059	C	49.647	45.452	45.035	C	49.672	45.464	45.041
C	48.414	45.189	44.395	C	48.438	45.126	44.424	C	48.478	45.117	44.411
C	47.283	44.899	45.153	C	47.343	44.847	45.235	C	47.374	44.828	45.203
H	46.312	44.752	44.689	H	46.363	44.645	44.813	H	46.407	44.609	44.761
H	48.352	45.271	43.313	H	48.343	45.156	43.343	H	48.403	45.136	43.328
C	49.724	45.462	46.445	C	49.806	45.527	46.415	C	49.804	45.552	46.423
H	50.675	45.684	46.917	H	50.766	45.790	46.844	H	50.753	45.833	46.868
C	48.598	45.151	47.191	C	48.712	45.229	47.213	C	48.700	45.245	47.203
H	48.636	45.116	48.274	H	48.786	45.243	48.295	H	48.752	45.271	48.286
C	47.399	44.868	46.542	C	47.502	44.882	46.621	C	47.505	44.876	46.592
O	46.310	44.679	47.358	O	46.435	44.693	47.461	O	46.430	44.678	47.415
C	45.541	43.554	47.259	C	45.765	43.490	47.449	C	45.756	43.472	47.389
O	45.874	42.566	46.615	O	46.257	42.453	47.060	O	46.235	42.449	46.948
N	44.443	43.706	48.001	N	44.524	43.677	47.941	N	44.544	43.650	47.942
H	44.374	44.587	48.515	H	44.368	44.581	48.391	H	44.399	44.546	48.412
C	43.612	42.608	48.452	C	43.708	42.562	48.385	C	43.735	42.525	48.369
H	43.787	41.759	47.785	H	43.932	41.711	47.735	H	43.982	41.683	47.717

H	42.563	42.904	48.359	H	42.658	42.832	48.237	H	42.683	42.775	48.207
C	43.917	42.227	49.892	C	43.943	42.190	49.837	C	43.964	42.161	49.825
C	42.937	41.580	50.647	C	42.911	41.587	50.558	C	42.949	41.510	50.529
H	41.975	41.349	50.198	H	41.956	41.397	50.073	H	42.014	41.272	50.028
C	43.198	41.201	51.959	C	43.110	41.194	51.877	C	43.142	41.132	51.852
H	42.434	40.683	52.530	H	42.306	40.704	52.417	H	42.351	40.606	52.378
C	44.438	41.460	52.542	C	44.341	41.400	52.499	C	44.348	41.401	52.499
H	44.641	41.137	53.559	H	44.500	41.059	53.518	H	44.502	41.075	53.523
C	45.415	42.114	51.799	C	45.370	42.013	51.790	C	45.360	42.061	51.807
H	46.382	42.330	52.246	H	46.331	42.182	52.267	H	46.300	42.282	52.303
C	45.156	42.494	50.483	C	45.172	42.404	50.467	C	45.169	42.436	50.478
H	45.928	43.008	49.917	H	45.988	42.177	49.927	H	45.969	42.948	49.951

ES active				TS1(A) (νi = 523.5i cm^{-1})				Int1(A)			
Atoms	x	y	z	Atoms	x	y	z	Atoms	x	y	z
C	44.344	51.414	39.692	C	44.402	51.438	39.740	C	44.401	51.437	39.741
H	45.401	51.423	39.404	H	45.457	51.473	39.449	H	45.460	51.477	39.464
H	43.831	50.789	38.951	H	43.909	50.785	39.011	H	43.915	50.789	39.002
C	44.180	50.767	41.071	C	44.270	50.801	41.132	C	44.255	50.790	41.127
H	44.686	51.353	41.847	H	44.790	51.397	41.889	H	44.764	51.380	41.897
H	43.117	50.734	41.339	H	43.214	50.769	41.422	H	43.197	50.741	41.407
C	44.731	49.338	41.103	C	44.821	49.367	41.180	C	44.828	49.364	41.146
H	44.210	48.743	40.344	H	44.292	48.762	40.433	H	44.302	48.768	40.390
H	45.796	49.346	40.826	H	45.883	49.363	40.902	H	45.888	49.388	40.866
C	44.572	48.661	42.468	C	44.656	48.710	42.556	C	44.683	48.685	42.506
H	43.536	48.767	42.808	H	43.637	48.870	42.922	H	43.657	48.751	42.878
H	45.208	49.177	43.195	H	45.346	49.175	43.267	H	45.354	49.137	43.239
N	44.900	47.227	42.505	N	44.897	47.247	42.578	N	45.008	47.223	42.453
H	45.883	47.067	42.270	H	45.879	47.036	42.366	H	46.007	47.035	42.268
H	44.357	46.753	41.777	H	44.351	46.821	41.821	H	44.462	46.771	41.705
C	40.259	44.208	44.742	C	40.253	44.212	44.752	C	40.255	44.215	44.761
O	40.087	42.991	44.556	O	40.083	43.001	44.551	O	40.096	42.996	44.580
N	41.423	44.836	44.573	N	41.433	44.830	44.625	N	41.417	44.849	44.606
H	42.215	44.235	44.361	H	42.214	44.211	44.436	H	42.213	44.248	44.446
C	41.657	46.271	44.797	C	41.689	46.262	44.828	C	41.674	46.276	44.834
H	40.891	46.596	45.511	H	40.944	46.607	45.553	H	40.920	46.614	45.553
C	42.991	46.435	45.517	C	43.038	46.455	45.510	C	43.021	46.422	45.536
H	43.127	47.502	45.720	H	43.148	47.513	45.743	H	43.124	47.464	45.854
H	42.951	45.897	46.472	H	43.092	45.885	46.445	H	43.055	45.787	46.431
O	44.098	45.901	44.818	O	44.157	46.033	44.723	O	44.106	46.062	44.689
H	44.347	46.438	44.006	H	44.433	46.593	43.822	H	44.707	46.760	43.374
C	42.777	39.936	42.381	C	42.814	39.967	42.443	C	42.786	39.952	42.421
O	41.756	40.048	41.704	O	41.812	40.088	41.734	O	41.757	40.074	41.754
N	43.535	40.989	42.736	N	43.554	41.018	42.857	N	43.540	40.998	42.795
H	44.255	40.781	43.412	H	44.244	40.830	43.577	H	44.309	40.803	43.425
C	43.147	42.414	42.620	C	43.186	42.446	42.693	C	43.120	42.410	42.665
H	42.164	42.506	43.101	H	42.222	42.586	43.200	H	42.114	42.477	43.104
C	44.151	43.237	43.391	C	44.261	43.317	43.336	C	44.060	43.261	43.490
H	44.069	44.280	43.075	H	44.276	44.279	42.821	H	43.934	44.311	43.240
H	45.178	42.928	43.150	H	45.242	42.860	43.228	H	45.111	43.005	43.359
O	43.934	43.113	44.789	O	44.115	43.645	44.732	O	43.719	43.163	44.890
H	44.145	43.980	45.173	H	44.233	44.801	44.778	H	44.776	45.476	45.222
O	50.988	45.238	42.841	O	50.972	45.848	43.580	O	51.257	45.993	43.533
N	50.894	45.747	43.943	N	50.665	45.945	44.759	N	50.937	45.972	44.724
O	51.750	46.475	44.432	O	51.396	46.449	45.606	O	51.703	46.360	45.614
C	49.713	45.459	44.749	C	49.367	45.435	45.181	C	49.639	45.515	45.087
C	48.537	45.049	44.124	C	48.438	45.068	44.210	C	48.707	45.242	44.074
C	47.423	44.784	44.911	C	47.205	44.564	44.598	C	47.408	44.930	44.399
H	46.470	44.522	44.459	H	46.486	44.292	43.838	H	46.681	44.733	43.615
H	48.480	44.999	43.043	H	48.669	45.204	43.161	H	49.016	45.318	43.039
C	49.824	45.608	46.127	C	49.081	45.327	46.542	C	49.272	45.410	46.442
H	50.759	45.933	46.569	H	49.826	45.619	47.275	H	50.019	45.596	47.206
C	48.719	45.310	46.907	C	47.855	44.810	46.924	C	47.979	45.075	46.771

H	48.753	45.386	47.987	H	47.609	44.684	47.973	H	47.670	44.997	47.809
C	47.533	44.902	46.299	C	46.909	44.407	45.962	C	46.968	44.889	45.765
O	46.459	44.744	47.125	O	45.746	43.923	46.440	O	45.738	44.719	46.070
C	45.711	43.580	47.095	C	45.026	42.788	45.804	C	44.441	42.349	45.659
O	46.145	42.519	46.701	O	45.728	41.955	45.196	O	45.397	41.713	45.217
N	44.485	43.855	47.573	N	44.029	42.376	46.724	N	43.965	42.282	46.915
H	44.384	44.766	48.018	H	43.488	43.153	47.090	H	43.157	42.848	47.139
C	43.593	42.791	47.998	C	44.384	41.388	47.744	C	44.409	41.279	47.865
H	43.664	41.985	47.264	H	45.367	40.983	47.470	H	45.432	41.014	47.579
H	42.571	43.186	47.960	H	43.668	40.559	47.702	H	43.789	40.375	47.782
C	43.914	42.288	49.395	C	44.435	41.870	49.188	C	44.390	41.754	49.301
C	43.620	40.972	49.742	C	43.819	41.115	50.195	C	43.801	40.965	50.294
H	43.171	40.310	49.005	H	43.233	40.237	49.929	H	43.267	40.057	50.021
C	43.864	40.499	51.029	C	43.935	41.477	51.537	C	43.883	41.338	51.636
H	43.608	39.472	51.272	H	43.462	40.869	52.304	H	43.441	40.705	52.401
C	44.425	41.336	51.990	C	44.662	42.611	51.898	C	44.546	42.511	51.995
H	44.620	40.958	52.990	H	44.756	42.900	52.944	H	44.621	42.807	53.039
C	44.725	42.655	51.651	C	45.250	43.382	50.897	C	45.101	43.314	50.997
H	45.154	43.315	52.400	H	45.797	44.284	51.156	H	45.602	44.241	51.260
C	44.469	43.130	50.365	C	45.134	43.022	49.556	C	45.021	42.948	49.657
H	44.690	44.166	50.110	H	45.562	43.656	48.786	H	45.425	43.599	48.884

Int1(A)				TS2(A) (νi = 933.9i cm^{-1})				PC(A)			
Atoms	x	y	z	Atoms	x	y	z	Atoms	x	y	z
C	44.405	51.439	39.753	C	44.407	51.438	39.755	C	44.402	51.438	39.738
H	45.464	51.481	39.481	H	45.466	51.476	39.478	H	45.459	51.474	39.457
H	43.922	50.785	39.018	H	43.924	50.781	39.023	H	43.911	50.787	39.005
C	44.251	50.804	41.144	C	44.264	50.803	41.150	C	44.252	50.804	41.126
H	44.763	51.395	41.910	H	44.780	51.399	41.909	H	44.765	51.399	41.890
H	43.192	50.768	41.421	H	43.206	50.771	41.432	H	43.192	50.774	41.404
C	44.809	49.373	41.172	C	44.815	49.369	41.198	C	44.801	49.375	41.166
H	44.276	48.780	40.420	H	44.291	48.766	40.448	H	44.270	48.773	40.419
H	45.869	49.386	40.888	H	45.879	49.368	40.928	H	45.863	49.375	40.884
C	44.663	48.701	42.535	C	44.648	48.710	42.571	C	44.645	48.711	42.536
H	43.638	48.778	42.910	H	43.624	48.834	42.939	H	43.617	48.843	42.892
H	45.340	49.151	43.265	H	45.331	49.164	43.293	H	45.309	49.205	43.254
N	44.975	47.236	42.485	N	44.915	47.243	42.565	N	44.934	47.266	42.561
H	45.969	47.040	42.288	H	45.903	47.034	42.366	H	45.914	47.087	42.320
H	44.418	46.789	41.742	H	44.364	46.803	41.816	H	44.378	46.812	41.830
C	40.305	44.197	44.757	C	40.311	44.201	44.759	C	40.306	44.196	44.757
O	40.159	42.976	44.582	O	40.163	42.979	44.584	O	40.162	42.974	44.580
N	41.460	44.843	44.621	N	41.469	44.842	44.626	N	41.461	44.842	44.625
H	42.271	44.255	44.489	H	42.276	44.251	44.483	H	42.271	44.265	44.449
C	41.690	46.272	44.845	C	41.715	46.272	44.841	C	41.686	46.272	44.841
H	40.929	46.603	45.560	H	40.977	46.607	45.578	H	40.928	46.604	45.559
C	43.033	46.437	45.549	C	43.084	46.436	45.498	C	43.035	46.446	45.528
H	43.130	47.486	45.848	H	43.176	47.486	45.794	H	43.157	47.506	45.762
H	43.068	45.820	46.456	H	43.135	45.828	46.413	H	43.059	45.880	46.468
O	44.119	46.063	44.710	O	44.160	46.047	44.658	O	44.119	45.965	44.747
H	44.679	46.769	43.412	H	44.557	46.722	43.590	H	44.397	46.550	43.949
C	42.810	39.901	42.396	C	42.811	39.900	42.394	C	42.811	39.900	42.395
O	41.780	40.028	41.731	O	41.780	40.028	41.732	O	41.776	40.027	41.741
N	43.557	40.951	42.783	N	43.562	40.948	42.777	N	43.566	40.948	42.775
H	44.307	40.746	43.432	H	44.316	40.738	43.420	H	44.333	40.736	43.400
C	43.152	42.369	42.669	C	43.161	42.368	42.668	C	43.166	42.368	42.667
H	42.141	42.441	43.096	H	42.153	42.441	43.103	H	42.161	42.445	43.106
C	44.087	43.193	43.537	C	44.104	43.180	43.538	C	44.116	43.191	43.516
H	44.024	44.244	43.263	H	44.052	44.234	43.275	H	44.058	44.240	43.237
H	45.132	42.886	43.470	H	45.146	42.862	43.472	H	45.159	42.876	43.439
O	43.670	43.139	44.921	O	43.684	43.113	44.923	O	43.715	43.145	44.910
H	44.790	45.480	45.258	H	44.953	45.382	45.315	H	45.115	45.130	45.614
O	51.258	45.856	43.536	O	51.269	45.836	43.555	O	51.225	45.829	43.489
N	50.944	45.879	44.728	N	50.946	45.850	44.741	N	50.950	45.863	44.681

O	51.724	46.275	45.602	O	51.709	46.235	45.629	O	51.743	46.235	45.543
C	49.635	45.464	45.106	C	49.618	45.439	45.100	C	49.619	45.470	45.095
C	48.692	45.198	44.102	C	48.689	45.218	44.079	C	48.634	45.321	44.119
C	47.388	44.922	44.439	C	47.370	44.968	44.395	C	47.334	45.046	44.503
H	46.652	44.731	43.661	H	46.640	44.815	43.605	H	46.552	44.963	43.753
H	48.997	45.247	43.064	H	49.006	45.285	43.046	H	48.873	45.470	43.074
C	49.271	45.394	46.464	C	49.245	45.341	46.449	C	49.335	45.310	46.454
H	50.026	45.579	47.222	H	49.993	45.490	47.220	H	50.127	45.427	47.185
C	47.972	45.092	46.806	C	47.930	45.063	46.766	C	48.039	45.009	46.834
H	47.666	45.036	47.846	H	47.607	44.991	47.799	H	47.774	44.885	47.878
C	46.955	44.906	45.807	C	46.940	44.937	45.749	C	47.016	44.918	45.868
O	45.724	44.756	46.120	O	45.682	44.817	46.061	O	45.766	44.726	46.278
C	44.350	42.379	45.784	C	44.377	42.381	45.797	C	44.373	42.366	45.771
O	45.322	41.716	45.439	O	45.379	41.749	45.473	O	45.383	41.747	45.452
N	43.812	42.397	47.022	N	43.828	42.397	47.033	N	43.793	42.350	46.990
H	42.988	42.968	47.163	H	42.981	42.941	47.153	H	42.935	42.877	47.101
C	44.123	41.365	47.999	C	44.135	41.368	48.014	C	44.126	41.342	47.984
H	45.084	40.944	47.690	H	45.099	40.947	47.714	H	45.098	40.939	47.684
H	43.368	40.569	47.951	H	43.382	40.570	47.963	H	43.391	40.528	47.944
C	44.232	41.837	49.432	C	44.232	41.839	49.449	C	44.216	41.838	49.410
C	43.719	41.033	50.457	C	43.703	41.040	50.469	C	43.682	41.060	50.443
H	43.151	40.139	50.208	H	43.128	40.151	50.216	H	43.110	40.165	50.204
C	43.924	41.370	51.794	C	43.898	41.375	51.809	C	43.868	41.425	51.776
H	43.539	40.726	52.580	H	43.497	40.735	52.591	H	43.464	40.801	52.568
C	44.637	42.524	52.120	C	44.619	42.520	52.142	C	44.588	42.578	52.089
H	44.798	42.800	53.160	H	44.773	42.796	53.184	H	44.737	42.873	53.126
C	45.120	43.338	51.096	C	45.120	43.330	51.124	C	45.094	43.366	51.056
H	45.661	44.248	51.337	H	45.668	44.235	51.373	H	45.635	44.279	51.284
C	44.916	43.009	49.758	C	44.924	43.004	49.784	C	44.905	43.010	49.723
H	45.260	43.677	48.972	H	45.278	43.672	49.003	H	45.264	43.662	48.930

Table S25. Cartesian coordinates (in Å) of QM atoms corresponding to the hydrolysis of metagenome-derived urethanase UMG-SP-2 with 4-nitrophenyl benzylcarbamate (pNC) substrate, optimized at M06-2X/6-31+G(d,p)/MM level of theory.

RC(H)				TS1(H) (νi = 299.7i cm^{-1})				Int1(H)			
Atoms	x	y	z	Atoms	x	y	z	Atoms	x	y	z
C	44.433	51.436	39.609	C	44.442	51.453	39.627	C	44.433	51.436	39.609
H	45.487	51.464	39.315	H	45.497	51.494	39.336	H	45.487	51.464	39.315
H	43.937	50.769	38.894	H	43.956	50.778	38.913	H	43.937	50.769	38.894
C	44.295	50.848	41.018	C	44.317	50.861	41.039	C	44.295	50.848	41.018
H	44.808	51.476	41.756	H	44.830	51.488	41.776	H	44.808	51.476	41.756
H	43.235	50.827	41.296	H	43.260	50.823	41.324	H	43.235	50.827	41.296
C	44.845	49.424	41.134	C	44.890	49.440	41.134	C	44.845	49.424	41.134
H	44.335	48.785	40.403	H	44.381	48.805	40.399	H	44.335	48.785	40.403
H	45.912	49.404	40.878	H	45.955	49.444	40.870	H	45.912	49.404	40.878
C	44.646	48.836	42.537	C	44.716	48.837	42.528	C	44.646	48.836	42.537
H	43.620	49.018	42.871	H	43.691	48.939	42.890	H	43.620	49.018	42.871
H	45.313	49.344	43.242	H	45.389	49.310	43.246	H	45.313	49.344	43.242
N	44.886	47.386	42.646	N	45.013	47.368	42.547	N	44.886	47.386	42.646
H	45.877	47.188	42.476	H	46.016	47.182	42.386	H	45.877	47.188	42.476
H	44.373	46.922	41.888	H	44.477	46.895	41.802	H	44.373	46.922	41.888
C	40.150	44.148	44.759	C	40.108	44.132	44.743	C	40.150	44.148	44.759
O	39.981	42.945	44.506	O	39.910	42.932	44.492	O	39.981	42.945	44.506
N	41.329	44.760	44.646	N	41.303	44.712	44.627	N	41.329	44.760	44.646
H	42.102	44.154	44.416	H	42.067	44.068	44.465	H	42.102	44.154	44.416
C	41.612	46.169	44.914	C	41.627	46.111	44.914	C	41.612	46.169	44.914
H	40.826	46.503	45.597	H	40.841	46.460	45.592	H	40.826	46.503	45.597
C	42.945	46.314	45.624	C	42.951	46.208	45.663	C	42.945	46.314	45.624
H	42.988	47.326	46.035	H	42.968	47.169	46.190	H	42.988	47.326	46.035
H	43.016	45.600	46.458	H	43.020	45.406	46.415	H	43.016	45.600	46.458
O	44.044	46.050	44.780	O	44.057	46.111	44.792	O	44.044	46.050	44.780

H	44.268	46.707	44.011	H	44.696	46.937	43.460	H	44.268	46.707	44.011
C	42.777	40.093	42.563	C	42.834	40.081	42.534	C	42.777	40.093	42.563
O	41.716	40.062	41.943	O	41.800	40.028	41.863	O	41.716	40.062	41.943
N	43.542	41.199	42.636	N	43.587	41.191	42.622	N	43.542	41.199	42.636
H	44.467	41.103	43.036	H	44.450	41.130	43.151	H	44.467	41.103	43.036
C	43.024	42.576	42.496	C	43.038	42.555	42.488	C	43.024	42.576	42.496
H	42.027	42.571	42.958	H	42.033	42.522	42.933	H	42.027	42.571	42.958
C	43.949	43.486	43.260	C	43.919	43.460	43.309	C	43.949	43.486	43.260
H	43.706	44.531	43.088	H	43.648	44.505	43.157	H	43.706	44.531	43.088
H	44.995	43.331	43.002	H	44.975	43.341	43.068	H	44.995	43.331	43.002
O	43.786	43.290	44.686	O	43.719	43.176	44.703	O	43.786	43.290	44.686
H	45.197	45.331	45.565	H	44.747	45.446	45.200	H	45.197	45.331	45.565
C	44.648	42.520	45.330	C	44.776	42.656	45.383	C	44.648	42.520	45.330
O	45.555	41.923	44.748	O	45.621	41.972	44.769	O	45.555	41.923	44.748
N	44.340	42.415	46.646	N	44.364	42.367	46.688	N	44.340	42.415	46.646
H	43.639	43.089	46.933	H	43.649	43.031	46.970	H	43.639	43.089	46.933
C	45.379	42.168	47.644	C	45.389	42.223	47.714	C	45.379	42.168	47.644
H	46.077	43.011	47.659	H	46.026	43.115	47.774	H	46.077	43.011	47.659
H	45.939	41.279	47.332	H	46.038	41.385	47.430	H	45.939	41.279	47.332
C	44.768	41.948	49.011	C	44.772	41.965	49.074	C	44.768	41.948	49.011
C	43.586	41.220	49.163	C	43.588	41.238	49.207	C	43.586	41.220	49.163
H	43.058	40.849	48.287	H	43.067	40.883	48.322	H	43.058	40.849	48.287
C	43.074	40.940	50.429	C	43.066	40.942	50.467	C	43.074	40.940	50.429
H	42.181	40.324	50.511	H	42.171	40.328	50.534	H	42.181	40.324	50.511
C	43.736	41.399	51.564	C	43.720	41.383	51.613	C	43.736	41.399	51.564
H	43.360	41.157	52.553	H	43.334	41.128	52.596	H	43.360	41.157	52.553
C	44.905	42.146	51.423	C	44.893	42.126	51.493	C	44.905	42.146	51.423
H	45.416	42.507	52.312	H	45.398	42.471	52.392	H	45.416	42.507	52.312
C	45.414	42.428	50.155	C	45.411	42.423	50.233	C	45.414	42.428	50.155
H	46.323	43.016	50.057	H	46.324	43.009	50.151	H	46.323	43.016	50.057
O	45.872	44.730	46.001	O	45.594	44.383	45.621	O	45.872	44.730	46.001
H	46.625	45.276	46.253	H	46.381	44.438	46.173	H	46.625	45.276	46.253

Int1(H)				TS2(H) (νi = 209.2i cm^{-1})				PC(H)			
Atoms	x	y	z	Atoms	x	y	z	Atoms	x	y	z
C	44.108	51.610	39.527	C	44.106	51.605	39.530	C	44.086	51.612	39.491
H	45.150	51.590	39.191	H	45.149	51.586	39.197	H	45.121	51.581	39.135
H	43.554	50.981	38.822	H	43.556	50.979	38.820	H	43.508	50.995	38.794
C	43.986	50.972	40.926	C	43.981	50.950	40.926	C	43.969	50.993	40.883
H	44.561	51.531	41.678	H	44.549	51.509	41.685	H	44.551	51.558	41.629
H	42.943	51.018	41.253	H	42.936	50.991	41.248	H	42.928	51.047	41.218
C	44.420	49.491	40.938	C	44.419	49.466	40.948	C	44.401	49.524	40.891
H	43.800	48.947	40.218	H	43.808	48.919	40.222	H	43.772	48.976	40.180
H	45.458	49.385	40.585	H	45.461	49.360	40.606	H	45.433	49.420	40.516
C	44.287	48.838	42.318	C	44.274	48.810	42.331	C	44.289	48.870	42.270
H	43.280	48.966	42.722	H	43.267	48.954	42.729	H	43.283	49.040	42.668
H	45.006	49.271	43.015	H	44.992	49.246	43.027	H	44.997	49.356	42.951
N	44.545	47.363	42.292	N	44.517	47.332	42.330	N	44.541	47.428	42.304
H	45.532	47.140	42.087	H	45.502	47.104	42.130	H	45.514	47.216	42.071
H	43.959	46.916	41.568	H	43.940	46.889	41.599	H	43.953	46.972	41.600
C	40.091	44.150	44.551	C	40.092	44.152	44.550	C	40.096	44.164	44.530
O	39.999	42.926	44.396	O	39.999	42.923	44.402	O	40.005	42.937	44.345
N	41.222	44.840	44.338	N	41.221	44.838	44.336	N	41.207	44.867	44.312
H	42.040	44.242	44.251	H	42.049	44.240	44.277	H	42.019	44.283	44.136
C	41.427	46.279	44.553	C	41.431	46.278	44.550	C	41.419	46.298	44.545
H	40.624	46.587	45.228	H	40.643	46.589	45.240	H	40.621	46.604	45.225
C	42.735	46.463	45.323	C	42.770	46.457	45.275	C	42.749	46.519	45.269
H	42.880	47.522	45.556	H	42.928	47.520	45.486	H	42.874	47.594	45.447
H	42.723	45.883	46.253	H	42.768	45.902	46.223	H	42.741	45.998	46.236
O	43.881	46.067	44.566	O	43.872	46.005	44.499	O	43.873	46.027	44.568
H	44.289	46.878	43.213	H	44.236	46.811	43.277	H	44.085	46.580	43.755
C	42.451	39.967	42.315	C	42.454	39.966	42.313	C	42.433	39.966	42.292
O	41.492	40.143	41.557	O	41.507	40.139	41.542	O	41.484	40.140	41.531

N	43.214	40.981	42.757	N	43.210	40.986	42.762	N	43.196	40.986	42.725
H	43.888	40.808	43.510	H	43.862	40.793	43.521	H	43.843	40.784	43.478
C	42.880	42.419	42.562	C	42.890	42.434	42.568	C	42.860	42.427	42.538
H	41.882	42.561	42.996	H	41.898	42.580	43.011	H	41.861	42.566	42.967
C	43.891	43.252	43.336	C	43.920	43.277	43.325	C	43.861	43.255	43.309
H	43.818	44.273	42.949	H	43.951	44.240	42.804	H	43.904	44.249	42.858
H	44.900	42.893	43.151	H	44.909	42.814	43.221	H	44.863	42.812	43.243
O	43.700	43.309	44.744	O	43.697	43.531	44.689	O	43.506	43.376	44.675
H	44.065	45.117	44.761	H	43.919	44.943	44.644	H	43.812	44.274	44.905
C	44.738	42.550	45.541	C	44.937	42.563	45.769	C	45.324	42.567	46.144
O	45.217	41.537	44.905	O	45.348	41.610	45.087	O	45.578	41.616	45.420
N	44.021	42.370	46.812	N	44.047	42.429	46.891	N	44.528	42.556	47.222
H	43.065	42.087	46.619	H	43.092	42.619	46.613	H	44.286	43.441	47.651
C	44.691	41.439	47.722	C	44.194	41.213	47.695	C	43.868	41.376	47.735
H	45.767	41.614	47.628	H	45.199	40.818	47.512	H	44.446	40.511	47.394
H	44.515	40.389	47.439	H	43.482	40.430	47.401	H	42.850	41.281	47.338
C	44.280	41.638	49.169	C	44.040	41.492	49.181	C	43.852	41.484	49.244
C	43.051	41.167	49.642	C	42.949	41.010	49.903	C	42.741	41.084	49.981
H	42.374	40.651	48.966	H	42.188	40.417	49.404	H	41.893	40.631	49.475
C	42.688	41.313	50.981	C	42.831	41.247	51.274	C	42.722	41.237	51.365
H	41.739	40.908	51.320	H	41.977	40.849	51.813	H	41.855	40.902	51.925
C	43.554	41.935	51.880	C	43.814	41.963	51.949	C	43.805	41.808	52.027
H	43.284	42.033	52.928	H	43.734	42.132	53.018	H	43.789	41.923	53.105
C	44.782	42.407	51.423	C	44.912	42.447	51.235	C	44.917	42.213	51.292
H	45.474	42.878	52.117	H	45.695	42.995	51.755	H	45.771	42.658	51.796
C	45.138	42.262	50.080	C	45.020	42.221	49.865	C	44.947	42.043	49.912
H	46.098	42.637	49.734	H	45.871	42.614	49.313	H	45.818	42.356	49.343
O	45.752	43.546	45.831	O	45.822	43.608	45.981	O	45.932	43.771	45.949
H	45.416	44.078	46.574	H	45.447	44.178	46.677	H	45.667	44.453	46.591

Table S26. Cartesian coordinates (in Å) of QM atoms corresponding to the decarboxylation of metagenome-derived urethanase UMG-SP-2 by the substrate, optimized at M06-2X/6-31+G(d,p)/MM level of theory.

RC(D)				TS1(D) (vi = 427.0i cm^{-1})				Int1(D)			
Atoms	x	y	z	Atoms	x	y	z	Atoms	x	y	z
C	44.295	51.518	39.558	C	44.325	51.534	39.575	C	44.312	51.541	39.5800
H	45.346	51.499	39.252	H	45.383	51.544	39.292	H	45.373	51.548	39.3126
H	43.759	50.865	38.860	H	43.817	50.863	38.875	H	43.809	50.871	38.8759
C	44.142	50.954	40.972	C	44.160	50.997	40.995	C	44.123	51.013	40.9979
H	44.688	51.561	41.702	H	44.673	51.632	41.725	H	44.618	51.653	41.7361
H	43.083	50.979	41.257	H	43.097	50.991	41.258	H	43.055	50.996	41.2436
C	44.635	49.510	41.069	C	44.706	49.578	41.104	C	44.687	49.605	41.1046
H	44.106	48.910	40.319	H	44.227	48.963	40.332	H	44.219	48.987	40.3280
H	45.704	49.460	40.819	H	45.783	49.579	40.896	H	45.765	49.625	40.9016
C	44.415	48.884	42.447	C	44.456	48.941	42.462	C	44.444	48.958	42.4537
H	43.371	49.016	42.754	H	43.406	49.029	42.756	H	43.392	49.007	42.7476
H	45.042	49.391	43.188	H	45.071	49.408	43.236	H	45.048	49.421	43.2383
N	44.709	47.441	42.505	N	44.768	47.489	42.444	N	44.799	47.512	42.3989
H	45.701	47.278	42.307	H	45.773	47.320	42.293	H	45.809	47.366	42.2413
H	44.182	46.980	41.754	H	44.249	47.037	41.678	H	44.277	47.058	41.6342
C	40.278	44.301	44.849	C	40.270	44.299	44.828	C	40.272	44.302	44.8315
O	40.205	43.066	44.720	O	40.183	43.067	44.681	O	40.199	43.075	44.6703
N	41.375	45.008	44.599	N	41.377	44.997	44.587	N	41.374	45.017	44.6006
H	42.212	44.449	44.437	H	42.232	44.449	44.440	H	42.210	44.468	44.4364
C	41.514	46.454	44.758	C	41.513	46.445	44.754	C	41.494	46.461	44.7673
H	40.723	46.755	45.450	H	40.730	46.741	45.455	H	40.694	46.750	45.4504
C	42.841	46.786	45.428	C	42.856	46.771	45.391	C	42.812	46.808	45.4355
H	42.890	47.875	45.492	H	42.880	47.850	45.544	H	42.846	47.883	45.5802
H	42.881	46.364	46.442	H	42.954	46.272	46.369	H	42.923	46.301	46.4040
O	43.981	46.299	44.751	O	43.955	46.412	44.586	O	43.932	46.494	44.6252

H	44.229	46.801	43.871	H	44.407	47.024	43.408	H	44.483	47.063	43.3168
C	42.626	40.288	42.603	C	42.665	40.294	42.582	C	42.668	40.299	42.5820
O	41.590	40.283	41.941	O	41.659	40.278	41.868	O	41.682	40.274	41.8374
N	43.366	41.399	42.808	N	43.398	41.407	42.802	N	43.389	41.415	42.8186
H	44.298	41.279	43.187	H	44.274	41.309	43.304	H	44.204	41.335	43.4210
C	42.962	42.806	42.558	C	43.004	42.822	42.559	C	43.001	42.823	42.5627
H	41.983	42.924	43.035	H	42.041	42.958	43.060	H	42.039	42.982	43.0618
C	43.994	43.702	43.201	C	44.066	43.747	43.139	C	44.068	43.741	43.1296
H	43.918	44.694	42.747	H	44.008	44.681	42.564	H	43.993	44.696	42.5992
H	44.991	43.314	42.956	H	45.046	43.305	42.918	H	45.051	43.318	42.9347
O	43.855	43.808	44.606	O	43.961	44.044	44.505	O	43.941	44.018	44.5220
H	44.038	44.781	44.796	H	43.993	45.258	44.553	H	44.018	45.493	44.6200
C	45.364	42.509	45.989	C	45.192	42.405	45.979	C	45.204	42.285	45.9774
O	45.838	42.077	44.929	O	45.625	41.912	44.923	O	45.430	41.737	44.8409
N	44.296	41.964	46.625	N	44.364	41.748	46.812	N	44.317	41.662	46.8323
H	43.673	41.556	45.936	H	44.160	40.796	46.556	H	43.829	40.892	46.4053
C	43.565	42.709	47.673	C	43.485	42.389	47.793	C	43.542	42.426	47.8107
H	42.512	42.443	47.550	H	42.492	41.946	47.673	H	42.500	42.102	47.7268
H	43.664	43.780	47.468	H	43.421	43.442	47.510	H	43.599	43.480	47.5211
C	44.001	42.383	49.086	C	43.971	42.229	49.213	C	44.010	42.257	49.2368
C	43.219	41.534	49.875	C	43.279	41.415	50.111	C	43.291	41.458	50.1293
H	42.318	41.088	49.460	H	42.392	40.876	49.785	H	42.402	40.930	49.7902
C	43.587	41.233	51.185	C	43.736	41.260	51.419	C	43.716	41.310	51.4483
H	42.971	40.564	51.776	H	43.198	40.618	52.108	H	43.153	40.685	52.1339
C	44.749	41.779	51.727	C	44.890	41.918	51.837	C	44.869	41.959	51.8862
H	45.032	41.554	52.751	H	45.230	41.815	52.864	H	45.181	41.870	52.9235
C	45.538	42.625	50.945	C	45.592	42.723	50.937	C	45.599	42.746	50.9926
H	46.444	43.065	51.352	H	46.490	43.249	51.249	H	46.493	43.268	51.3220
C	45.163	42.935	49.638	C	45.132	42.886	49.633	C	45.170	42.902	49.6761
H	45.777	43.610	49.050	H	45.675	43.520	48.936	H	45.733	43.520	48.9804
O	45.913	43.525	46.652	O	45.516	43.630	46.374	O	45.663	43.408	46.3001
H	46.611	43.928	46.110	H	45.206	44.153	45.572	H	44.767	43.878	45.0686

Int1(D)				TS2(D) (νi = 433.2i cm^{-1})				Int2(D)			
Atoms	x	y	z	Atoms	x	y	z	Atoms	x	y	z
C	44.273	51.547	39.566	C	44.261	51.540	39.566	C	44.282	51.547	39.561
H	45.334	51.535	39.303	H	45.322	51.516	39.301	H	45.341	51.541	39.285
H	43.762	50.902	38.843	H	43.746	50.900	38.841	H	43.773	50.895	38.843
C	44.061	50.979	40.967	C	44.047	50.957	40.965	C	44.097	50.969	40.963
H	44.582	51.576	41.722	H	44.560	51.554	41.725	H	44.608	51.580	41.716
H	42.993	50.992	41.214	H	42.978	50.967	41.207	H	43.031	50.966	41.218
C	44.570	49.543	41.030	C	44.554	49.517	41.042	C	44.625	49.539	41.050
H	44.050	48.956	40.262	H	44.042	48.925	40.274	H	44.134	48.934	40.277
H	45.640	49.523	40.786	H	45.626	49.490	40.808	H	45.701	49.524	40.832
C	44.358	48.888	42.383	C	44.325	48.871	42.402	C	44.382	48.883	42.406
H	43.309	48.916	42.690	H	43.273	48.922	42.696	H	43.325	48.973	42.680
H	44.965	49.363	43.158	H	44.922	49.363	43.175	H	44.969	49.392	43.178
N	44.747	47.447	42.328	N	44.694	47.429	42.399	N	44.723	47.450	42.436
H	45.759	47.325	42.154	H	45.703	47.297	42.238	H	45.726	47.318	42.258
H	44.222	46.983	41.572	H	44.185	46.958	41.639	H	44.220	46.984	41.675
C	40.274	44.366	44.862	C	40.281	44.363	44.878	C	40.311	44.376	44.894
O	40.222	43.129	44.779	O	40.231	43.120	44.835	O	40.277	43.132	44.863
N	41.360	45.080	44.562	N	41.363	45.068	44.558	N	41.393	45.087	44.584
H	42.196	44.536	44.366	H	42.196	44.516	44.360	H	42.220	44.538	44.365
C	41.504	46.522	44.711	C	41.520	46.515	44.705	C	41.529	46.537	44.705
H	40.718	46.829	45.403	H	40.749	46.819	45.416	H	40.753	46.840	45.409
C	42.830	46.861	45.380	C	42.877	46.820	45.335	C	42.873	46.894	45.334
H	42.900	47.946	45.455	H	42.941	47.906	45.443	H	42.932	47.986	45.346
H	42.891	46.420	46.384	H	42.933	46.368	46.341	H	42.927	46.521	46.367
O	43.970	46.449	44.639	O	43.971	46.367	44.575	O	43.995	46.369	44.658
H	44.469	46.987	43.248	H	44.362	46.950	43.392	H	44.260	46.847	43.748
C	42.684	40.272	42.624	C	42.682	40.273	42.627	C	42.675	40.284	42.624
O	41.682	40.266	41.902	O	41.668	40.269	41.923	O	41.652	40.290	41.935

N	43.433	41.367	42.844	N	43.435	41.368	42.838	N	43.437	41.374	42.831
H	44.220	41.275	43.480	H	44.255	41.263	43.422	H	44.284	41.256	43.373
C	43.026	42.772	42.630	C	43.036	42.782	42.635	C	43.042	42.789	42.631
H	42.065	42.904	43.143	H	42.078	42.904	43.155	H	42.088	42.914	43.156
C	44.089	43.650	43.267	C	44.107	43.664	43.269	C	44.110	43.669	43.255
H	44.095	44.604	42.736	H	44.122	44.599	42.702	H	44.096	44.634	42.742
H	45.079	43.194	43.180	H	45.090	43.190	43.155	H	45.100	43.225	43.100
O	43.853	43.989	44.625	O	43.909	43.996	44.629	O	43.922	43.890	44.646
H	44.051	45.454	44.756	H	44.003	45.140	44.668	H	44.058	44.886	44.770
C	45.267	42.327	46.228	C	45.314	42.320	46.279	C	45.255	42.378	46.185
O	45.317	41.652	45.144	O	45.298	41.641	45.234	O	45.310	41.642	45.192
N	43.911	42.433	46.734	N	43.848	42.612	46.719	N	43.735	42.340	46.758
H	43.383	41.568	46.700	H	43.312	41.746	46.773	H	43.474	41.378	46.981
C	43.667	43.254	47.919	C	43.696	43.444	47.936	C	43.463	43.239	47.910
H	42.598	43.504	47.917	H	42.654	43.780	47.957	H	42.384	43.419	47.930
H	44.239	44.175	47.774	H	44.351	44.305	47.789	H	43.986	44.170	47.683
C	44.036	42.637	49.252	C	44.050	42.725	49.218	C	43.939	42.650	49.216
C	43.192	41.686	49.836	C	43.211	41.722	49.713	C	43.188	41.648	49.843
H	42.294	41.367	49.310	H	42.323	41.425	49.156	H	42.277	41.274	49.378
C	43.488	41.138	51.082	C	43.494	41.089	50.922	C	43.595	41.121	51.067
H	42.824	40.392	51.506	H	42.840	40.297	51.273	H	43.008	40.335	51.531
C	44.631	41.547	51.770	C	44.614	41.470	51.661	C	44.752	41.600	51.682
H	44.851	41.136	52.751	H	44.826	40.991	52.614	H	45.056	41.205	52.647
C	45.486	42.482	51.185	C	45.467	42.454	51.161	C	45.516	42.580	51.048
H	46.386	42.799	51.705	H	46.354	42.741	51.716	H	46.428	42.949	51.507
C	45.197	43.022	49.931	C	45.194	43.075	49.942	C	45.116	43.103	49.818
H	45.875	43.739	49.476	H	45.875	43.827	49.552	H	45.724	43.857	49.326
O	46.136	42.990	46.783	O	46.161	42.853	46.960	O	45.983	43.102	46.811
H	43.723	43.252	45.295	H	43.588	43.162	45.808	H	43.218	42.656	45.923

Int2(D)				TS3(D) (vi = 208.5i cm^{-1})				PC(D)			
Atoms	x	y	z	Atoms	x	y	z	Atoms	x	y	z
C	44.292	51.547	39.568	C	44.300	51.548	39.565	C	44.300	51.546	39.562
H	45.353	51.542	39.302	H	45.360	51.547	39.291	H	45.355	51.542	39.273
H	43.787	50.895	38.847	H	43.795	50.893	38.847	H	43.783	50.892	38.852
C	44.091	50.975	40.970	C	44.116	50.970	40.969	C	44.133	50.970	40.969
H	44.592	51.591	41.725	H	44.625	51.584	41.720	H	44.663	51.576	41.712
H	43.021	50.971	41.213	H	43.049	50.965	41.223	H	43.071	50.981	41.242
C	44.621	49.548	41.067	C	44.645	49.540	41.062	C	44.641	49.532	41.054
H	44.136	48.939	40.293	H	44.157	48.931	40.290	H	44.128	48.931	40.292
H	45.699	49.537	40.853	H	45.722	49.525	40.848	H	45.714	49.499	40.818
C	44.377	48.894	42.424	C	44.399	48.887	42.421	C	44.413	48.884	42.419
H	43.319	48.978	42.696	H	43.343	48.980	42.695	H	43.363	48.993	42.712
H	44.959	49.407	43.197	H	44.987	49.396	43.192	H	45.022	49.387	43.178
N	44.727	47.463	42.455	N	44.736	47.452	42.456	N	44.731	47.444	42.452
H	45.728	47.336	42.271	H	45.736	47.318	42.269	H	45.728	47.301	42.252
H	44.223	46.994	41.694	H	44.228	46.986	41.698	H	44.210	46.986	41.698
C	40.308	44.346	44.878	C	40.306	44.345	44.872	C	40.302	44.343	44.865
O	40.260	43.102	44.828	O	40.250	43.104	44.805	O	40.241	43.105	44.781
N	41.399	45.051	44.589	N	41.404	45.047	44.596	N	41.403	45.046	44.596
H	42.228	44.498	44.382	H	42.233	44.488	44.412	H	42.225	44.487	44.396
C	41.543	46.498	44.722	C	41.547	46.497	44.723	C	41.547	46.496	44.724
H	40.765	46.801	45.425	H	40.771	46.802	45.428	H	40.770	46.800	45.428
C	42.884	46.850	45.360	C	42.889	46.847	45.361	C	42.887	46.839	45.368
H	42.932	47.940	45.401	H	42.943	47.938	45.390	H	42.951	47.929	45.395
H	42.940	46.450	46.384	H	42.942	46.456	46.386	H	42.935	46.448	46.393
O	44.015	46.355	44.674	O	44.017	46.335	44.680	O	44.013	46.314	44.689
H	44.264	46.846	43.770	H	44.267	46.826	43.783	H	44.262	46.807	43.798
C	42.681	40.282	42.621	C	42.676	40.282	42.626	C	42.666	40.282	42.632
O	41.659	40.280	41.931	O	41.647	40.285	41.947	O	41.637	40.284	41.956
N	43.441	41.374	42.822	N	43.438	41.375	42.824	N	43.424	41.378	42.830

H	44.296	41.256	43.353	H	44.321	41.248	43.299	H	44.309	41.251	43.301
C	43.040	42.786	42.620	C	43.031	42.788	42.622	C	43.014	42.788	42.625
H	42.083	42.906	43.142	H	42.074	42.900	43.143	H	42.053	42.900	43.139
C	44.102	43.671	43.244	C	44.087	43.678	43.246	C	44.070	43.683	43.238
H	44.075	44.642	42.741	H	44.027	44.661	42.771	H	43.974	44.676	42.789
H	45.096	43.241	43.084	H	45.083	43.275	43.030	H	45.059	43.304	42.962
O	43.911	43.875	44.637	O	43.941	43.830	44.651	O	43.992	43.801	44.654
H	44.070	44.862	44.775	H	44.083	44.811	44.809	H	44.085	44.788	44.821
C	45.294	42.383	46.133	C	45.551	42.521	45.936	C	45.789	42.656	45.743
O	45.353	41.665	45.130	O	45.558	41.788	44.991	O	45.734	41.893	44.853
N	43.767	42.291	46.723	N	43.702	42.074	46.801	N	43.465	41.969	46.883
H	43.551	41.324	46.971	H	43.505	41.129	47.127	H	42.873	41.186	47.159
C	43.466	43.200	47.859	C	43.365	43.035	47.867	C	43.313	43.011	47.916
H	42.383	43.349	47.875	H	42.278	43.176	47.918	H	42.259	43.304	48.038
H	43.962	44.142	47.619	H	43.824	43.988	47.586	H	43.862	43.895	47.579
C	43.956	42.645	49.174	C	43.889	42.571	49.206	C	43.858	42.550	49.249
C	43.222	41.648	49.827	C	43.183	41.600	49.925	C	43.143	41.614	50.006
H	42.313	41.252	49.376	H	42.267	41.178	49.514	H	42.205	41.212	49.628
C	43.643	41.151	51.058	C	43.638	41.162	51.167	C	43.617	41.185	51.243
H	43.070	40.369	51.545	H	43.081	40.405	51.708	H	43.052	40.457	51.815
C	44.799	41.655	51.654	C	44.809	41.694	51.708	C	44.817	41.690	51.747
H	45.116	41.283	52.624	H	45.152	41.367	52.685	H	45.173	41.369	52.721
C	45.548	42.629	50.994	C	45.530	42.644	50.986	C	45.545	42.607	50.991
H	46.460	43.018	51.436	H	46.449	43.060	51.388	H	46.485	43.006	51.363
C	45.132	43.124	49.758	C	45.074	43.083	49.742	C	45.069	43.036	49.751
H	45.728	43.873	49.245	H	45.644	43.825	49.191	H	45.644	43.757	49.176
O	46.001	43.113	46.774	O	45.992	43.328	46.662	O	46.007	43.398	46.603
H	43.232	42.570	45.889	H	43.141	42.271	45.970	H	43.070	42.338	46.015

Experimental evaluation of the effect of the concentrations of Triton X100 over the esterase activity of the UMG – SP2.

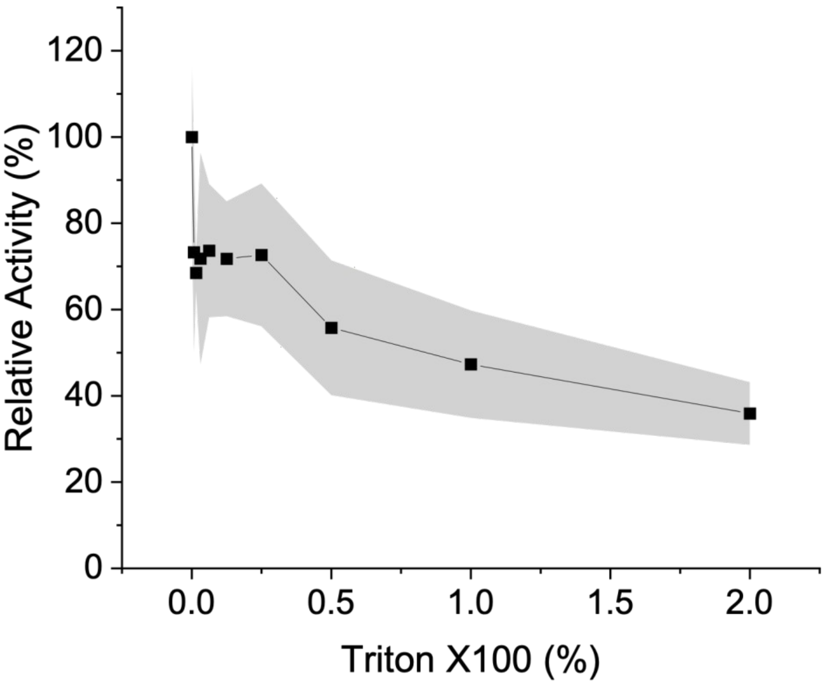

Figure S25. Effect of increasing concentrations of Triton X100 over the esterase activity of the UMG – SP2. Conditions: 0.5 mM of 4-nitrophenyl butyrate in buffer sodium phosphate 50 mM at pH 7 and 30º C. UMG-SP2 specific activity in the absence of Triton X100 33.11 U/mg

References

(1) Sievers, F.; Wilm, A.; Dineen, D.; Gibson, T. J.; Karplus, K.; Li, W.; Lopez, R.; McWilliam, H.; Remmert, M.; Söding, J.; Thompson, J. D.; Higgins, D. G. Fast, Scalable Generation of High-quality Protein Multiple Sequence Alignments Using Clustal Omega. *Mol Syst Biol* **2011**, *7* (1), 539. https://doi.org/https://doi.org/10.1038/msb.2011.75.

(2) Eisenberg, D.; Schwarz, E.; Komaromy, M.; Wall, R. Analysis of Membrane and Surface Protein Sequences with the Hydrophobic Moment Plot. *J Mol Biol* **1984**, *179* (1), 125–142. https://doi.org/https://doi.org/10.1016/0022-2836(84)90309-7.
